# Distributive randomization: a pragmatic fractional factorial design to screen or evaluate multiple simultaneous interventions in a clinical trial

**DOI:** 10.1186/s12874-024-02191-9

**Published:** 2024-03-11

**Authors:** Skerdi Haviari, France Mentré

**Affiliations:** 1 Université Paris Cité, Inserm, IAME, Paris, 75018 France; 2 Département Epidémiologie Biostatistiques Et Recherche Clinique, AP-HP, Hôpital Bichat, Paris, 75018 France

**Keywords:** Trial design, Methodology, Simulation

## Abstract

**Background:**

In some medical indications, numerous interventions have a weak presumption of efficacy, but a good track record or presumption of safety. This makes it feasible to evaluate them simultaneously. This study evaluates a pragmatic fractional factorial trial design that randomly allocates a pre-specified number of interventions to each participant, and statistically tests main intervention effects. We compare it to factorial trials, parallel-arm trials and multiple head-to-head trials, and derive some good practices for its design and analysis.

**Methods:**

We simulated various scenarios involving 4 to 20 candidate interventions among which 2 to 8 could be simultaneously allocated. A binary outcome was assumed. One or two interventions were assumed effective, with various interactions (positive, negative, none). Efficient combinatorics algorithms were created. Sample sizes and power were obtained by simulations in which the statistical test was either difference of proportions or multivariate logistic regression Wald test with or without interaction terms for adjustment, with Bonferroni multiplicity-adjusted alpha risk for both. Native R code is provided without need for compiling or packages.

**Results:**

Distributive trials reduce sample sizes 2- to sevenfold compared to parallel arm trials, and increase them 1- to twofold compared to factorial trials, mostly when fewer allocations than for the factorial design are possible. An unexpectedly effective intervention causes small decreases in power (< 10%) if its effect is additive, but large decreases (possibly down to 0) if not, as for factorial designs. These large decreases are prevented by using interaction terms to adjust the analysis, but these additional estimands have a sample size cost and are better pre-specified. The issue can also be managed by adding a true control arm without any intervention.

**Conclusion:**

Distributive randomization is a viable design for mass parallel evaluation of interventions in constrained trial populations. It should be introduced first in clinical settings where many undercharacterized interventions are potentially available, such as disease prevention strategies, digital behavioral interventions, dietary supplements for chronic conditions, or emerging diseases. Pre-trial simulations are recommended, for which tools are provided.

**Supplementary Information:**

The online version contains supplementary material available at 10.1186/s12874-024-02191-9.

## Introduction

As modern medicine expands, medical management options are constantly growing; for example, more drugs are approved [1, 2] than withdrawn [3], and thus the total available number is increasing. The same is true for medical devices [4], supplements [5] and even more so for non-pharmacological, non-device interventions (that can hardly be withdrawn in any meaningful sense). Thus, if only by repurposing, available options will inevitably come to include weak ones, with little pre-clinical evidence base and no randomized trials. This is especially true in areas such as chronic disease, functional symptoms, lifestyle interventions, and/or prevention strategies (e.g. different diet/supplement/exercise/meditation/education types). This can also be true when a new disease or disease form emerges (e.g. long COVID). If many possible interventions exist, and none of them are too burdensome or dangerous, it becomes feasible to administer them simultaneously, including for the purpose of evaluating them in a trial, in order to quickly generate strong evidence. In the aforementioned example of long COVID, with its lack of animal models and unknown but likely immune-mediated pathophysiology [6], this could take the form of combinations of immunosuppressants or immunomodulators, of which more than 70 are approved [7]. Many other immune-mediated diseases without good etiological animal models exist. In these cases, evaluating all interesting candidates would likely require a massive clinical research effort, one that would be accelerated if participants can take several at once during the trial.

One way to do this is using factorial trials: if there are K interventions, and each of them can be administered independently, 2^ K^ combinations are defined and can each be tested. While extensively used for industrial or non-clinical research experiments, this approach is rarely used in clinical trials beyond a 2 × 2 factorial design (with 4 arms) [8]. Many reasons participate to this underuse: among them, it can be logistically complicated to manage too many different unique combinations in many different patients, it can be unsafe to administer too many interventions per patient (in terms of medical management of toxicities), and it can be difficult to have patients and clinicians accept that some participants will get most promising investigational agents while others will get all placebos (a problem that is already present with sham- and placebo-controlled two-arm trials, but even more salient with many investigational interventions or agents). In the example of long COVID above, all these issues would make a full factorial design challenging, even if the number of candidates could be extensively reduced by observational or pre-clinical studies. In this simulation study, examining cases where K is relatively large (4 and above), we introduce a limit k to the number of interventions that can be administered simultaneously in each patient, and explore the most natural way to put it in practice, which is to administer k interventions to each patient, picked at random among all candidates.

**Fig. 1:**
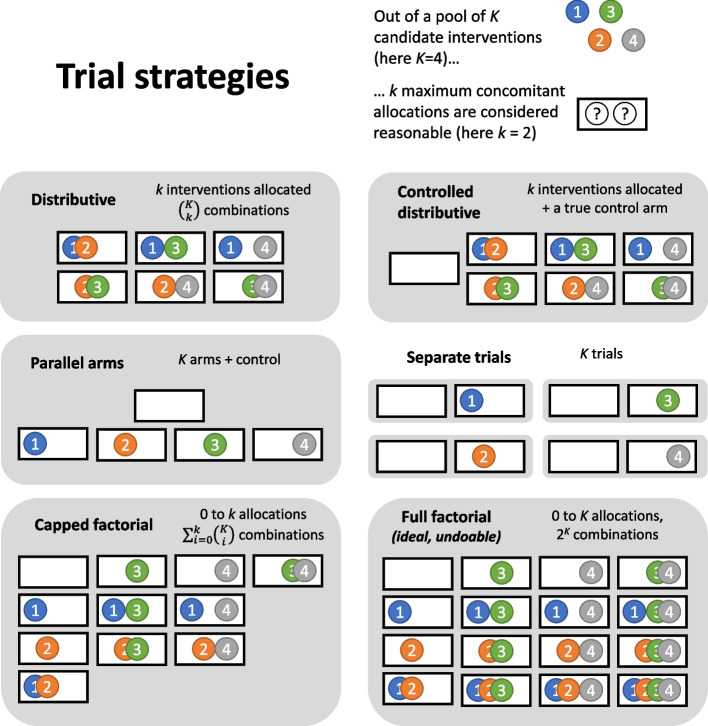
Distributive trials versus other possible designs for parallel evaluation. The situation shown is that of 4 candidate interventions, numbered 1 to 4 and color-coded, with 2 maximum interventions in each patient due to toxicity or participant burden. Each white rectangle represents a possible combination of allocations in one patient (all possibilities are shown), and each gray rectangle represents a trial

The basis for the limit k can vary across settings: it can be physiological (fear of combining too many drugs for a new pathogen), ergonomic (avoid introducing too many changes to a digital interface or exercise routine), or even economic (in cases where participants incur costs or burden for each intervention). Using the $\left({K \atop k}\right)$ notation for the binomial coefficient, this creates $\left({K \atop k}\right)$ different arms with k interventions each. We call this distributive randomization, and compare distributive trials based on it to full factorial trials, "capped" factorial trials where combinations with more than k interventions are excluded, parallel-arm trials, wholly separate trials, and a version of distributive trials with an additional true control arm without any intervention (Fig. 1).

Distributive randomization can be seen as a particular type of fractional factorial experimental plan. The fractional factorial design starts from a full factorial design matrix (with all combinations of all factors to be tested), and strategically omits some. Fractional factorial plans are often used in biotechnology, chemistry or agrotechnology, most typically to cap the total number of experimental combinations to be prepared, at the cost of not being able to explore some higher-order interactions [9]. In the case of clinical research, because each participant is seen and managed individually, this classical use of the design may be less interesting. As for the practical use of fractionation in clinical research, a quick review of the literature using the "fractional factorial" expression in the NCBI Pubmed database found only 13 trials employing this terminology [10–22], none of them in order to cap interventions per patient. In theory, fractionation strategies can have the latter goal, but we have been unable to find simulation-tested algorithms for clinical research methodologists, whose work impacts human subjects and requires multiple institutional reviews, and thus may face higher adoption barriers. Therefore, this work aims to be a starting point in that regard.

In addition to the randomization strategy, analysis of the results is not obvious. In a factorial trial, each intervention is evaluated in all treated versus all non-treated patients with this intervention, and an adjustment can be made for multiplicity (testing several hypotheses at the same time), either explicitly or by using a test that takes it into account (such as Tukey’s test) [23, 24]. The same can be done for parallel arms, which is often done with Dunnett's test. An adjustment could even theoretically be used for separate trials, although there is no practical way to do so across studies. In the present study, we apply a multiplicity adjustment consistently (always), in order to ensure comparability. For the statistical test, we start with a simple difference of proportions. In cases where multiple efficacious interventions and/or interactions are expected, we use linear or logistic regression [25], which provides more flexibility to focus on the most relevant pre-specified estimands.

## Materials and methods

### Combinatorics for allocation table computation

Because of the high number of treatment combinations and the need find sample sizes for intricate cases (including ones where the design matrix has > 100 k rows), we needed efficient combinatorics algorithms to reduce the set of explicitly computed combinatorial allocations, and obtain an allocation table for simulations efficiently.

To this end, distributive randomization was computed as a fractionation of a factorial design. In the latter, 2^ K^ combinations are initially possible, each allocated with probabilities

$${\prod }_{i=1}^{K}\left[{{{\text{X}}}_{{\text{i}}}{\text{p}}}_{{\text{i}}}+({1-{\text{X}}}_{{\text{i}}})({1-{\text{p}}}_{{\text{i}}})\right]$$

where X_i_ indicates the allocation of intervention i, and p_i_ is the intervention’s allocation probability, with p_i_ = 0.5 for balanced allocations and p_i_ ≠ 0.5 for unbalanced allocations. For the fractionation, only those combinations with k allocations are kept (there are $\left({K \atop k}\right)$ of those), and probabilities are normalized to sum to 1. This is equivalent to a situation where patients with more or less than k interventions would be rerandomized until they received k interventions.

Even with this reduced set, the design matrix can quickly become too large. To further reduce the number of explicitly computed combinations for simulations and sample size determinations, some combinations were kept implicit. For this, the following reasoning was used: “interesting” interventions were defined, as either non-null efficacy and/or unbalanced allocations, and the algorithm focused on all combinations of those, whereas "uninteresting" combinations, being interchangeable (balanced and null efficacy), were simply appended at the end of calculations.

First, for the subset of L interesting interventions, the full table with all combinations was computed, with the corresponding probability for each of the 2^L^ combinations (as a partial draw) explicitly computed (and their success rates appended). Formally, if we number interventions so that 1 to L denotes interesting ones and L + 1 to K denotes uninteresting ones, we have for the subset of interesting interventions

$$\mathrm{p}'({{\text{X}}}_{{\text{I}}})={\prod }_{{\text{i}}=1}^{{\text{L}}}\left({{{\text{X}}}_{{\text{i}}}{\text{p}}}_{{\text{i}}}+({1-{\text{X}}}_{{\text{i}}})({1-{\text{p}}}_{{\text{i}}})\right)$$

where X_I_ = {X_1_,X_2_,X_3,_…,X_L_} is the vector of indicator variables for the allocations of interesting interventions, L is the number of interesting interventions and p_i_ is as above.

Then, because the draws for different candidate interventions are independent except for their total number, the probability of each of the 2^L^ combinations of interesting interventions in the final allocation is obtained, by multiplying the probability of that combination as a partial draw, p'(X_I_), by the probability of getting exactly the correct number of allocations among the remaining uninteresting interventions (by the binomial distribution with *p* = *q* = 0.5 by definition of uninteresting interventions), p''(X_I_), then normalizing to obtain the final probability of allocation, p(X_I_).

$${{\text{l}}}={\sum }_{{\text{i}}=1}^{{\text{L}}}{{\text{X}}}_{{\text{i}}}$$

$${{\text{p}}''({\text{X}}}_{{\text{I}}})=\left({{\text{K}}-{\text{L}} \atop {\text{k}}-{\text{l}}}\right){0.5}^{{\text{K}}-{\text{L}}}$$

$$\mathrm{p{}}\mathrm{^{}}{{'''}({\text{X}}}_{{\text{I}}})={\text{p}}{'}({{\text{X}}}_{{\text{I}}})\times \mathrm{ p{''}}({{\text{X}}}_{{\text{I}}})$$

$${\text{p}}\left({{\text{X}}}_{{\text{I}}}\right)=\frac{{\mathrm{p}}{'''}({{\text{X}}}_{{\text{I}}})}{\sum \mathrm{p}{'''}({{\text{X}}}_{{\text{I}}})}$$

The convention $\left({{\text{Y}} \atop {\text{y}}}\right)=0$ for y < 0 or y > Y is used when computing p''(X_I_) to give arms with too many or too few allocations a probability of 0. In the provided code, to enable others to explore cases where ineffective allocations have arbitrary allocation ratios (and thus are not equiprobable), all possible values of p''(X_I_) are pre-computed by explicitly drawing the (L + 1)^th^ to K^th^ interventions one by one and keeping track of the probabilities for each overall number of allocations.

For the capped factorial the computations were as above except ${{\text{p}}''({\text{X}}}_{{\text{I}}})={\sum }_{a=0}^{k}\left({{\text{K}}-{\text{L}} \atop {\text{a}}-{\text{l}}}\right){0.5}^{{\text{K}}-{\text{L}}}$ since fewer than k allocations are also allowed.

For the full factorial trial computations were as above except p''(X_I_) was set to 1 since any number of uninteresting interventions is allowed.

For the controlled distributive design, an arm with no interventions was added at the end of the computation, with a specified probability, and the other p(X_I_) terms were adjusted.

For parallel-arms each arm was computed explicitly and the allocation ratio was 1:1 between each intervention and control.

### Adding outcomes to the allocation table and simulating a trial

The probability of clinical success was computed for each of the 2^L^ combinations of interesting interventions (ignoring the uninteresting ones, which have no effect by definition). This was done by applying an explicit list of successful outcome probabilities, with a priority for specified higher-order combinations (e.g. if intervention 1 + intervention 2 combined had 99% success, and intervention 1 had 70% success, the former value was used for all arms containing 1 and 2, and the latter for all other arms containing 1). The list used in the scripts was written to avoid any ambiguous or implicit cases.

The full allocation table with outcomes allowed overall success probabilities by presence or absence of interesting interventions to be readily computed, and enabled fast simulations by drawing patients and their outcomes in the detailed combinatorial arms. To complete the design matrix with the uninteresting interventions, for each simulated patient the remaining draws were made:

- for distributive randomization, k-l uninteresting interventions among K-L were drawn at random
- for the full factorial design, interventions among the remaining K-L were drawn independently
- for the capped factorial design, in this study no simulations were run, so no algorithm was devised to complete the design matrix (in this case, it would need to keep track of the probability for each number of uninteresting interventions, and first draw that number, then their identity)

### Statistical risk, sample size and power computations

Type I (α) risk was set at bilateral 5% and type II (β) at 10%. For consistency, due to its generic nature and its applicability to all statistical tests, a Bonferroni adjustment was applied for multiplicity in all situations where the effect of several interventions was tested at the same time, regardless of the trial design. The correction was applied to the α threshold, which was divided by the number of tests. The corrected α risk was then used for sample size and power computations. The β risk was not adjusted: e.g. if several interventions were equally effective, β was the probability of missing the first one, regardless of the others. To evaluate α risk for multiple interventions, family-wise error rate (FWER) was computed as the proportion of trials that made at least one false rejection of a null hypothesis.

In the base case, only one intervention was considered effective, and therefore analytical formulas were used for sample size and power, based on difference of proportions under a Gaussian approximation [26–29], using the previously obtained table. The formula is

$${n}_{I}=\frac{{\left({z}_{\alpha }\sqrt{\left(r+1\right){p}_{I}\left(1-{p}_{I}\right)}+{z}_{\beta }\sqrt{r{{\text{p}}}_{I}\left(1-{p}_{I}\right){{\text{p}}}_{C}\left(1-{p}_{C}\right)}\right)}^{2}}{r{\left({{\text{p}}}_{I}-{p}_{C}\right)}^{2}}$$

$$N={n}_{I}+{rn}_{I}$$

where r is the ratio between subjects not receiving and receiving the tested intervention (given by the trial design), ${n}_{I}$ is the number of subjects receiving the tested intervention, r ${n}_{I}$ the number not receiving it, n is the total sample size, ${p}_{I}$ is the success probability for those receiving the intervention and ${p}_{C}$ for those not receiving it, and ${z}_{\alpha }$ and ${z}_{\beta }$ denote the standard normal values for the desired type I and type II errors.

In more complicated cases, where multiple interventions could be effective, with or without interactions, analysis was performed by logistic regression and simulations were used for trial size and power. Power computation was straightforward; using the previously derived table to draw allocations and outcomes for virtual patients, 5000 trials were simulated, and power was approximated as the frequency of a p-value below the α threshold for one pre-specified intervention with the desired effect size (and not any of the effective ones within a trial).

Trial sizes by simulation were obtained with a bespoke algorithm. The probability of discovery (rejection of the null) was modeled using a modified probit model with a single variable, the square root of the trial size. This specification was used because many closed-form sample size formulas are of the form ${\text{N}}={({{\text{z}}}_{1-\mathrm{\alpha }/2}+{{\text{z}}}_{1-\mathrm{\beta } })}^{2}/{\text{K}}$ where K is an expression of the effect size; expressing power as a function of sample size thus gives an equation of the form ${{\text{p}}}_{d}=\Phi ({\text{a}}+{\text{b}}\sqrt{{\text{N}}})$ with a and b as parameters to optimize, and p_*d*_ the probability of a discovery (rejection of the null).

The modification of the probit model was the use of the following reparameterization:

$${{\text{p}}}_{d}=\Phi \left({{\text{z}}}_{1-\mathrm{\beta } }+{\text{b}}({\text{X}}-{{\text{X}}}_{0})\right)$$

where X is the square root of the trial size, b is a nuisance slope parameter, X_0_ is the parameter of interest (the square root of the right trial size), and ${{\text{z}}}_{1-\mathrm{\beta } }$ ensures that when X is equal to X_0_, the desired power is obtained.

Sampling of trial sizes was started with one (assumed) non-significant trial with 1 patient, one (assumed) significant trial with 10^6^ patients, and 48 trials between 50 and 10,000 patients (with equal log-scale increments). The 48 initial trials were re-run if they were either all significant or all non-significant, then additional trials were simulated in batches of either 50 trials or 10% of already simulated trials (whichever highest), with new fits of the probit model after each batch, until at least 5000 simulations were run. Simulated sample sizes for each batch were spread uniformly in an interval of $\widehat{{{\text{X}}}_{0}}$ (current estimate) ± its standard error derived from the Hessian matrix, which narrowed at each step, or ± 10, whichever was smallest. This quickly resulted in sampling occurring only around the desired value, alleviating misspecification concerns.

As a validation step, computations of sample size for Fig. 3B (2 to 8 allocations among 3 to 20 interventions) were all checked with 5000 simulations each and showed an observed power of 90.02 ± 0.5857%, whereas 90 ± 0.5888% would be expected simply due to chance if starting from the known true value and adding Monte Carlo variances (first from the sample size algorithm, which usually stopped at 5400 simulations, then from the check with 5000). Outliers could very rarely occur (apparently due to wildly improbable first batches) and were re-run after being diagnosed visually (an implementation that requires b to be positive and "forgets" the first batches is available in the provided code and does not seem to have this issue).

### Assumptions and simulation scenarios

In all studied scenarios, the rate of successful outcomes was 50% in the absence of any intervention, and most interventions were ineffective, having no impact on the success rate. When shown, factorial trials used a 1:1 randomization, violating the individual-patient limit of k interventions of distributive trials for some of their patients, and are presented as an ideal but unrealistic benchmark for the envisioned clinical use cases.

Table 1 shows an overview of all scenarios and their progression. The overall goal is to examine the sample size and power of a distributive design, under several sparse parametric (logistic) efficacy assumptions, that are relatively simple and not based on real data. The presented set of simulations starts from a simple case with a single effective intervention that needs to be found in the trial, and shows sample size gains with distributive trials. We then move to a situation where this single-effective assumption is false, and another intervention is effective as well. This incorrect assumption leads to changes in power, that vary with the simulation truth interactions between the two. We then examine scenarios where the second effective intervention is correctly foreseen, to see how sensitive the distributive design is in terms of sample size. Finally, we try different design and analysis modifications, requiring decreasing levels of a priori precise suspicion of interactions, to detect all effective interventions with as few patients as possible. We also provide probabilities for false discoveries for most parameter sets in Supplementary Materials. Except for the FWER estimand, the number of simulations per set of conditions was set to at least 5000 as this number yields a Monte Carlo variance [30] of 0.1*0.9/5000 and standard error of 0.42% (< 0.5%) for the 90% power that is sought. For sample size, as noted above, computing power on the sample size produced by the bespoke algorithm above showed variance to be roughly doubled, as expected, meaning the actual powers of the proposed sample sizes apparently had the same Monte Carlo variance. For the FWER estimand, since the goal was to check it was controlled, 1000 simulations per condition were run, yielding confidence intervals of 1–3 percentage points (obtained by the Jeffreys method).

**Table 1:** Simulation and analysis scenarios

Scenario group	Designs	k	K	Interventions' probability of success			Analysis	Foresight^b^/Estimand	Figure
				1	2	1 + 2			
1	All	2	4	70%	50%	70%	Difference of proportions	Yes/Sample size	2A
		2	4–20						2BCD
		4							2E
		8							2F
2	Distributive, factorial	3–8	5, 10, 15, 20	70%	50%	50%	Logistic regression without interaction	Yes/Sample size	3A
		2, 4, 6, 8	4–20						3B
		2–8			70%	84.5%^a^		No/Power	3C
						99%			3D
						70%			3E
					60%	70%			3F
3	Distributive, factorial	2–8	4–20	70%	70%	84.5%^a^	Logistic regression without interaction	Yes/Sample size	4A
						99%			4B
						70%			4C
					60%	70%			4D
4	(controlled) Distributive with 0–80% intervention-free control	4	10	70%	70%	84.5%^a^	Difference of proportions, or logistic regression, with interaction either absent, or backward selected, or pre-specified, or gated pre-specified	Yes/Sample size	5A
						70%			5B
		2	20			84.5%^a^			5C
						70%			5D

50% success means no intervention effect (this is the baseline in all scenarios) ^a^denotes logit-scale additivity, i.e., expit(2ln(7/3)), which is rounded to 84.5% in the table and 84.48276% in the code implementation ^b^"foresight" denotes whether trial size was computed with true assumptions; if so, the quantity of interest is sample size, if not, the quantity of interest is power (i.e., how it is affected by those wrong assumptions), and in the latter case the wrong assumption is that of a single effective intervention with 70% success rate

Scenarios were devised in a progressive manner and are best understood as investigating questions arising from previous scenarios. Their details are given here.

In scenario group 1, only one intervention was assumed to possibly be effective, with successful outcomes going from the baseline 50% to 70% with that intervention (regardless of any other interventions in those patients). Sample sizes were obtained under closed-formed formulas for a difference of proportions with unbalanced arms shown above, pooling all patients who did not receive a particular intervention to serve as control for that intervention (trading power for a small bias against the other interventions in distributive designs). For parallel arm trials, a non-pooled analysis is also shown (each intervention versus the control arm), as it is the most intuitive and common analysis. For distributive and capped factorial designs, the number of simultaneously feasible interventions, denoted k, was either 2, 4 or 8, and the total number of candidate interventions in the trial, K, varied from k + 2 to 20.

In scenario group 2 the comparison was restricted to distributive versus full factorial designs. A first subset of this scenario group explored how k and K impacted sample size, with the same settings as above except for the following:

- the analysis relied on logistic regression (allowing detection of, and adjustment for, more than 1 effective intervention), with main effect terms only
  $${\text{logit}}\left({\text{p}}\left({{\text{Y}}}_{{\text{i}}}=1\right)\right)={\upalpha }+{\upbeta }_{1}{{\text{X}}}_{1,{\text{i}}}+{\upbeta }_{2}{{\text{X}}}_{2,{\text{i}}}+\dots +{\upbeta }_{{\text{K}}}{{\text{X}}}_{{\text{K}},{\text{i}}}$$

where Y_i_ is the indicator variable for success or failure for subject i, α is the intercept, β_j_ is the coefficient for intervention j, and X_j,i_ is the indicator variable for whether subject i received intervention j.

- the sample sizes were therefore obtained by simulation
- holding K fixed at 5, 10, 15 or 20, all possible values of k were tested to find the ideal k/K ratio for sample size minimization
- holding k fixed at 2, 4, 6 or 8, K was increased from k + 2 to 20 to examine the incremental cost of evaluating additional interventions (e.g. with weak presumption of efficacy) in terms of sample size

In a subset of scenario group 2, the simulation truth did not match the sample size assumption: a second intervention was effective, while only one was expected to be. The impact of this incorrect assumption on power was computed by simulation. The second intervention's efficacy, unplanned for at the sample size computation stage, was one of the following four:

- same main effect as the expected effective intervention (70% success when administered standalone, regardless of other interventions except the other intervention with a main effect), with additivity on the logit scale (the combination had an 84.48276% success rate)
- same main effect as the expected effective intervention, but strong synergy (99% success rate for the combination)
- same main effect as expected effective intervention, but no additivity of effects (the combination also has a 70% success rate)
- smaller main effect than the expected effective intervention (60% success rate rather than 70%), and no additivity (the combination also has a 70% success rate)

In scenario group 3 similar interactions as for scenario group 2 were used, except they were taken into account initially to compute trial size.

Finally, in scenario group 4, we explored additional strategies to reduce sample size and stay robust against interactions. The addition of a true control arm representing 0 to 80% of patients was tested, as well as a set of different analysis algorithms adjusting for interactions. While it is generally a bad idea to run factorial trials when interactions are expected [25, 31], and the argument should apply to distributive trials, wrong assumptions on interactions are possible. Thus, some robustness against them can be useful to limit unexpected loss of power, even at some upfront sample size cost. The testing strategies were:

- logistic regression without any interaction term
- logistic regression with a pre-specified interaction term between the two effective interventions
  $${\text{logit}}\left({\text{p}}\left({{\text{Y}}}_{{\text{i}}}=1\right)\right)={\upalpha }+{\upbeta }_{1}{{\text{X}}}_{1,{\text{i}}}+{\upbeta }_{2}{{\text{X}}}_{2,{\text{i}}}+\dots +{\upbeta }_{{\text{K}}}{{\text{X}}}_{{\text{K}},{\text{i}}}+{\upbeta }_{12}{{\text{X}}}_{1,{\text{i}}}{{\text{X}}}_{2,{\text{i}}}$$

(with the same notations as above, except 1 and 2 are the two effective interventions and β_12_ is the additional interaction term)

- logistic regression with the same pre-specified interaction term but only kept in the model if *p* < 0.05
- same as above but with a threshold of *p* < 0.15
- logistic regression with a backward elimination strategy for interaction terms, starting from all interactions involving the tested intervention and removing those with *p* > 0.05
- same as above but with a threshold at *p* > 0.25
- pooled difference, testing the success rate difference in intervention versus non-intervention subjects (and accepting some bias to decrease sample size)

In this scenario group, we held other parameters at constant values (previously explored):

- k and K were either {k, K} = {4,10} or {k, K} = {2,20}
- success rate was 70% for two interventions and 50% (no effect) for others, and the former's combined effectiveness was either 84.48276% (logit-scale additivity) or 70% (lack of additivity).

Other simulation sets are described and presented in Supplementary Materials, investigating FWER and more thoroughly exploring interaction adjustment strategies.

### Software

All computations were made with R version 4.2.2., without parallelization.

## Results

**Fig. 2:**
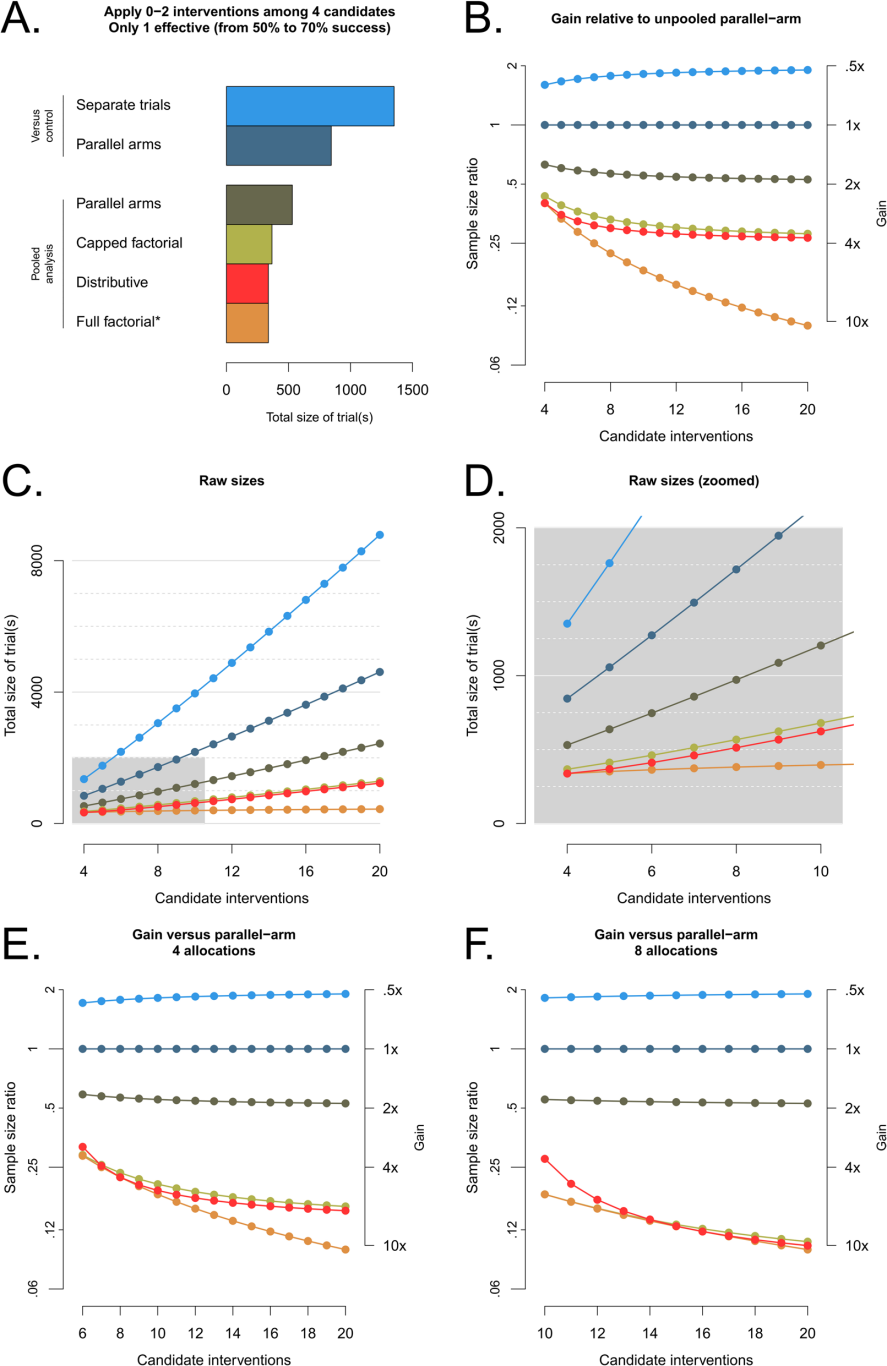
Trial sizes for a single effective intervention among 4 candidates (**A**, with enrolment of separate trials summed) or among 4 to 20 candidates (**C** and **D**, same colors as in **A**) with either 2 (**A**-**D**), 4 (**E**) or 8 (**F**) allocations per patient. **B** shows the total trial size ratio compared to the parallel-arm design (each intervention analyzed versus control) and the associated gains, with the same color scheme as in **A**. **E** and **F** show improvements of the distributive and capped factorial design as more interventions (4 and 8 respectively) are tested per patient (same colors as in **A**). The efficacy assumption is that a single intervention brings the probability of a good outcome from 0.5 to 0.7, and the other interventions have no effect. Alpha risks (nominal 5%) are Bonferroni-adjusted for multiplicity, and 90% power is sought. *the full factorial design is shown for reference but ignores maximum allocations

As shown in Fig. 2 (panels A, B, E and F), when analyzed by testing differences in proportions, distributive trials perform as well as factorial trials when k = K/2 and much better than parallel arm trials. The capped factorial design behaves similarly to the distributive one. The parallel arm design requires 1.5–2× more subjects than the distributive one with k = 2 (the least efficient value for k), when using the most efficient analysis for both (pooling arms in a large control). Increasing the number of per-patient interventions k to 4 and 8 (Fig. 2, panels E and F) allowed further reductions in sample size, similar to that of the full factorial design when K = 2k. For the pooled analyses (capped factorial, pooled parallel, and distributive designs), FWER is controlled if the test is one-sided (Supplementary Fig. S1), but not always if it is two-sided (Supplementary Fig. S2). This is as expected: when testing a null intervention, against a pooled control group with all patients who did not get it, the single effective intervention is more likely to be given to the control group (especially with few candidates), which creates a negative contrast and bias against the null intervention (in the direction of harm) and increases FWER in the two-sided case; conversely, in the one-sided case (testing only for benefit), this bias in the direction of harm reduces FWER.

**Fig. 3:**
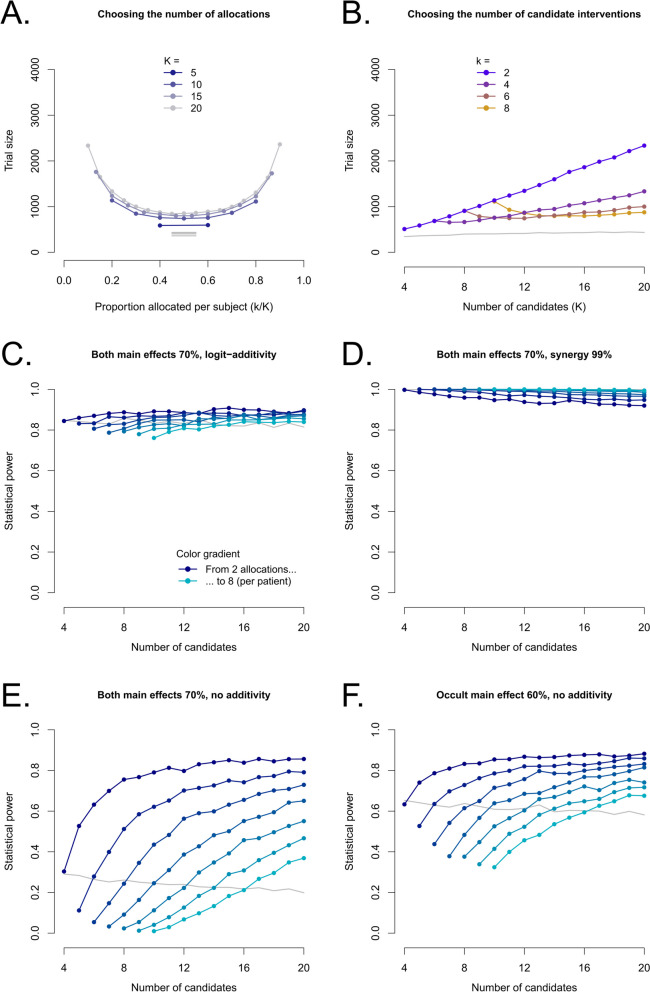
Sample sizes using logistic regression and loss of power with an extra unexpected effective intervention. **A** and **B** show the expected sample size with a single expected effective intervention that increases clinical success from 50 to 70%, based on > 5000 simulations, using logistic regression for analysis and aiming for 90% power. Then, each panel shows a different scenario with an additional unexpected effective intervention with the same main effect size, and, for the combination, either: logit-scale additivity (the combination yielding 84.48276% clinical success) (**C**), strong synergy with 99% clinical success (**D**), or no additivity at all (**E**). In the final case, the unexpectedly effective intervention only has a 60% success rate, and no additivity (**F**). For each panel, the background gray line shows the sample size or true power of a factorial trial powered for the same situation with the same wrong assumptions (changes are mostly due to the multiplicity adjustment). For the distributive designs, each curve is for a different number of allocations per patient k (equal to its starting point minus 2), with one color hue per number of allocations

Still assuming a single effective intervention, but switching the analysis to logistic regression to make it possible to find multiple effective interventions without bias, Fig. 3A shows that the most efficient number of allocations per patient is 50% of candidate interventions, bringing trial sizes close to those of factorial trials (background grey lines for 1:1 factorial design in all panels). Figure 3B shows sample sizes for a given number of allocations, depending on how many interventions are to be tested. The switch to logistic regression, while reasonable if several interventions can be effective, is costly in terms of sample size. The latter is approximately 80% higher compared to a pooled difference of means (Fig. 3B purple line versus Fig. 2C red line), but remains way below those of a parallel trial (from 2× to 5× depending on k and K for K < 20, and even more if K is larger). Incremental increases for additional candidate interventions to be tested are also small compared to an extra arm in a parallel trial, and smaller as k increases (+ 220 subjects for a 20-arm Bonferroni-adjusted parallel trial versus around + 120 with k = 2 and + 20 for k = 8). Sample size increases for additional candidates are very small in particular if the k/K ratio can be maintained (Fig. 3A, distance between curves for 5 extra candidates), much like a factorial trial. As expected thanks to the multiplicity correction, FWER is properly controlled (Supplementary Fig. S3).

The assumption on the number of effective interventions may be false, which will impact power; Fig. 3C-F shows the drop in power when only one intervention was expected to be effective, but another one was also (for a total of two effective interventions). The extent of the decrease depends heavily on the nature of the interaction between the two effective interventions, from small (< 15%) for a logit-scale additive effect (Fig. 3C), to non-existent and even reversed for a synergistic effect (Fig. 3D), to large for a lack of additivity (i.e., a negative interaction) (Fig. 3E), with the effect being somewhat mitigated if there is still some contrast between the interventions and k is small (Fig. 3F). The decrease is modest compared to that observed with a factorial design (background gray lines) if the effective interventions are sufficiently diluted among ineffective ones.

**Fig. 4:**
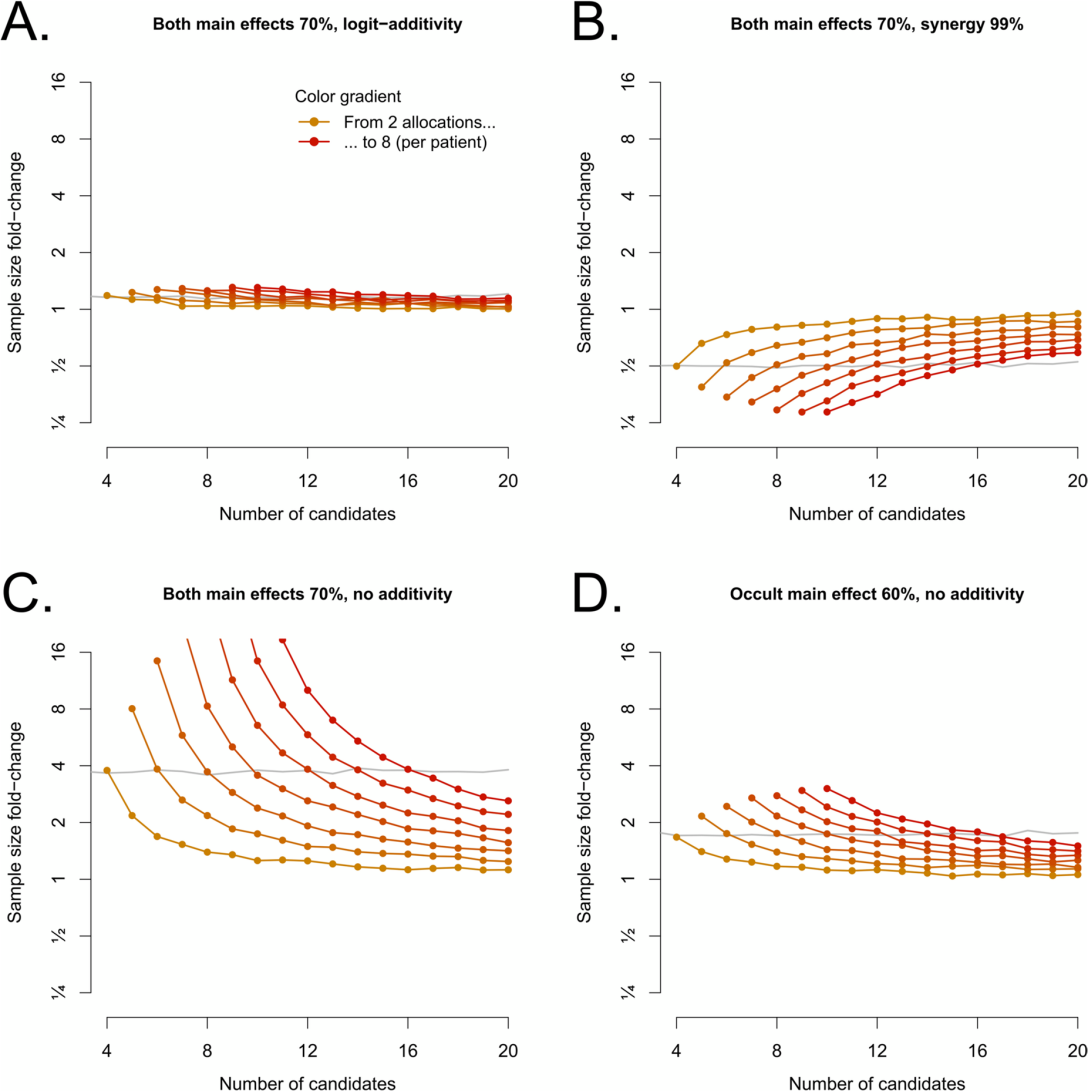
Increase in sample size with an extra expected effective intervention Each panel shows the same scenarios as in Fig. 3, but this time the scenario was correctly assumed to compute sample size

When multiple effective interventions are expected, sample size can be adapted to maintain statistical power. Fig. 4 shows that increases in sample size are modest unless a large fraction of interventions is given to each patient, the effect is non-additive, and the main effects have exactly the same size (Fig. 4C). This special case is difficult, even for factorial trials (Fig. 4C, background gray line). In all 4 scenarios, the multiplicity correction functions as expected and FWER is properly controlled (Supplementary Fig. S4).

While positive interactions increase power and thus do not adversely impact sample size, negative interactions decrease power. Supplementary Figs. S5 and 6 (in Supplementary Materials) show how adding an interaction term in the logistic model can allow it to adjust for the negative interaction and prevent loss of power with an extra effective intervention. They show the same cases as Fig. 3, but with an added interaction term used only for adjustment (conclusions are only drawn about main effects); this term is either systematically used for adjustment (Supplementary Fig. S5), or only kept if *p* < 0.05 (Supplementary Fig. S6). Systematic adjustment provides robustness to wrong assumptions, at limited sample size cost, whereas conditional adjustment is economical in sample size and provides some limited robustness for negative interactions, but can destabilize estimates and decrease power (compared to no adjustment) in the case of positive interactions.

**Fig. 5:**
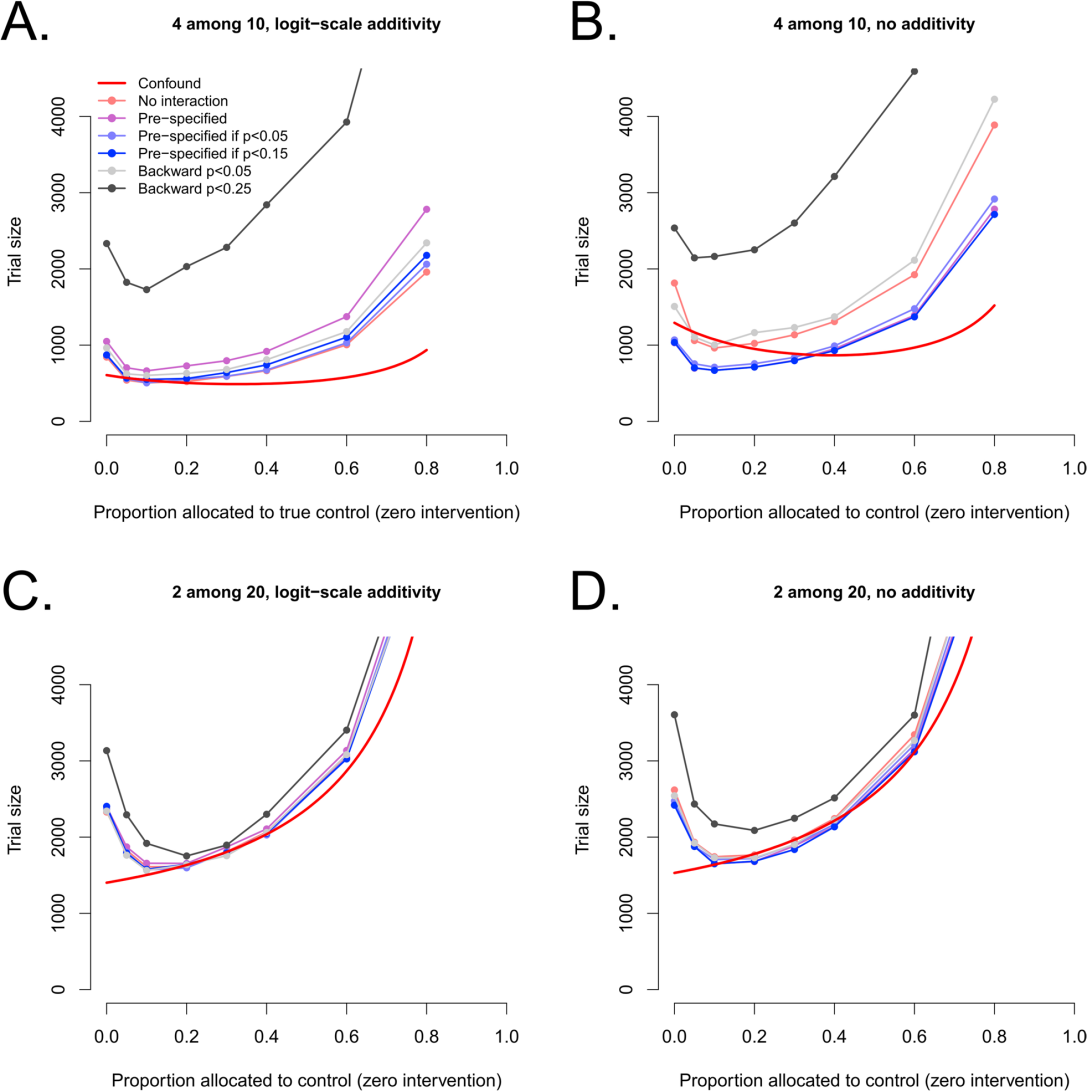
True control arms and mitigation strategies for interactions. **A** shows sample sizes for a scenario with 2 effective interventions (70% success) with additive efficacy on the logit scale (84.48276% clinical success), 8 ineffective interventions (no change), and 4 allocations per patient, as a function of the size of the control arm (relative to total inclusions). **B** shows the same situation but with non-additive efficacy (70% success for the combination, same as each standalone intervention). **C** and **D** show the same two effective interventions (with and without additivity) but with 2 allocations among 20 candidates rather than 4 among 10. The analysis algorithms are: “Confound” for a pooled difference of means between treated and non-treated, accepting bias due to pooling but counting on a small proportion of effective interventions, “No interaction” for a simple logistic regression with the main effects, “Pre-specified” for a single interaction term between the two effective interventions, “Backward” for a backward elimination strategy starting from all interaction terms involving the analyzed intervention and keeping only those with a *p*-value below the threshold

These analysis methods only add one interaction term to adjust for a suspected interaction, but are not useful when interactions are suspected in general but not for some combinations and not others. Some possible strategies for the latter case are shown in Fig. 5. Figure 5A and B show different design and analysis strategies for 4 allocations among 10 candidates, of which 2 are effective either with logit-scale additivity (i.e., no interaction) in Fig. 5A, as in Fig. 3C, or without additivity (i.e., negative interaction) in Fig. 5B, as in Fig. 3E. The allocation of some patients to a true control arm without any active intervention, which corresponds to the controlled distributive design from Fig. 1, can also provide robustness if the effective interventions are sufficiently diluted (as shown by Fig. 5C versus D with 2 allocations among 20 candidates, but less so in 5A versus 5B with 4 allocations among 10 candidates), and also give overall lower sample sizes (-35%).

As previously, a pre-specified interaction term is helpful to adjust for a negative interaction, but requires a good intuition before the trial. Trying to circumvent the need for good intuition by using a backward elimination strategy, starting from all interactions involving an intervention and iteratively eliminating those with *p* > 0.25, also has limited utility, but a more stringent threshold (*p* > 0.05) can work, at some sample size cost. Unsurprisingly, adjusting on all interactions involving the analyzed intervention is not viable because it requires many more subjects (> 9000, not shown on the graph), and the effect is even worse for all two-way interactions and not only those for the analyzed intervention (> 30,000, also not shown). An iterative backward elimination strategy starting from all interactions, is able to prune them and bring sample sizes to reasonable levels, very similar to starting from interactions only with the analyzed intervention (not shown). Fig. 5C and D show sample size determinations for the same true effects but with a strategy involving 2 allocations among 20 candidate interventions; here, interaction terms are not very useful because effective interventions are highly “diluted” among non-effective ones, and interactions rarely occur.

The red lines in Fig. 5 show a strategy where interventions are analyzed by difference of means while simply pooling receiving versus non-receiving subjects, as in Fig. 2, and accepting that the difference will be biased by the mean of the other interventions; the strategy performs surprisingly well in this setting with few effective interventions (2 effective among either 10 or 20), bested only by a combination of a small control arm and a pre-specified interaction term in case of no additivity (Fig. 5B), but its performance would likely quickly drop if many candidate interventions have similar efficacy, and may also run into problems with unbalanced designs [25].

## Discussion

This study evaluates a simple experimental fractionation strategy to evaluate many interventions in parallel, deliberately confounding their effects in some subjects while adjusting the analysis to retrieve correct results. The spirit is similar to that of a factorial trial (the possible combinations per subject are a subset thereof) but mitigates the practical issues with the high number of per-patient allocations in factorial trials beyond the 2 × 2 case, while still retaining large decreases in sample size compared to parallel trials. It has the added benefit of providing (multiple) active interventions to all subjects, which could increase participation rates by as much as 20–50 percentage points in clinical trials given patients’ distaste for placebos [32, 33].

Many biomedical fields could benefit from distributive randomization, and there are several examples of recent trials that could have used it. During the early COVID pandemic, several in vitro screening experiments produced many candidates for drug repositioning, many of which did not appear to be effective with further research [34]. Trials such as DisCoVeRy [35], RECOVERY [36] or REMAP-CAP [37] could have produced faster results or evaluated more candidates with such an approach. Our experience with DisCoVeRy also showed high rates of participant refusal due to the control arm. In another setting, the NUDGE-FLU trial recently evaluated 9 different electronic letters to encourage influenza vaccine uptake in the general population [38], using a parallel-arm design, and could have similarly benefitted from multiple interventions per subject; only 2 out of 9 arms showed a (small) effect, and the planned instrumental variable analysis could have benefitted from more interventions given to each subject, not to mention the potential benefit for subjects themselves. Future similar prevention trials could benefit from distributive randomization. Nutrition is another field where many possible interventions can be tested and where the scattershot approach of traditional trials has difficulty producing results, due to unclear rationales and probably small effect sizes [39]; large distributive randomization trials testing 20 or 30 nutrients in a few thousand patients could be of great help there, especially if linked with administrative databases for simpler follow-up. Finally, another area of interest is open online trials, which are starting to be a modality [40] and for which sample sizes are constrained and subject motivation is critical, both problems being alleviated by this design.

When planning a distributive trial, assumptions must be carefully laid out. In particular, one should avoid excessive optimism about the effectiveness of candidate interventions, as the history of clinical trials warrants. Even thoroughly vetted drug candidates in phase III fail due to a lack of efficacy approximately half the time [41]. This is even more common with supplements [42, 43]. Behavioral interventions, even without a clinical endpoint, also have a high failure rate [44]. Unless a true control arm is added, distributive randomization actively relies on some interventions not working, and we fear that this could be a barrier to adoption. One way to manage this objection is simply to evaluate additional candidate interventions that are unlikely to work, because the incremental sample size increases are small. Another possible objection is the correction for multiplicity; some authors argue this adjustment may not be necessary in all settings [45, 46], while other researchers [23] and regulators [24] are less liberal. In this study, we applied the most conservative strategy and used it systematically, to control the overall alpha risk of introducing a useless intervention at the whole clinical trial level. Given that one strength of the distributive design is that additional candidates have small extra sample size cost, clinicians may want to investigate candidates with weaker presumptions of efficacy, so adjusting for multiplicity seems more prudent. As shown in Supplementary Figs. 1– 4, this adjustment is sufficient to control for family-wise-error rates (provided the underlying testing procedure makes sense).

The distributive design has drawbacks. In settings where giving a high proportion of candidate interventions to each patient is possible, it will underperform full factorial and classically aliased fractional factorial trials (of note, those have the same sample size requirements in our scenarios, as seen in Supplementary Fig. S7). If only some combinations are of clinical concern, a classical fractionation will be better, as it can weed those out of the design matrix, while requiring significantly fewer resources to run than the distributive design. Another drawback of distributive randomization is that like in full factorial trials, the mixing of interventions comes at the expense of some estimand clarity, because the effect of interventions is measured in a population receiving a mix of other interventions (most of which are supposed and/or tested, correctly or incorrectly, to be ineffective and/or non-interacting). Furthermore, its planning has many moving parts, some of which are introduced here (the k/K ratio, the existence and size of the control arm, the inclusion of interaction terms), and some of which remain to be addressed in the future or for particular cases (unbalanced allocations, further fractionation of the distributive design with forbidden or enriched combinations, dose levels).

Because of these, one should run careful simulations when planning such a trial, and try to make sure the design is robust if the interventions do not work as expected. As a starting strategy, we recommend either avoiding or grouping (as levels of a single factor) interventions with suspected non-additive effects due to e.g. the same mechanism of action (or, if impractical, adjusting with a few dedicated pre-specified interaction terms, at the cost of additional inclusions), increasing allocations per patient (k/K) if possible (no further than 40–50%, and less than that if negative interactions are suspected and cannot be adjusted), and admitting potentially ineffective candidate interventions in the trial liberally. Adjusting for interactions is generally not recommended in classical factorial 2 × 2 trials, because half of the information is spent on estimating the interaction term, making the design useless with respect to sample size if the goal is to estimate main effects (this can be seen in the factorial design sample size increase between Fig. 3A and Supplementary Fig. 5A). But the effect is much less pronounced with distributive trials (same figures, same panels). This is probably because these interactions happen more rarely in the latter; the same attenuation of this effect can be observed with back-of-the-envelope simulations of unbalanced factorial allocations (not shown). Because of this dilution, even outright ignoring interactions and adjustments becomes possible if there are few effective interventions among many exploratory ones. A small true control arm (5–20% of subjects), while it introduces inequality between subjects and may be hard to justify to them and to investigators, can reduce sample size significantly; its presence should be debated case by case, as a tradeoff between cost and length on one hand, and fairness and absence of self-selection of subjects on the other. All scripts used for this study are provided, and the authors can be contacted to replicate or adapt the computations for a planned trial.

## Conclusion

Distributive trials are a simple but efficient fractionated experimental design that can help expedite evaluation in under-evidenced areas of medicine, such as emerging diseases, supplements, nutrition, probiotics, behavioral or lifestyle modifications, and digital interventions. Their strengths include more fairness to participants, small sample sizes, and low sensitivity to additional interventions with unlikely efficacy, while its adjustable parameters require foresight and simulation work from investigators. The aforementioned strengths are especially well-suited to a rapidly evolving healthcare landscape where therapeutic options are ever more numerous, digital tools are gaining in strength and reach and enabling larger sample sizes, patients are increasingly willing to challenge expertise and try things on their own, and the distinction between wellness and medicine can become blurry.

In this changing context, the clinical research community should ensure that the evidence-based spirit of modern medicine stays in step with patients and society, and is not confined to academic and industrial settings. Instead of often simply shrugging and citing lack of evidence, we can use innovative and efficient trial designs to produce it. Creativity in this area can help unlock the potential of not only new inventions, but also nonpatentable or otherwise overlooked strategies that may have been available for years or even centuries, and about which evidence-based medicine’s lack of real answers is often not accepted by segments of broader society.

### Supplementary Information

**Supplementary Material 1.**

## Data Availability

The exact code used to generate the simulations and the figures is provided in the Supplementary Appendix.
