# Supplementary material for: Distributive randomization: a pragmatic fractional factorial design to screen or evaluate multiple simultaneous interventions in a clinical trial
Source: BMC Med Res Methodol. 2024 Mar 11;24:64. doi: 10.1186/s12874-024-02191-9 (PMC11340141; doi:10.1186/s12874-024-02191-9)
Supplement: Supplementary file 1 — Supplementary Material 1. [file 12874_2024_2191_MOESM1_ESM.docx]

# Title

Distributive randomization: a pragmatic fractional factorial design to screen or evaluate multiple simultaneous interventions in a clinical trial - **Supplementary Materials**

# Authors and affiliations

1. Skerdi Haviari^1,2^, France Mentré^1,2^

^1^ Département Epidémiologie Biostatistiques et Recherche Clinique, AP-HP, Hôpital Bichat, Paris, France.

^2^ Université Paris Cité UMR1137 (IAME), INSERM, Paris, France.

# Supplementary Materials Contents

[Title 1](#_Toc158192487)

[Authors and affiliations 1](#_Toc158192488)

[Supplementary Materials Contents 1](#_Toc158192489)

[Supplementary methods 2](#_Toc158192490)

[Additional simulations 2](#_Toc158192491)

[Combinatorics 3](#_Toc158192492)

[Effect specification 4](#_Toc158192493)

[Fractional versus full factorial comparison 5](#_Toc158192494)

[Supplementary Figures 6](#_Toc158192495)

[Supplementary Figure S1 7](#_Toc158192496)

[Supplementary Figure S2 8](#_Toc158192497)

[Supplementary Figure S3 9](#_Toc158192498)

[Supplementary Figure S4 10](#_Toc158192499)

[Supplementary Figure S5 11](#_Toc158192500)

[Supplementary Figure S6 12](#_Toc158192501)

[Supplementary Figure S7 13](#_Toc158192502)

[#Setup code 14](#_Toc158192503)

[#Utilities 14](#_Toc158192504)

[#Combinatorics for allocation table 15](#_Toc158192505)

[#Sample size and power for difference of proportions 16](#_Toc158192506)

[#Logistic regression algorithms (different strategies) 17](#_Toc158192507)

[#Power by simulation 19](#_Toc158192508)

[#Sample size by simulation 21](#_Toc158192509)

[#Scripts to reproduce the main figures 23](#_Toc158192510)

[#Compute and draw Figure 2 23](#_Toc158192511)

[#Generate data for Figures 3 and 4 26](#_Toc158192512)

[#Draw Figures 3 and 4 28](#_Toc158192513)

[#Generate data for Figure 5 30](#_Toc158192514)

[#Draw Figure 5 31](#_Toc158192515)

[#Scripts to reproduce the supplementary figures 32](#_Toc158192516)

[#Simulate and draw Supplementary Figure S1 32](#_Toc158192517)

[#Simulate and draw Supplementary Figure S2 36](#_Toc158192518)

[#Generate data for Supplementary Figure S3 40](#_Toc158192519)

[#Generate data for Supplementary Figure S4 42](#_Toc158192520)

[#Draw Supplementary Figures S3 and S4 43](#_Toc158192521)

[#Generate data for Supplementary Figure S5 44](#_Toc158192522)

[#Draw Supplementary Figure S5 46](#_Toc158192523)

[#Generate data for Supplementary Figure S6 48](#_Toc158192524)

[#Draw Supplementary Figure S6 50](#_Toc158192525)

[#Generate data for Supplementary Figure S7 52](#_Toc158192526)

[#Draw Supplementary Figure S7 55](#_Toc158192527)

[#Code for quick calculations used in reviewer response 1 56](#_Toc158192528)

# Supplementary methods

## Additional simulations

Additional simulation were run to check values and explore additional scenarios.

Scenario group 1 from the main article was evaluated using simulations rather than analytical forms, checking power and family-wise error rate (FWER, computed among interventions with no effect). 1000 simulations per point were run. Error bars are based on a Jeffreys interval for proportions.

Scenario group S1 is the same as Scenario group 1 but the statistical test is two-tailed rather than one-tailed. This is not appropriate since, in the presence of an effective intervention, the pooled difference of proportions introduces biases in the direction of harm when testing any of the null interventions (that intervention takes a "slot" and thus the effective intervention is more likely in the control group).

Scenario groups 2 and 3 are as in the main article, FWER was also computed for 1000 simulations per point.

Scenario groups S2 and S3 are as figure 3 in the main article exploring the distributive design, but the testing strategy is adjusted on interaction terms. For S2

| Scenario group | Designs | k | K | Interventions' probability of success | | | Analysis | Foresight /Estimand | Figure |
| --- | --- | --- | --- | --- | --- | --- | --- | --- | --- |
|  |  |  |  | 1 | 2 | 1+2 |  |  |  |
| 1 | All | 2 | 4-20 | 70% | 50% | 70% | Pooled one-sided difference of proportions | Yes/Power | S1A |
|  |  |  |  |  |  |  |  | Yes/FWER | S1B |
|  |  | 4 |  |  |  |  |  | Yes/Power | S1C |
|  |  |  |  |  |  |  |  | Yes/FWER | S1D |
|  |  | 8 |  |  |  |  |  | Yes/Power | S1E |
|  |  |  |  |  |  |  |  | Yes/FWER | S1F |
| S1 | All | 2 | 4-20 | 70% | 50% | 70% | Pooled **two-sided** difference of proportions | Yes/Power | S2A |
|  |  |  |  |  |  |  |  | Yes/FWER | S2B |
|  |  | 4 |  |  |  |  |  | Yes/Power | S2C |
|  |  |  |  |  |  |  |  | Yes/FWER | S2D |
|  |  | 8 |  |  |  |  |  | Yes/Power | S2E |
|  |  |  |  |  |  |  |  | Yes/FWER | S2F |
| 2 | Distributive | 2-8 | 4-20 | 70% | 70% | 84.5%* | Logistic regression without interaction | Yes/FWER | S3A |
|  |  |  |  |  |  | 99% |  |  | S3B |
|  |  |  |  |  |  | 70% |  |  | S3C |
|  |  |  |  |  | 60% | 70% |  |  | S3D |
| 3 | Distributive | 2-8 | 4-20 | 70% | 70% | 84.5%* | Logistic regression without interaction | **No**/FWER | S4A |
|  |  |  |  |  |  | 99% |  |  | S4B |
|  |  |  |  |  |  | 70% |  |  | S4C |
|  |  |  |  |  | 60% | 70% |  |  | S4D |
| S2 | Distributive, factorial | 3-8 | 5, 10, 15, 20 | 70% | 50% | 50% | Logistic regression with pre-specified interaction | Yes /Sample size | S5A |
|  |  | 2, 4, 6, 8 | 4-20 |  |  |  |  |  | S5B |
|  |  | 2-8 |  |  | 70% | 84.5%* |  | No /Power | S5C |
|  |  |  |  |  |  | 99% |  |  | S5D |
|  |  |  |  |  |  | 70% |  |  | S5E |
|  |  |  |  |  | 60% | 70% |  |  | S5F |
| S3 | Distributive, factorial | 3-8 | 5, 10, 15, 20 | 70% | 50% | 50% | Logistic regression with pre-specified interaction gated at p<0.05 | Yes /Sample size | S6A |
|  |  | 2, 4, 6, 8 | 4-20 |  |  |  |  |  | S6B |
|  |  | 2-8 |  |  | 70% | 84.5%* |  | No /Power | S6C |
|  |  |  |  |  |  | 99% |  |  | S6D |
|  |  |  |  |  |  | 70% |  |  | S6E |
|  |  |  |  |  | 60% | 70% |  |  | S6F |

**Supplementary Table S1 Additional simulation and analysis scenarios**

Notations are as in the main article. FWER Family-Wise Error Rate, computed among interventions with no effect. Scenario groups 1-2 are as in the main paper, scenarios S1 to S3 are additional.

## Combinatorics

In the provided code, the combinatorics algorithm is a bit more complex than the case described in the main article. Once L interesting and K-L uninteresting interventions have been defined, rather than use a simple binomial distribution to compute the probability of getting exactly the correct number of allocations among the remaining uninteresting interventions, the code outright multiplies the allocation probability of the K-L remaining treatments (allowing them to have different allocation ratios). The explanation below uses the same notation as the main text methods : p''(X_I_) is the probability of getting the right number of uninteresting interventions.

In the provided code, all possible values of p''(X_I_) (which depends on the number of draws among the L interesting interventions, which will be called l = $\sum_{i=1}^{L} X_{i}$, and the values of p_i_ for i=L+1 to K) are pre-computed using a Markov chain random variable, Q, which can take values from 0 to k+1, representing the number of allocated interventions drawn sequentially, with the value k+1 value representing too many allocations (without detailing how many).

Formally, the idea is to create a 3D-tensor, M, with element M_i,j,l_ representing the probability of having Q = j after i draws starting from l allocated interventions among the L interesting ones, we initiate M with $M_{i,j,l}=0$ for i ≤ L, except for :

- i = L and j = l, l ≤ k, $M_{i,j,l}=1$ (initiation of the Markov variable at l allocated interventions after the first L draws)
- i = L and j = k+1, and all l > k $M_{i,j,l}=1$ (if too many draws among the first L, the Markov variable is initiated at the excess interventions state and will stay there)

M is then updated for each successive possible draw (by incrementing i), using :

- for j < k+1 $M_{i,j,l}={p_{i}M}_{i-1,j-1,l}+ \left( 1-p_{i} \right)M_{i-1,j,l}$ (stochastic increase of Q)
- for j = k+1 $M_{i,j,l}={p_{i}M}_{i-1,j-1,l}+ M_{i-1,j,l}$ (Q cannot leave the excess interventions state)

Once M is pre-computed, p''(X_I_) for the distributive design equals M_K,k,l_ for l = $\sum_{i=1}^{L} X_{i}$, which is the probability to have exactly k final allocations, starting from l among L, after K interventions have had their attempted draw.

For the capped factorial design, p''(X_I_) = $\sum_{j=1}^{k} M_{K,j,l}$ since fewer than k allocations are also allowed.

For the full factorial design, p''(X_I_) = 1 since any number of allocations is allowed.

The final allocation probability is then

p(X_I_) = $\frac{p'(X_{I})p''(X_{I})}{\sum p'(X_{I})p''(X_{I})}$

In the provided implementation the first dimension of M (indexed by i above) is not explicitly kept since only its last value is relevant to the rest of the computations. The code is not able to compute a non-zero effect for uninteresting interventions, but could be adapted relatively easily to do so if implemented with an explicitly declared M defined as above (since the computation will keep track of allocation probabilities by exact number of uninteresting interventions), and a rule to combine not explicitly defined interventions' effectiveness.

## Effect specification

In the provided implementation, effects are inputs that need to be very explicit, in the following format :

- Baseline success rate is specified with c("--"=0.5) (for example).
- Each treatment has a number (from 1 to K), and names of the form "-1-" are used for them.
- Combined effect is supplied with elements named e.g. "-1-2-3-" which provide a clinical success probability for patients receiving all three interventions.
- Higher-order specifications supersede lower-order ones e.g. "-1-2-"=0.8 supersedes "-1-"=0.7 for patients getting interventions 1 and 2.
- Conflicting specifications of the same order, e.g. c("-1-2-"=0.8,"-1-3-"=0.7,""=0.5) for patients getting interventions 1, 2 and 3, are set by the parameter alphabetically ranked last. This was not tested thoroughly so it is advised to specify terms to avoid them e.g. in this example specify "-1-2-3-"=0.8.
- If a patient or arm has none of the specified interventions, the baseline probability is used.
- As follows from the previous rules, by default, no additivity is implemented e.g. c("-1-"=0.7,"-2-"=0.7,""=0.5) gives a 0.7 success probability for patients getting interventions 1 and 2. Situations with many effective interventions with additive effects will require automated generation of combined effect terms if the code below is used.

## Fractional versus full factorial comparison

In order to explore the impact of fractionation, a standard fractional design, with 40% of interventions aliased (rounded down), was compared to a full factorial design in terms of sample sizes. Fractional design matrices were obtained using R package FrF2 (Grömping, U. (2014). R Package FrF2 for Creating and Analyzing Fractional Factorial 2-Level Designs. *Journal of Statistical Software*, *56*(1), 1–56. https://doi.org/10.18637/jss.v056.i01). Results are presented in Supplementary Figure S7.

# Supplementary Figures

##
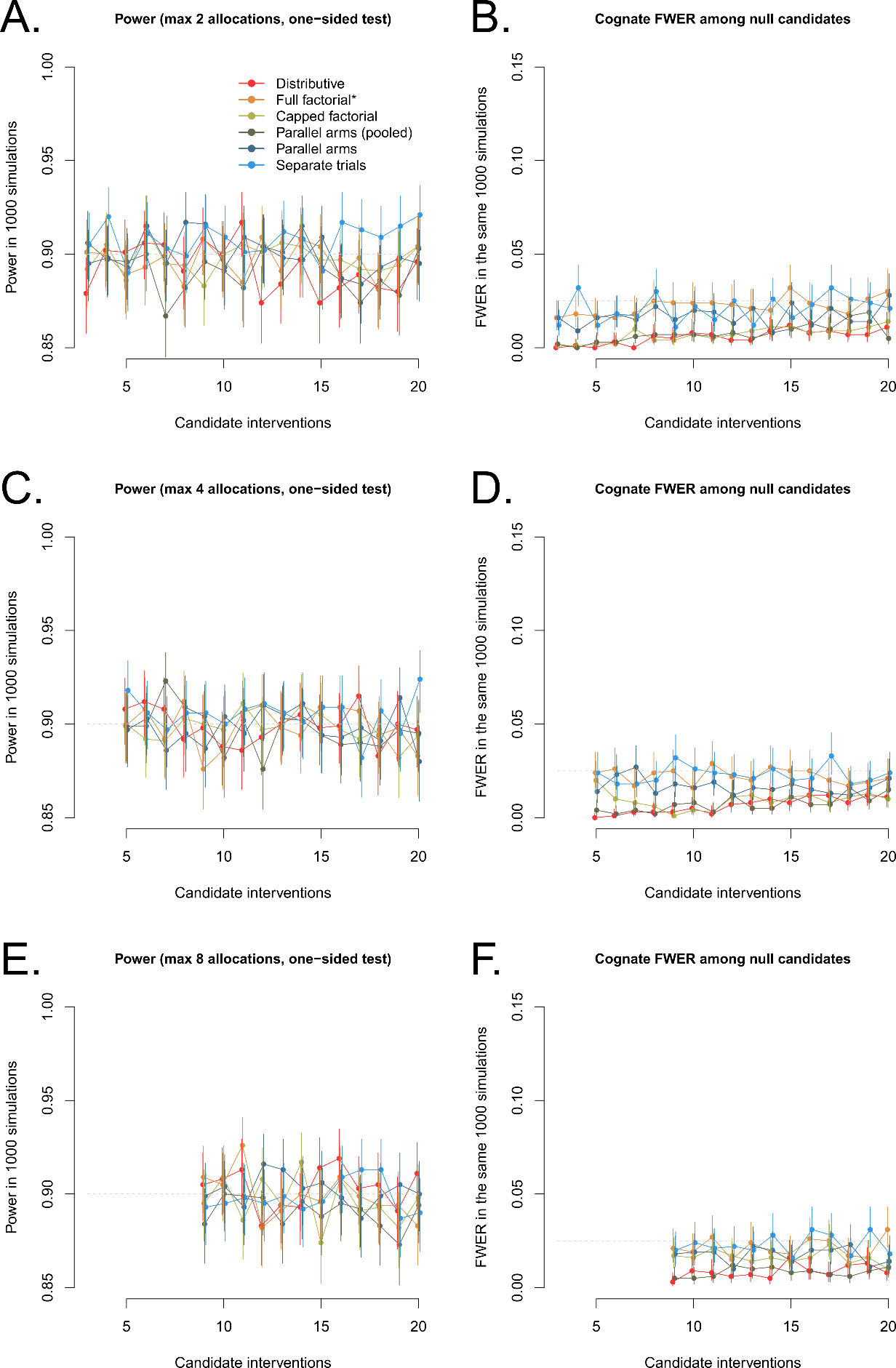
Supplementary Figure S1

**Supplementary Figure S1** **Power and FWER with a single effective intervention tested by one-sided difference of proportions** between patients receiving and not receiving each intervention. Assumptions are those of Figure 2 in the main article. Panels ACE for power, BDF for FWER, AB for 2 allocations, CD for 4 allocations, EF for 8 allocations. Error bars are Jeffreys credibility intervals for Monte Carlo variability. FWER : Family Wise Error Rate.

##
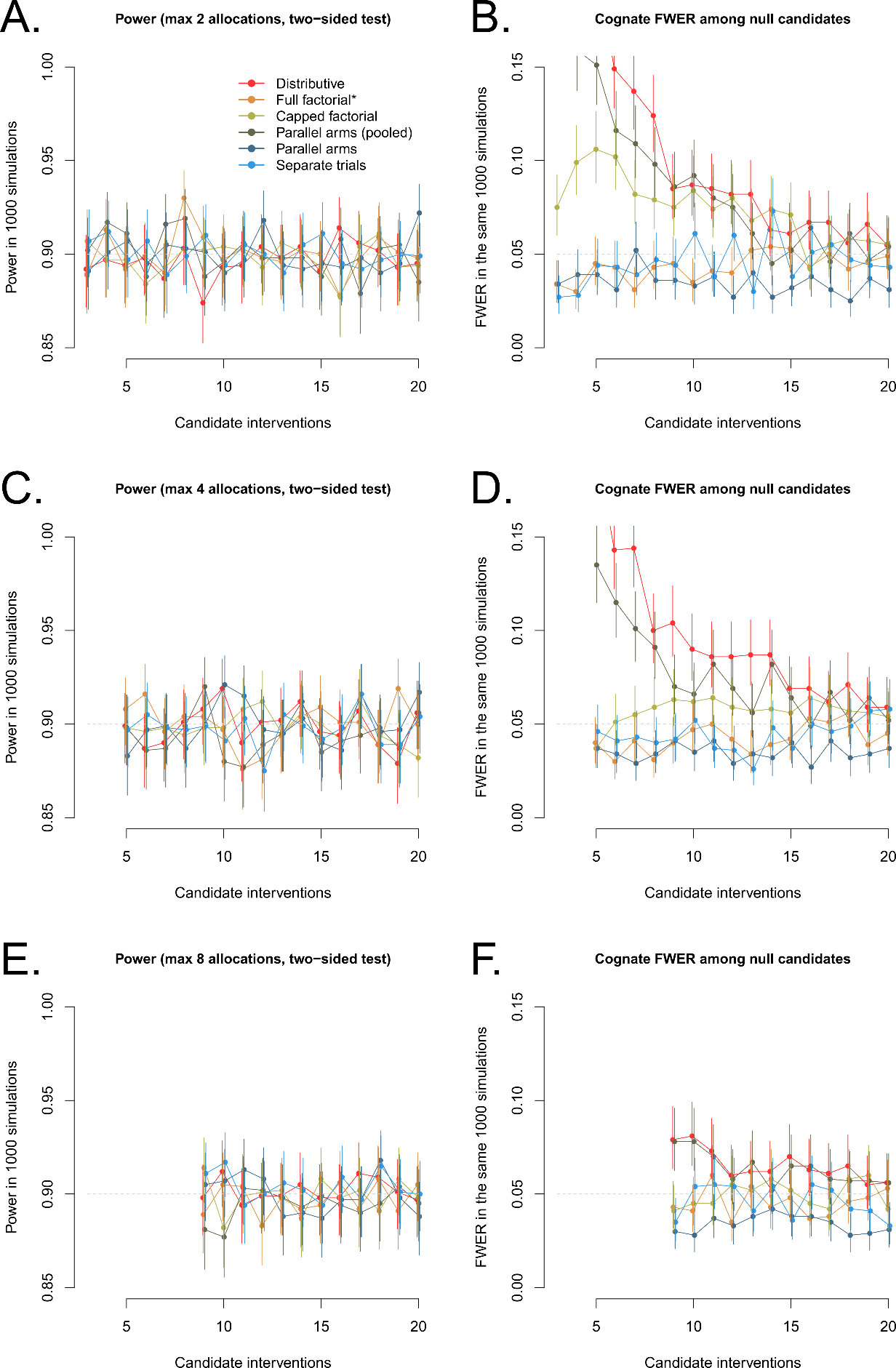
Supplementary Figure S2

**Supplementary Figure S2 Power and FWER with a single effective intervention tested by two-sided difference of proportions**, otherwise as in Supplementary Figure S1.

##
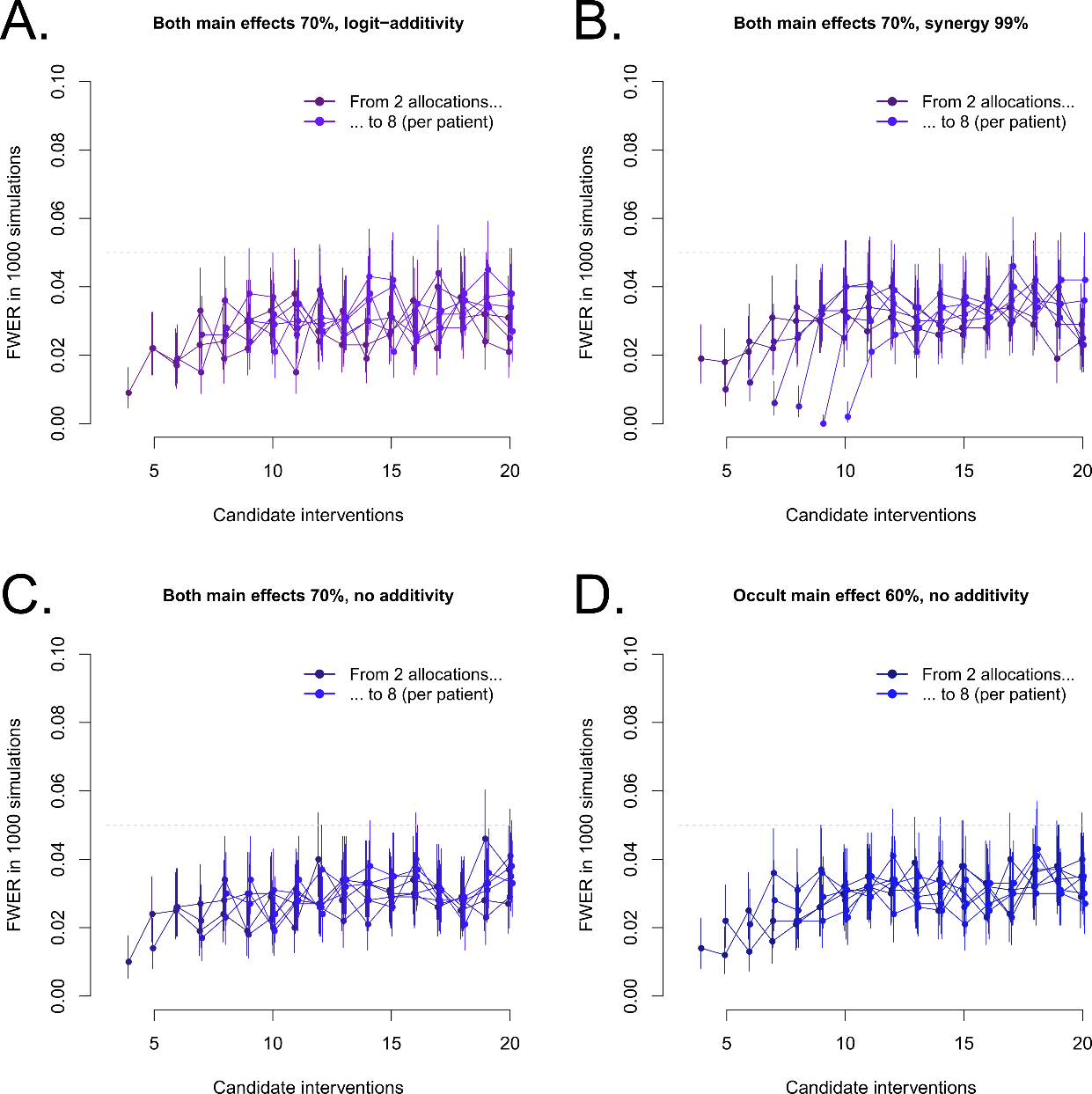
Supplementary Figure S3

**Supplementary Figure S3 FWER with testing done by a logistic model with main effects only.** Scenarios are as in Figure 4 in the main article, with correct assumptions for sample size calculations (those sample sizes are used in the simulations). Error bars are Jeffreys credibility intervals for Monte Carlo variability. FWER : Family Wise Error Rate

##
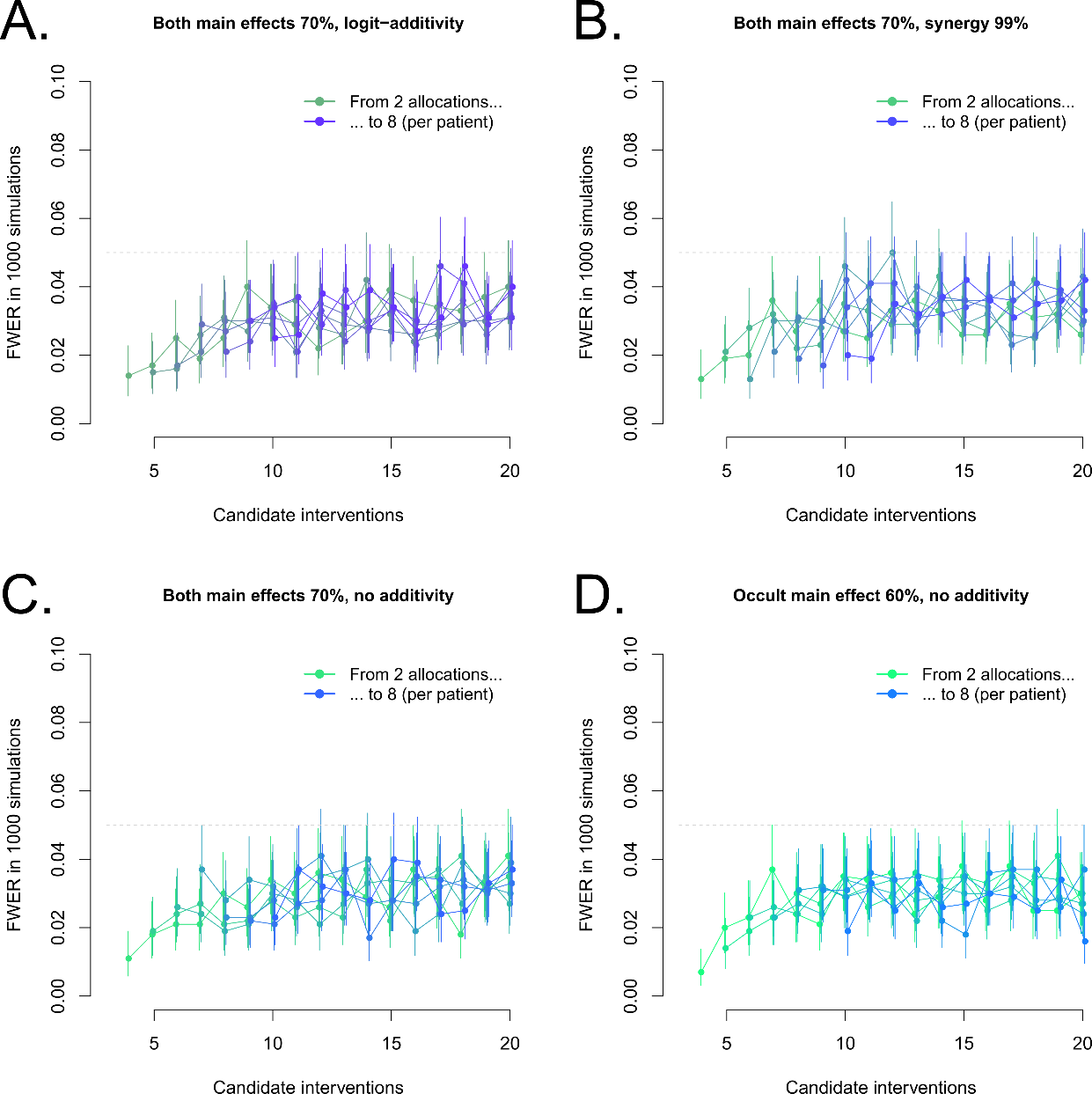
Supplementary Figure S4

**Supplementary Figure S4 FWER with testing done by a logistic model with main effects only.** Scenarios are as in Figure 3C-F in the main article, with an incorrect assumption for sample size calculations (that of a single effective intervention), and therefore an incorrect sample size for the different simulation truths. Error bars are Jeffreys credibility intervals for Monte Carlo variability. FWER : Family Wise Error Rate

##
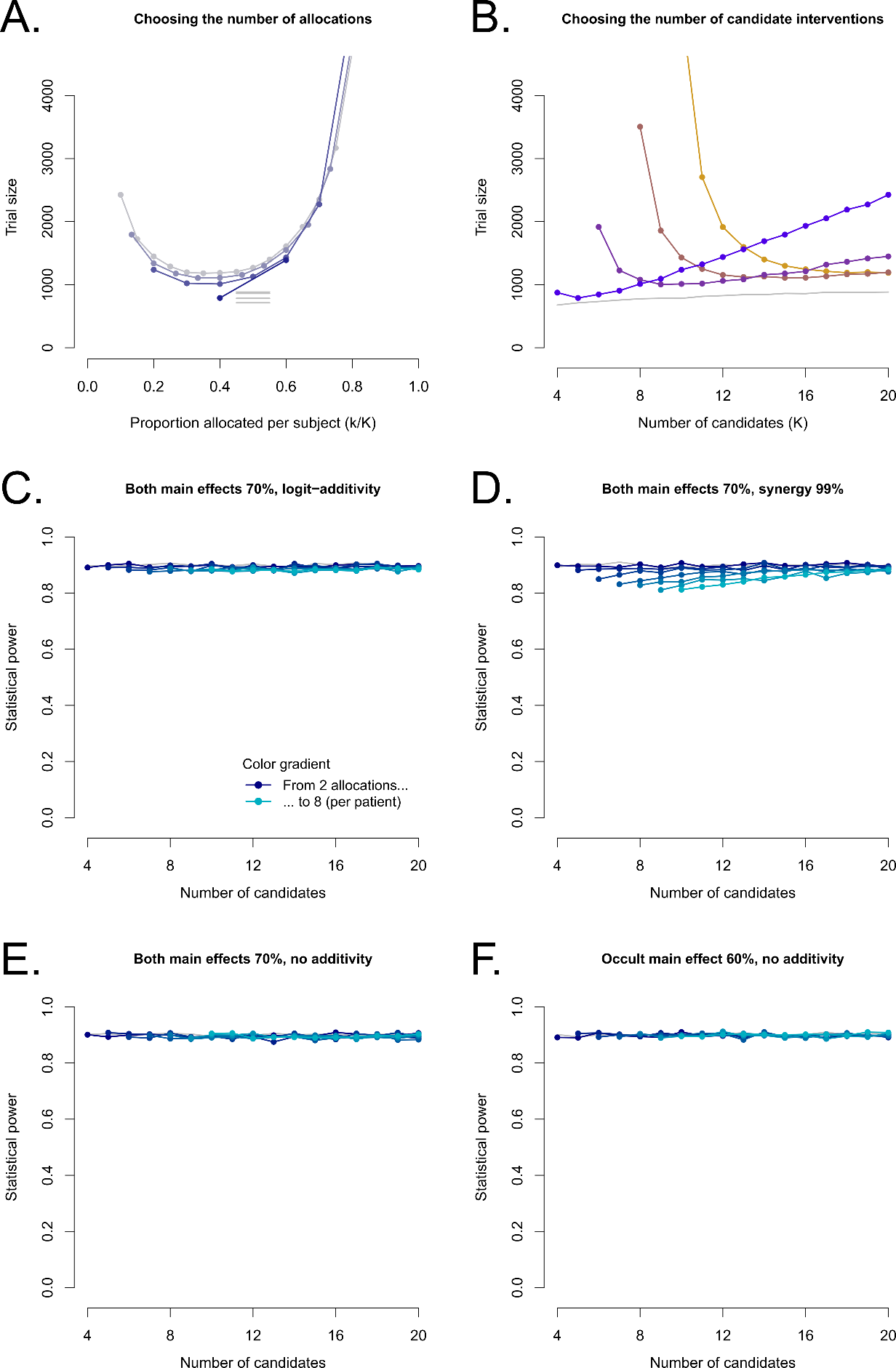
Supplementary Figure S5

**Supplementary Figure S5** **Sample sizes and power for a logistic model testing main effects while systematically adjusting for a suspected interaction between two candidates.** Otherwise parameters are as in Figure 3 of the main article.

##
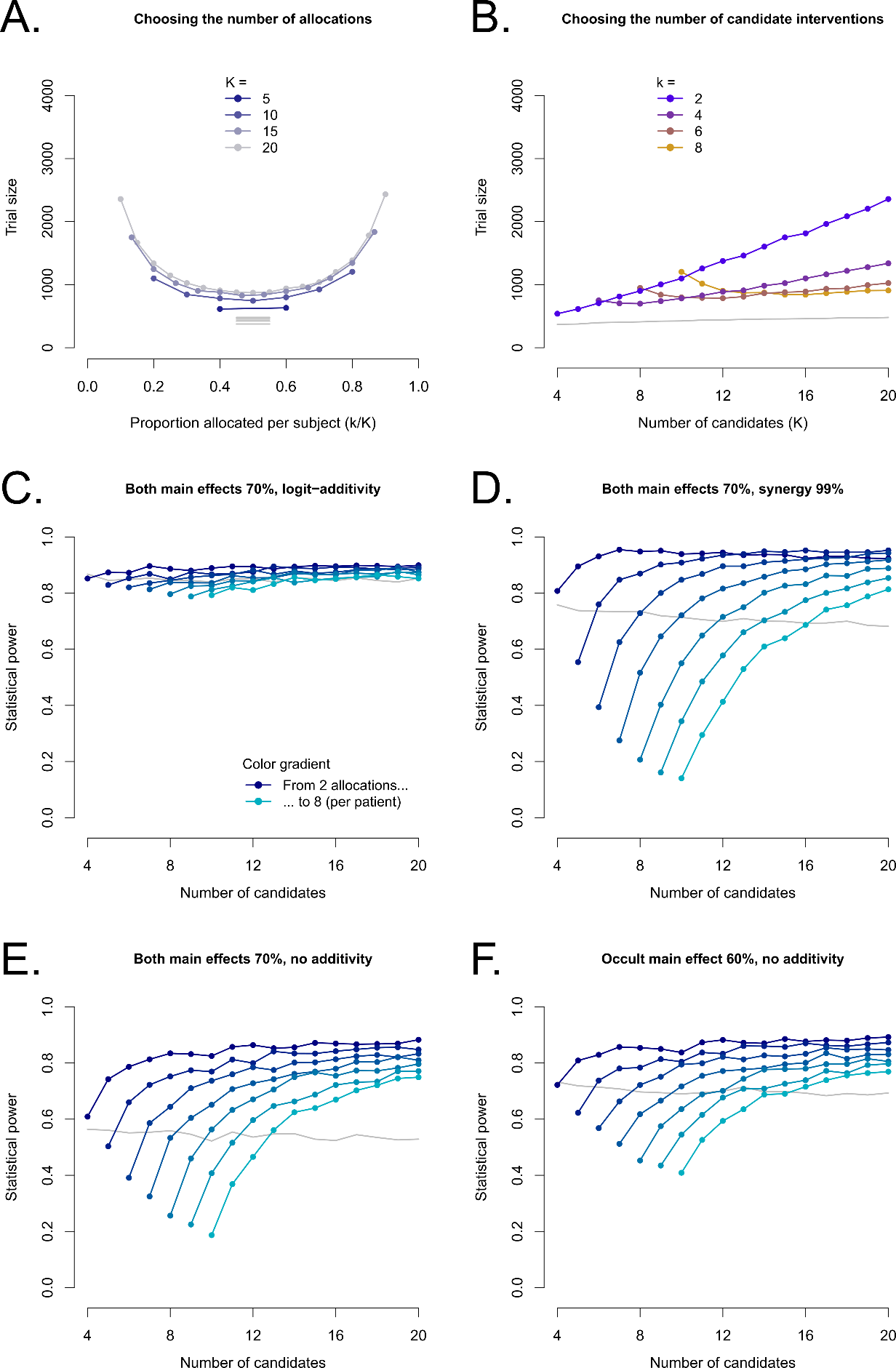
Supplementary Figure S6

**Supplementary Figure S6** **Sample sizes and power for a logistic model testing main effects while adjusting for a suspected interaction between two candidates only if the interaction term has p<0.05.** Otherwise parameters are as in Figure 3 of the main article and Supplementary Figure S5 above.

##
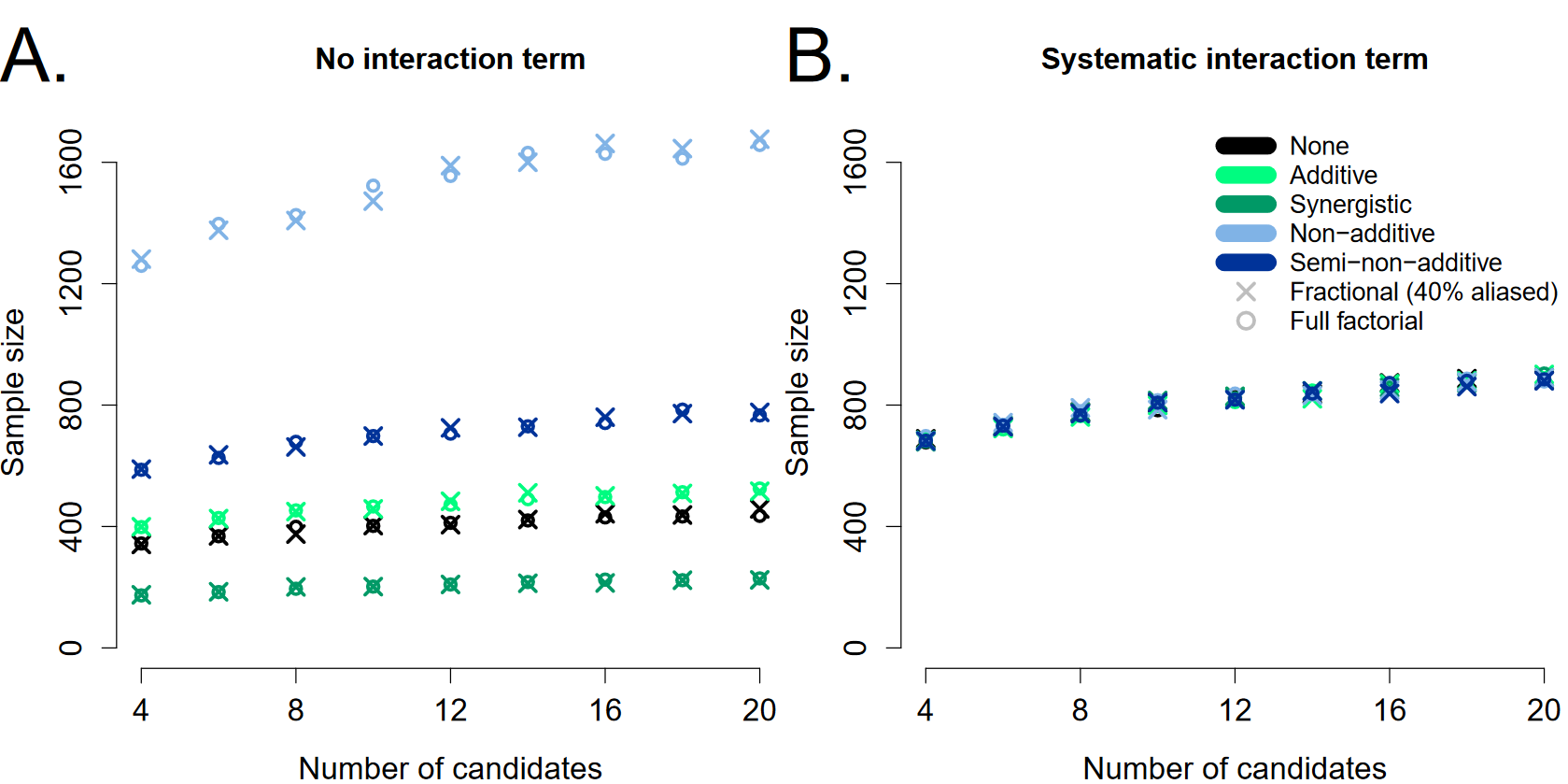
Supplementary Figure S7

**Supplementary Figure S7 Fractional factorial versus full factorial sample sizes** Fractional design matrices have 40% (rounded down) of interventions aliased with high-order interactions. Crosses are for fractional, rounds are for full, colors are as indicated. Panel A for logistic models without interaction terms, panel B for models with the relevant interaction term.

# #Setup code

#The # mark (comment in R code) is used in titles so that the code can be copy-pasted in an editor or terminal and run directly.

#The functions in this section are called by the other scripts. For ease of use, no packages are necessary except for the last supplementary figure.

#(if viewing the original file,"Web mode" visualization is recommended for this section)

## #Utilities

topdf = TRUE # graphic output to pdfs ; there will be bugs if trying to run all scripts in one shot in window displays (size issues)

expit = function(xx){1/(1+exp(-xx))}

bsamsize <- function(p1, p2, fraction=.5, alpha=.05, power=.8) # from https://rdrr.io/cran/Hmisc/src/R/bpower.s

{

z.alpha <- qnorm(1-alpha/2)

z.beta <- qnorm(power)

ratio <- (1-fraction)/fraction

p <- fraction*p1+(1-fraction)*p2

n1 <- (z.alpha * sqrt((ratio+1) * p * (1-p)) +

z.beta * sqrt(ratio * p1 * (1-p1) + p2 * (1 - p2))

)^2/ratio/((p1-p2)^2)

n2 <- ratio*n1

n1+n2 #c(n1=n1, n2=n2) modified from the original

}

# based on

# Fleiss JL, Tytun A, Ury HK (1980): A simple approximation for calculating sample sizes for comparing independent proportions. Biometrics 36:343--6.

# Brittain E, Schlesselman JJ (1982): Optimal allocation for the comparison of proportions. Biometrics 38:1003--9.

# Gordon I, Watson R (1996): The myth of continuity-corrected sample size formulae. Biometrics 52:71--6.

bpower <- function(p1, p2, fraction1, nn, alpha=.05) # adapted from https://rdrr.io/cran/Hmisc/src/R/bpower.s

{

n1 = round(fraction1*nn)

n2 = nn-n1

z <- qnorm(1-alpha/2)

q1 <- 1-p1

q2 <- 1-p2

pm <- (n1*p1+n2*p2)/(n1+n2)

ds <- z*sqrt((1/n1 + 1/n2)*pm*(1-pm))

ex <- abs(p1-p2)

sd <- sqrt(p1*q1/n1+p2*q2/n2)

1-pnorm((ds-ex)/sd)+pnorm((-ds-ex)/sd)

}

# based on

# Fleiss JL, Tytun A, Ury HK (1980): A simple approximation for calculating sample sizes for comparing independent proportions. Biometrics 36:343--6.

# Brittain E, Schlesselman JJ (1982): Optimal allocation for the comparison of proportions. Biometrics 38:1003--9.

# Gordon I, Watson R (1996): The myth of continuity-corrected sample size formulae. Biometrics 52:71--6.

palette_anchors = rbind(c(0.0,1.0,0.2,0.2),c(0.33,0.8,0.8,0.3),c(0.67,0.3,0.3,0.3),c(1,0.2,0.6,0.9))

color_spread = function(nn,pp)

{ cbind(

pp[1,-1],

sapply((1:(nn-1))/(nn-1),function(xx){ above=which(xx<=pp[,1])[1] ; below=above-1 ; pp[below,2:4]+(pp[above,2:4]-pp[below,2:4])*(xx-pp[below,1])/(pp[above,1]-pp[below,1]) })

) }

cc = apply(color_spread(6,palette_anchors),2,function(xx){rgb(xx[1],xx[2],xx[3])})

names(cc) = c("Distributive","Full factorial*","Capped factorial","Parallel arms (pooled)","Parallel arms","Separate trials")

## #Combinatorics for allocation table

focused_allocations = function(odds=rep(1,10), allocs = 4, true_effects = c("-1-2-3-"=0.9,"-1-"=0.7,"--"=0.5), modality=c("distributive","full factorial","capped factorial","parallel","single trial")[1])

{

probs = 1-1/(odds+1) # change those ugly odds into probs

interesting_combinations_byspec =

lapply(strsplit(names(true_effects)[names(true_effects)!="--"],"-"),

function(xx)

{

aa = sort(xx[xx!=""]) # for each combination, list of involved candidates ; convoluted to go this way but this piece of code may be useful later

if(length(aa))

{

bb = matrix(matrix(as.logical(intToBits(-1+1:2**length(aa))),nrow=32)[1:length(aa),],nrow=length(aa))

cc = apply(bb,2,function(xx){paste("",paste(aa[xx],collapse="-"),"",sep="-")})

} else { cc="--" }

cc

})

interesting_combinations_overall = names(table(unlist(interesting_combinations_byspec))) # just calling this on a strsplit is better

interesting_combinations_overall = interesting_combinations_overall[interesting_combinations_overall!="--"]

interesting_candidates = names(table(unlist(strsplit(interesting_combinations_overall,"-"))))

interesting_candidates = as.numeric(interesting_candidates[interesting_candidates!=""])

if(modality=="single trial") { output = cbind("-1-"=c(TRUE,FALSE),p=c(probs[1],1-probs[1])) }

if(modality=="parallel") { output = cbind(rbind(0,diag(length(probs))),c(1,odds)) ; colnames(output) = c(paste("-",1:length(probs),"-",sep=""),"p") ; output[,"p"]=output[,"p"]/sum(output[,"p"]) }

if(any(modality==c("distributive","full factorial","capped factorial")))

{

interesting_candidates_binary_draws = matrix(matrix(as.logical(intToBits(-1+1:2**length(interesting_candidates))),nrow=32)[1:length(interesting_candidates),],nrow=length(interesting_candidates))

rownames(interesting_candidates_binary_draws) = interesting_candidates

interesting_candidates_binary_draws_probabilities = apply(interesting_candidates_binary_draws, 2, function(xx){ prod(probs[interesting_candidates][xx],1-probs[interesting_candidates][!xx]) } )

uninteresting_candidates_draws = c(1,rep(0,allocs+1)) ; names(uninteresting_candidates_draws) = c(0:allocs,"too many")

for(ii in (1:length(probs))[-interesting_candidates])

{

uninteresting_candidates_draws = (1-probs[ii])*uninteresting_candidates_draws + probs[ii]*c(0,uninteresting_candidates_draws[-(allocs+2)]) + c(rep(0,allocs+1),probs[ii]*uninteresting_candidates_draws["too many"])

} # maybe something faster here by treating this as markov chain and multiplying matrices together beforehand, but this looks okay efficiency-wise given expectation for allocs

# for each number of interesting candidates drawn, probability to have picked an allowed number of uninteresting ones

if(modality=="full factorial") { prob_allowed_number_uninteresting_drawn = rep(1,1+length(interesting_candidates)) }

if(modality=="capped factorial") { prob_allowed_number_uninteresting_drawn = sapply(0:min(allocs,length(interesting_candidates)),function(xx) { sum(uninteresting_candidates_draws[1:(allocs-xx+1)]) }) }

if(modality=="distributive") { prob_allowed_number_uninteresting_drawn = sapply(0:min(allocs,length(interesting_candidates)),function(xx) { uninteresting_candidates_draws[allocs-xx+1] }) }

if(length(interesting_candidates)>allocs&(modality=="distributive"|modality=="capped factorial")) { prob_allowed_number_uninteresting_drawn = c(prob_allowed_number_uninteresting_drawn,rep(0,length(interesting_candidates)-allocs)) }

names(prob_allowed_number_uninteresting_drawn) = 0:length(interesting_candidates)

probs_interesting_allocations = sapply(1:ncol(interesting_candidates_binary_draws),function(ii) { interesting_candidates_binary_draws_probabilities[ii] * prob_allowed_number_uninteresting_drawn[1+sum(interesting_candidates_binary_draws[,ii])] })

probs_interesting_allocations = probs_interesting_allocations/sum(probs_interesting_allocations)

output = t(interesting_candidates_binary_draws)

colnames(output) = paste("-",colnames(output),"-",sep="")

output = cbind(output,p=probs_interesting_allocations)

}

output=cbind(output,sapply(interesting_combinations_overall[sapply(interesting_combinations_overall,function(xx){length(strsplit(xx,"-")[[1]])})>2],

function(xx){apply(matrix(as.logical(output[,paste("-",strsplit(xx,"-")[[1]][-1],"-",sep="")]),nrow=nrow(output)),1,all)}))

true_effects = true_effects[order(names(true_effects))]

true_effects = true_effects[order(nchar(names(true_effects)))]

s = rep(true_effects["--"],nrow(output))

for(ii in names(true_effects)[names(true_effects)!="--"])

{

s[as.logical(output[,ii])] = true_effects[ii] # set the outcome ; this can overwrite values several times, up to the largest specified combination (which is the right one in this specification)

}

output = cbind(output,s)

output = output[,order(nchar(colnames(output)))]

output

}

aa = focused_allocations()

## #Sample size and power for difference of proportions

chain_size = function(mm, of_interest = colnames(mm)[-(1:2)], powers=0.9, nominal_alphas=0.05, bonferroni = rep(length(of_interest),length(of_interest)))

{

alphas = nominal_alphas/bonferroni

if(length(alphas)<length(of_interest)) { alphas = c(alphas,rep(rev(alphas)[1],length(of_interest)-length(alphas))) }

names(alphas) = of_interest

if(length(powers)<length(of_interest)) { powers = c(powers,rep(rev(powers)[1],length(of_interest)-length(powers))) }

names(powers) = of_interest

output = sapply(of_interest,function(xx)

{

treated = matrix(c(mm[mm[,xx]==TRUE,1:2]),ncol=2)

untreated = matrix(c(mm[mm[,xx]==FALSE,1:2]),ncol=2)

bsamsize(weighted.mean(unlist(treated[,2]),unlist(treated[,1])),weighted.mean(unlist(untreated[,2]),unlist(untreated[,1])),sum(unlist(treated[,1])), alpha=alphas[xx], power=powers[xx])

})

names(output) = of_interest

output

}

chain_power = function(mm, sampsize, of_interest = colnames(mm)[-(1:2)], nominal_alphas = 0.05, bonferroni = rep(length(of_interest),length(of_interest)))

{

alphas = nominal_alphas/bonferroni

if(length(alphas)<length(of_interest)) { alphas = c(alphas,rep(rev(alphas)[1],length(of_interest)-length(alphas))) }

names(alphas) = of_interest

output = sapply(of_interest,function(xx)

{

treated = matrix(mm[mm[,xx]==TRUE,1:2],ncol=2)

untreated = matrix(mm[mm[,xx]==FALSE,1:2],ncol=2)

bpower(weighted.mean(unlist(treated[,2]),unlist(treated[,1])),weighted.mean(unlist(untreated[,2]),unlist(untreated[,1])),sum(unlist(treated[,1])),sampsize,alpha=alphas[xx])

})

names(output) = of_interest

output

}

integrated_calc = function(truth_integ,NN=4,allocs_integ=2,alpha_integ=0.05,bonferroni_integ=1,odds_integ=rep(1,NN),modality_integ="distributive",of_interest_integ="-1-",proportion_true_control=0)

{

arm_descriptor = focused_allocations(odds=odds_integ, allocs=allocs_integ, true_effects=truth_integ, modality=modality_integ)

if(proportion_true_control!=0) { arm_descriptor[,1] = arm_descriptor[,1]*(1-proportion_true_control) ; arm_descriptor = rbind(arm_descriptor,c(proportion_true_control,truth_integ["--"],rep(0,ncol(arm_descriptor)-2))) }

sizes_integ = chain_size(arm_descriptor,of_interest=of_interest_integ,nominal_alphas=alpha_integ,bonferroni=bonferroni_integ)

powers_integ = chain_power(arm_descriptor,min(sizes_integ,na.rm=T),of_interest=of_interest_integ,nominal_alphas=alpha_integ,bonferroni=bonferroni_integ)

output = list("0",arm_descriptor,sizes_integ[which.min(sizes_integ)],powers_integ)

names(output) = c("Call","Allocations-outcomes","Size","Power")

output$Call = paste0(modality_integ, " design with ",NN," interventions with successes ", paste(names(truth_integ),round(truth_integ,2), sep=" ", collapse=" ")," and ", allocs_integ, " allocations")

output

}

## #Logistic regression algorithms (different strategies)

logreg_mains = function(XX)

{

pvals = summary(glm(as.formula(paste0("yy ~ ",paste(colnames(XX)[-1],collapse=" + "))), family=binomial(logit),data=XX))$coefficients[,"Pr(>|z|)"]

pvals_output = rep(1,ncol(XX)-2)

names(pvals_output) = paste0("X",-2+3:ncol(XX))

pvals_output[] = pvals[names(pvals_output)]

pvals_output[is.na(pvals_output)] = 1

pvals_output

}

logreg_mains_ixn12 = function(XX)

{

pvals=summary(glm(as.formula(paste0("yy ~ ",paste(colnames(XX)[-1],collapse=" + ")," + X1:X2")), family=binomial(logit),data=XX))$coefficients[,"Pr(>|z|)"]

pvals_output = rep(1,ncol(XX)-2)

names(pvals_output) = paste0("X",-2+3:ncol(XX))

pvals_output[] = pvals[names(pvals_output)]

pvals_output[is.na(pvals_output)] = 1

pvals_output

}

logreg_mains_ixn12desc05 = function(XX)

{

pvals=summary(glm(as.formula(paste0("yy ~ ",paste(colnames(XX)[-1],collapse=" + ")," + X1:X2")), family=binomial(logit),data=XX))$coefficients[,"Pr(>|z|)"]

if(!is.na(pvals["X1:X2"])&pvals["X1:X2"]>.05) pvals=summary(glm(as.formula(paste0("yy ~ ",paste(colnames(XX)[-1],collapse=" + "),"")), family=binomial(logit),data=XX))$coefficients[,"Pr(>|z|)"]

pvals_output = rep(1,ncol(XX)-2)

names(pvals_output) = paste0("X",-2+3:ncol(XX))

pvals_output[] = pvals[names(pvals_output)]

pvals_output[is.na(pvals_output)] = 1

pvals_output

}

logreg_mains_ixn12desc15 = function(XX)

{

pvals=summary(glm(as.formula(paste0("yy ~ ",paste(colnames(XX)[-1],collapse=" + ")," + X1:X2")), family=binomial(logit),data=XX))$coefficients[,"Pr(>|z|)"]

if(!is.na(pvals["X1:X2"])&pvals["X1:X2"]>.15) pvals=summary(glm(as.formula(paste0("yy ~ ",paste(colnames(XX)[-1],collapse=" + "),"")), family=binomial(logit),data=XX))$coefficients[,"Pr(>|z|)"]

pvals_output = rep(1,ncol(XX)-2)

names(pvals_output) = paste0("X",-2+3:ncol(XX))

pvals_output[] = pvals[names(pvals_output)]

pvals_output[is.na(pvals_output)] = 1

pvals_output

}

logreg_mains_all_ixn = function(XX)

{

all_pairs_twice = sapply(colnames(XX)[-1],function(xx) colnames(XX)[-1][colnames(XX)[-1]!=xx] ) # interactions

all_ixns_once = unique(c(sapply(colnames(all_pairs_twice), function(xx) apply(cbind(xx,all_pairs_twice[,xx]),1,function(yy) paste(sort(yy),collapse=":") ) )))

pvals=summary(glm(as.formula(paste0("yy ~ ",paste(colnames(XX)[-1],collapse=" + ")," + ",paste(all_ixns_once,collapse=" + "))), family=binomial(logit),data=XX))$coefficients[,"Pr(>|z|)"]

pvals_output = rep(1,ncol(XX)-2)

names(pvals_output) = paste0("X",-2+3:ncol(XX))

pvals_output[] = pvals[names(pvals_output)]

pvals_output[is.na(pvals_output)] = 1

pvals_output

}

logreg_mains_ixn_1all = function(XX)

{

all_ixns_once = paste(colnames(XX)[2],colnames(XX)[-c(1,2,ncol(XX))],sep=":")

pvals=summary(glm(as.formula(paste0("yy ~ ",paste(colnames(XX)[-1],collapse=" + ")," + ",paste(all_ixns_once,collapse=" + "))), family=binomial(logit),data=XX))$coefficients[,"Pr(>|z|)"]

pvals_output = rep(1,ncol(XX)-2)

names(pvals_output) = paste0("X",-2+3:ncol(XX))

pvals_output[] = pvals[names(pvals_output)]

pvals_output[is.na(pvals_output)] = 1

pvals_output

}

logreg_mains_ixn_Adesc = function(XX,pthreshold=.25)

{

all_pairs_twice = sapply(colnames(XX)[-1],function(xx) colnames(XX)[-1][colnames(XX)[-1]!=xx] ) # interactions

all_ixns_once = unique(c(sapply(colnames(all_pairs_twice), function(xx) apply(cbind(xx,all_pairs_twice[,xx]),1,function(yy) paste(sort(yy),collapse=":") ) )))

eligible_ixns = all_ixns_once

finished=F

while(!finished)

{

pvals=summary(glm(as.formula(paste0("yy ~ ",paste(colnames(XX)[-1],collapse=" + ")," + ",paste(eligible_ixns,collapse=" + "))), family=binomial(logit),data=XX))$coefficients[,"Pr(>|z|)"]

pvals_ixns = rep(1,length(eligible_ixns)) ; names(pvals_ixns) = eligible_ixns

pvals_ixns[] = pvals[names(pvals_ixns)]

pvals_ixns[is.na(pvals_ixns)] = 1

if(all(pvals_ixns<pthreshold)|!any(pvals_ixns<pthreshold)) finished=T

eligible_ixns = eligible_ixns[pvals_ixns<pthreshold]

}

pvals=summary(glm(as.formula(paste0("yy ~ ",paste(colnames(XX)[-1],collapse=" + "),paste(rep(" + ",length(eligible_ixns)),eligible_ixns,sep="",collapse=""))), family=binomial(logit),data=XX))$coefficients[,"Pr(>|z|)"]

pvals_output = rep(1,ncol(XX)-2)

names(pvals_output) = paste0("X",-2+3:ncol(XX))

pvals_output[] = pvals[names(pvals_output)]

pvals_output[is.na(pvals_output)] = 1

pvals_output

}

logreg_mains_ixn_Adesc_005 = function(XX) logreg_mains_ixn_Adesc(XX,pthreshold=.05)

logreg_mains_ixn_1desc = function(XX,pthreshold=.25)

{

all_ixns_once = paste(colnames(XX)[2],colnames(XX)[-c(1,2,ncol(XX))],sep=":")

eligible_ixns = all_ixns_once

finished=F

while(!finished)

{

pvals=summary(glm(as.formula(paste0("yy ~ ",paste(colnames(XX)[-1],collapse=" + ")," + ",paste(eligible_ixns,collapse=" + "))), family=binomial(logit),data=XX))$coefficients[,"Pr(>|z|)"]

pvals_ixns = rep(1,length(eligible_ixns)) ; names(pvals_ixns) = eligible_ixns

pvals_ixns[] = pvals[names(pvals_ixns)]

pvals_ixns[is.na(pvals_ixns)] = 1

if(all(pvals_ixns<pthreshold)|!any(pvals_ixns<pthreshold)) finished=T

eligible_ixns = eligible_ixns[pvals_ixns<pthreshold]

}

pvals=summary(glm(as.formula(paste0("yy ~ ",paste(colnames(XX)[-1],collapse=" + "),paste(rep(" + ",length(eligible_ixns)),eligible_ixns,sep="",collapse=""))), family=binomial(logit),data=XX))$coefficients[,"Pr(>|z|)"]

pvals_output = rep(1,ncol(XX)-2)

names(pvals_output) = paste0("X",-2+3:ncol(XX))

pvals_output[] = pvals[names(pvals_output)]

pvals_output[is.na(pvals_output)] = 1

pvals_output

}

logreg_mains_ixn_1desc_005 = function(XX) logreg_mains_ixn_1desc(XX,pthreshold=.05)

## #Power by simulation

simulate_power = function(odds=c(1,1,1,1,1,1,1,1,1,1), allocs=4, sampsize=2000, nsims = 1000, true_effects = c("-1-2-3-"=0.9,"-1-"=0.7,"--"=0.5), nominal_alphas = 0.05, bonferroni = NA, modality=c("distributive","full factorial")[1], analysis_algorithm = logreg_mains, proportion_true_control = 0)

{

if(is.na(bonferroni)) { bonferroni = rep(length(odds),length(odds)) }

nonmodal = which(odds!=names(table(odds))[which.max(table(odds))])

if(length(nonmodal))

{

names(nonmodal) = paste0("-",nonmodal,"-")

nonmodal[] = true_effects["--"]

nonmodal=nonmodal[!sapply(names(nonmodal), function(xx) { any(xx==names(true_effects)) })]

}

# here we add skewedly allocated treatments to true_effects to have them be computed explicitly by the other function

drawing_table = focused_allocations(odds=odds,allocs=allocs,true_effects=c(true_effects,nonmodal),modality=modality)

if(mode(drawing_table)=="list") { backup = dimnames(drawing_table) ; drawing_table = matrix(unlist(drawing_table),nrow=nrow(drawing_table)) ; dimnames(drawing_table) = backup }

drawing_table = drawing_table[,sapply(colnames(drawing_table),function(xx){length(gregexpr("-",xx)[[1]])<3})] # kick out combinations, they are not useful for simulations

remaining_draws = (1:length(odds))[-as.numeric(gsub("-","",colnames(drawing_table)[-(1:2)]))]

# capped factorial needs to keep track of probability for each number of allowed remaining draws, not implemented yet

# will need the same workflow as focused_allocations but pulling out the probabilities for each number of possible remaining draws

# maybe can add an option to focused_allocations to output this as well for capped factorial

#print(drawing_table)

#clusterExport(workingcluster,c("drawing_table","sampsize","remaining_draws","modality","allocs","odds","analysis_algorithm"), envir=environment() )

simulations = sapply(1:nsims,function(xx)#parSapply(workingcluster,1:nsims,function(xx)

{

partial_draw = cbind(1:nrow(drawing_table),rmultinom(1,round(sampsize*(1-proportion_true_control)),drawing_table[,"p"]))

partial_draw = drawing_table[unlist(apply(partial_draw,1,function(yy){rep(yy[1],yy[2])})),]

if(length(remaining_draws)>0)

{

if(modality=="distributive") simdata = cbind(partial_draw,matrix(sapply(as.numeric(rownames(partial_draw)),function(yy){sample(c(rep(1,allocs-yy),rep(0,length(remaining_draws)-allocs+yy)))}),byrow=T,nrow=nrow(partial_draw)))

if(modality=="full factorial") simdata = cbind(partial_draw,matrix(runif(nrow(partial_draw)*length(remaining_draws))<(1-1/(mean(odds[remaining_draws])+1)),nrow=nrow(partial_draw)))

colnames(simdata) = c(colnames(partial_draw),paste0("-",remaining_draws,"-"))

} else { simdata = partial_draw }

if(proportion_true_control != 0) simdata = rbind(simdata,matrix(c(0,true_effects["--"],rep(0,ncol(simdata)-2)),nrow=sampsize-nrow(simdata),ncol=ncol(simdata),byrow=T)) #adding some controls

# we are not modifying simdata$s to include an effect for the remaining draws, future versions may

simdata = simdata[,-1]

simdata[,"s"] = runif(nrow(simdata))<simdata[,"s"]

colnames(simdata)=c("yy",paste0("X",gsub("-","",colnames(simdata)[-1])))

colnames(simdata)[colnames(simdata)=="X"]=paste0("X",remaining_draws)

simdata = data.frame(simdata[,c("yy",paste0("X",1:length(odds)))])

backup = dimnames(simdata) ; simdata = data.frame(matrix(unlist(simdata),nrow=nrow(simdata))) ; dimnames(simdata) = backup

rownames(simdata) = paste0("P",1:nrow(simdata))

analysis_algorithm(simdata)

})

#print(simulations)

output = sapply(1:nrow(simulations),function(xx){mean(!is.na(simulations[xx,])&simulations[xx,]<nominal_alphas/bonferroni[xx])})

output

}

## #Sample size by simulation

simulate_sampsize = function(odds=rep(1,10), allocs=4, tar_power=0.9, nsims=5000, true_effects = c("-1-2-3-"=0.9,"-1-"=0.7,"--"=0.5), nominal_alphas = 0.05, bonferroni = NA, modality=c("distributive","full factorial")[1], analysis_algorithm = logreg_mains, verbose=F, proportion_true_control=0)

{

if(is.na(bonferroni)) { bonferroni = rep(length(odds),length(odds)) }

# see above for the first computations

nonmodal = which(odds!=names(table(odds))[which.max(table(odds))])

if(length(nonmodal))

{

names(nonmodal) = paste0("-",nonmodal,"-")

nonmodal[] = true_effects["--"]

nonmodal=nonmodal[!sapply(names(nonmodal), function(xx) { any(xx==names(true_effects)) })]

}

drawing_table = focused_allocations(odds=odds,allocs=allocs,true_effects=c(true_effects,nonmodal),modality=modality)

if(mode(drawing_table)=="list") { backup = dimnames(drawing_table) ; drawing_table = matrix(unlist(drawing_table),nrow=nrow(drawing_table)) ; dimnames(drawing_table) = backup }

drawing_table = drawing_table[,sapply(colnames(drawing_table),function(xx){length(gregexpr("-",xx)[[1]])<3})] # kick out combinations, they are not useful for simulations

remaining_draws = (1:length(odds))[-as.numeric(gsub("-","",colnames(drawing_table)[-(1:2)]))]

# here is where it starts being different

# we are going to compute different sample sizes only a few times and then fit a logistic regression

# this will enable picking a new range of values, and we keep doing this until our estimate for the sample size giving desired poser converges

tarNN = round(exp(seq(log(50),log(10000),length.out=48)))

latest_estimate = sqrt(10000)

latest_beta=.1

trials = matrix(c(1,sqrt(1000000),0,1),nrow=2)

colnames(trials) = c("srsampsize","nullreject")

se = NA

intercept_target = qnorm(tar_power)

trimmed_initiation = FALSE

#clusterExport(workingcluster,c("drawing_table","remaining_draws","modality","allocs","odds","analysis_algorithm"), envir=environment() )

while(nrow(trials)<nsims) #abs(latest_estimate^2-older_estimate^2)/latest_estimate^2>0.01|is.na(se)|se>.3)

{

cat("-")

simulations = sapply(tarNN,function(NN)#parSapply(workingcluster,tarNN,function(NN)

{

partial_draw = cbind(1:nrow(drawing_table),rmultinom(1,round(NN*(1-proportion_true_control)),drawing_table[,"p"]))

partial_draw = drawing_table[unlist(apply(partial_draw,1,function(yy){rep(yy[1],yy[2])})),]

if(length(remaining_draws)>0)

{

if(modality=="distributive") simdata = cbind(partial_draw,matrix(sapply(as.numeric(rownames(partial_draw)),function(yy){sample(c(rep(1,allocs-yy),rep(0,length(remaining_draws)-allocs+yy)))}),byrow=T,nrow=nrow(partial_draw)))

if(modality=="full factorial") simdata = cbind(partial_draw,matrix(runif(nrow(partial_draw)*length(remaining_draws))<(1-1/(mean(odds[remaining_draws])+1)),nrow=nrow(partial_draw)))

colnames(simdata) = c(colnames(partial_draw),paste0("-",remaining_draws,"-"))

} else { simdata = partial_draw }

if(proportion_true_control != 0) simdata = rbind(simdata,matrix(c(0,true_effects["--"],rep(0,ncol(simdata)-2)),nrow=NN-nrow(simdata),ncol=ncol(simdata),byrow=T)) #adding some controls

simdata = simdata[,-1]

simdata[,"s"] = runif(nrow(simdata))<simdata[,"s"]

colnames(simdata)=c("yy",paste0("X",gsub("-","",colnames(simdata)[-1])))

colnames(simdata)[colnames(simdata)=="X"]=paste0("X",remaining_draws)

simdata = data.frame(simdata[,c("yy",paste0("X",1:length(odds)))])

backup = dimnames(simdata) ; simdata = data.frame(matrix(unlist(simdata),nrow=nrow(simdata))) ; dimnames(simdata) = backup

rownames(simdata) = paste0("P",1:nrow(simdata))

analysis_algorithm(simdata)[1]<nominal_alphas/bonferroni[1] # non-convergence of too large trials interpreted as failure so strange behavior can happen

})

if(nrow(trials)<200&nrow(trials)>50) cat(round(median(tarNN)))

if(nrow(trials)>200&!trimmed_initiation) { trials=trials[-(1:100),] ; trimmed_initiation = TRUE } # this prevents getting stuck because of an unlucky initiation

plot(trials[,1]^2,xlim=c(0,nsims*1.1),ylim=quantile(trials[,1],c(.01,.99))^2,main=c("Trace of sample size exploration",paste0("Current estimate ",round(latest_estimate^2),", slope ",round(latest_beta,3))),xlab="Simulation#",ylab="Sample size",type="p",col=2+trials[,2],pch=3,cex=.25+.25*(1-trials[,2]))

trials = rbind(trials,cbind(srsampsize=sqrt(tarNN),simulations))

loglik = function(xx) { sum(log( (1-trials[,"nullreject"]) + (trials[,"nullreject"]-.5)*2*pnorm(intercept_target+xx["srbeta"]^2*(trials[,"srsampsize"]-xx["X_target"])))) }

if(verbose) { print(trials[nrow(trials)-2:0,]) ; cat("\n") ; print(c("beta"=latest_beta, "X_target"=latest_estimate)) }

initial_value = loglik(c("srbeta"=.3, "X_target"=mean(trials[,"srsampsize"])))

if(initial_value!=Inf&initial_value!=-Inf&!is.na(initial_value))

{

try(mle <- optim(c("srbeta"=.3, "X_target"=mean(trials[,"srsampsize"])), fn=loglik, hessian = TRUE, control=list(fnscale=-1)), silent = TRUE)

latest_beta = unname(mle$par["srbeta"]^2)

latest_estimate = unname(mle$par["X_target"])

if(det(mle$hessian)!=0) { se = sqrt(diag(solve(-mle$hessian)))["X_target"] } else {se = 10}

if(is.na(se)){se=NULL}

tarNN = pmax(7,latest_estimate+c(-1,1)*min(10,se)) # span a window of +- 1 SE except if SE too large, minimum trial size 49

tarNN = round(seq(tarNN[1],tarNN[2],length.out=max(50,round(nrow(trials)/10)))^2) # in increments of 10% of the number of existing data points, evenly spread

} else {

tarNN = round(seq(quantile(trials[,1],.01),quantile(trials[,1],.99),length.out=max(50,round(nrow(trials)/10)))^2)

latest_estimate = 30

latest_beta=0.3

cat("LLfail")

} # the else loop may rarely be needed to unstick the algorithm

if(verbose) { cat("\n") ; print(tarNN); cat("\n") ; print(se) ; cat("\n") }

}

cat("sample size found : ", round(latest_estimate^2), " with ",nrow(trials), " simulated trials", "\n")

round(latest_estimate^2)

}

# #Scripts to reproduce the main figures

## #Compute and draw Figure 2

if(!any(ls()=="topdf")) topdf = T

if(topdf) pdf("Figure analytical proportions.pdf",width=7*2,height=7*3)

par(mfrow=c(3,2),cex=1.35,cex.lab=1,cex.axis=1)

####################################################

# 1-20 treatments, one works

####################################################

#

treatments_eff = c(0.7,0.5) ; names(treatments_eff) = c("-1-","--")

results = matrix(rep(NA,120),nrow=6)

rownames(results) = c("Distributive","Full factorial*","Capped factorial","Parallel arms (pooled)","Parallel arms","Separate trials")

modalities_list = c("distributive","full factorial","capped factorial","parallel","single trial","single trial")

for(ii in 1:6) { for(jj in 4:20)

{

results[ii,jj] = integrated_calc(treatments_eff,NN=jj,allocs_integ=2,bonferroni_integ=jj,modality_integ=modalities_list[ii])$Size

}}

results["Parallel arms",] = results["Parallel arms",]/2*((1:ncol(results))+1) # Obtained from a H2H trial, /2 for arm size, multiply by Ntreatments+1

results["Separate trials",] = results["Separate trials",]*(1:ncol(results)) # Need to run several separate trials, not one

####################################################

# Plot the base case of 4 treatments

####################################################

#

xx = results[,4]

names(xx) = c("Distributive","Full factorial*","Capped factorial","Parallel arms","Parallel arms","Separate trials")

xx = xx[c(2,1,3:6)]

plot(-1000,-1000,xlim=c(-.75,1)*1500, ylim=0:1*7, main="Apply 0-2 interventions among 4 candidates\nOnly 1 effective (from 50% to 70% success)", cex.main=0.9, xaxt="n",yaxt="n",bty="n",xlab="",ylab="")

mtext("A.",side=3,line=-3,outer=T,at=0,cex=4,adj=0)

for(ii in 6:1)

{

polygon(0+c(0,0,1,1)*xx[ii],-1+ii+(ii>4)*.5+c(0,1,1,0),col=cc[c(2,1,3:6)][ii])

text(-.95*max(xx,na.rm=T),-.5+ii+(ii>4)*.5,names(xx)[ii],pos=4)

}

axis(1, las=1, line=0.5,at=c(0,500,1000,1500))

mtext("Versus\ncontrol",2,line=1,at=5.5)

mtext("__________",2,line=.5,at=5.5)

mtext("Pooled\nanalysis",2,line=1,at=2)

mtext("_____________________",2,line=.5,at=2)

mtext("Total size of trial(s)",1,3,at=750,cex=1.2)

####################################################

# Plot ratio to parallel-arm

####################################################

#

par(mar=c(4 , 4, 4, 6))

plot(0,0,xlim=c(4,20),ylim=c(floor(log(results["Full factorial*",ncol(results)]/results["Parallel arms",ncol(results)])/log(2)),ceiling(log(results["Separate trials",ncol(results)]/results["Parallel arms",ncol(results)])/log(2))),col=0,main="Gain relative to unpooled parallel-arm",cex.main=0.9,xlab="Candidate interventions",ylab="Sample size ratio",xaxt="n",yaxt="n",bty="n")

mtext("B.",side=3,line=-3,outer=T,at=0.5,cex=4,adj=0)

for(ii in 6:1)

{

lines(log(results[ii,]/results["Parallel arms",])/log(2),type="o",pch=19,col=cc[ii],lwd=2)

}

axis(1, las=1, line=0.5,at=c(4,8,12,16,20))

axis(2, labels=rep("",3), las=0, line=0.5,at=log(c(0.06,0.25,1))/log(2),tck=-0.10/2,cex.axis=1)

axis(2, labels=rep("",3), las=0, line=0.5,at=log(c(0.12,0.5,2))/log(2),tck=-0.03/2,cex.axis=1)

mtext(c(".06",".12",".25",".5","1","2"),side=2,line=0.8*c(2.2,1,2.2,1,2.2,1),at=log(c(.06,.12,.25,.5,1,2))/log(2),cex=1.2)

axis(4, labels=paste0(c(".5",1,2,4,10),"x"), las=1, line=0.5,at=log(1/c(0.5,1,2,4,10))/log(2),cex.axis=1)

mtext("Gain", side=4, line=3,cex=1.2)

par(mar=c(4 , 4, 4, 4))

####################################################

# Plot as function of candidates, overall

####################################################

#

plot(0,0,xlim=c(4,20),ylim=c(0,9000),col=0, main="Raw sizes", cex.main=0.9,xlab="Candidate interventions",ylab="Total size of trial(s)",xaxt="n",yaxt="n",bty="n")

for(ii in 1:9) lines(c(2,20),1000*c(ii,ii)-1000,lty=c(1,2,2,2,1,2,2,2,1)[ii],col="lightgrey")

mtext("C.",side=3,line=-1*26-3,outer=T,at=0,cex=4,adj=0)

polygon(c(2.5,10.5,10.5,2.5),c(0,0,2000,2000),col="lightgrey",border="lightgrey",lwd=2)

for(ii in 1:3) lines(c(2.5,10.5),1000*c(ii,ii)-1000,lty=c(1,2,2,2,1,2,2,2,1)[ii],col="white")

for(ii in 6:1)

{

lines(results[ii,],type="o",col=cc[ii],pch=19,lwd=2)

}

axis(1, las=1, line=0.5,at=c(4,8,12,16,20))

axis(2, las=0, line=0.5,at=c(0,4000,8000))

####################################################

# Plot as function of candidates, zoomed

####################################################

#

plot(0,0,xlim=c(3.5,10.5),ylim=c(0,2000),col=0, main="Raw sizes (zoomed)", cex.main=0.9,xlab="Candidate interventions",ylab="Total size of trial(s)",xaxt="n",yaxt="n",bty="n")

mtext("D.",side=3,line=-1*26-3,outer=T,at=0.5,cex=4,adj=0)

polygon(c(2.5,10.5,10.5,2.5),c(0,0,2000,2000),col="lightgrey",border="lightgrey",lwd=2)

for(ii in 1:9) lines(c(2.5,10.5),250*c(ii,ii)-250,lty=c(1,2,2,2,1,2,2,2,1)[ii],col="white")

for(ii in 6:1)

{

lines(results[ii,],type="o",col=cc[ii],pch=19,lwd=2)

}

axis(1, las=1, line=0.5,at=c(4,6,8,10))

axis(2, las=0, line=0.5,at=c(0,1000,2000))

####################################################

# 1-20 treatments, one works

####################################################

#

treatments_eff = c(0.7,0.5) ; names(treatments_eff) = c("-1-","--")

results = matrix(rep(NA,120),nrow=6)

rownames(results) = c("Distributive","Full factorial*","Capped factorial","Parallel arms (pooled)","Parallel arms","Separate trials")

modalities_list = c("distributive","full factorial","capped factorial","parallel","single trial","single trial")

for(ii in 1:6) { for(jj in 6:20)

{

results[ii,jj] = integrated_calc(treatments_eff,NN=jj,allocs_integ=4,bonferroni_integ=jj,modality_integ=modalities_list[ii])$Size

}}

results["Parallel arms",] = results["Parallel arms",]/2*((1:ncol(results))+1) # Obtained from a H2H trial, /2 for arm size, multiply by Ntreatments+1

results["Separate trials",] = results["Separate trials",]*(1:ncol(results)) # Need to run several separate trials, not one

####################################################

# Plot ratio to parallel-arm

####################################################

#

par(mar=c(4 , 4, 4, 6))

plot(0,0,xlim=c(6,20),ylim=c(floor(log(results["Full factorial*",ncol(results)]/results["Parallel arms",ncol(results)])/log(2)),ceiling(log(results["Separate trials",ncol(results)]/results["Parallel arms",ncol(results)])/log(2))),col=0,main="Gain versus parallel-arm\n4 allocations",cex.main=0.9,xlab="Candidate interventions",ylab="Sample size ratio",xaxt="n",yaxt="n",bty="n")

mtext("E.",side=3,line=-2*26-3,outer=T,at=0,cex=4,adj=0)

for(ii in 6:1)

{

lines(log(results[ii,]/results["Parallel arms",])/log(2),type="o",pch=19,col=cc[ii],lwd=2)

}

axis(1, las=1, line=0.5,at=c(6,8,10,12,14,16,18,20))

axis(2, labels=rep("",3), las=0, line=0.5,at=log(c(0.06,0.25,1))/log(2),tck=-0.10/2,cex.axis=1)

axis(2, labels=rep("",3), las=0, line=0.5,at=log(c(0.12,0.5,2))/log(2),tck=-0.03/2,cex.axis=1)

mtext(c(".06",".12",".25",".5","1","2"),side=2,line=0.8*c(2.2,1,2.2,1,2.2,1),at=log(c(.06,.12,.25,.5,1,2))/log(2),cex=1.2)

axis(4, labels=paste0(c(".5",1,2,4,10),"x"), las=1, line=0.5,at=log(1/c(0.5,1,2,4,10))/log(2),cex.axis=1)

mtext("Gain", side=4, line=3,cex=1.2)

par(mar=c(4 , 4, 4, 4))

####################################################

# 1-20 treatments, one works

####################################################

#

treatments_eff = c(0.7,0.5) ; names(treatments_eff) = c("-1-","--")

results = matrix(rep(NA,120),nrow=6)

rownames(results) = c("Distributive","Full factorial*","Capped factorial","Parallel arms (pooled)","Parallel arms","Separate trials")

modalities_list = c("distributive","full factorial","capped factorial","parallel","single trial","single trial")

for(ii in 1:6) { for(jj in 10:20)

{

if(!(ii==1&jj<9)) { results[ii,jj] = integrated_calc(treatments_eff,NN=jj,allocs_integ=8,bonferroni_integ=jj,modality_integ=modalities_list[ii])$Size }

}}

results["Parallel arms",] = results["Parallel arms",]/2*((1:ncol(results))+1) # Obtained from a H2H trial, /2 for arm size, multiply by Ntreatments+1

results["Separate trials",] = results["Separate trials",]*(1:ncol(results)) # Need to run several separate trials, not one

####################################################

# Plot ratio to parallel-arm

####################################################

#

par(mar=c(4 , 4, 4, 6))

plot(0,0,xlim=c(10,20),ylim=c(floor(log(results["Full factorial*",ncol(results)]/results["Parallel arms",ncol(results)])/log(2)),ceiling(log(results["Separate trials",ncol(results)]/results["Parallel arms",ncol(results)])/log(2))),col=0,main="Gain versus parallel-arm\n8 allocations",cex.main=0.9,xlab="Candidate interventions",ylab="Sample size ratio",xaxt="n",yaxt="n",bty="n")

mtext("F.",side=3,line=-2*26-3,outer=T,at=0.5,cex=4,adj=0)

for(ii in 6:1)

{

lines(log(results[ii,]/results["Parallel arms",])/log(2),type="o",pch=19,col=cc[ii],lwd=2)

}

axis(1, las=1, line=0.5,at=c(10,12,14,16,18,20))

axis(2, labels=rep("",3), las=0, line=0.5,at=log(c(0.06,0.25,1))/log(2),tck=-0.10/2,cex.axis=1)

axis(2, labels=rep("",3), las=0, line=0.5,at=log(c(0.12,0.5,2))/log(2),tck=-0.03/2,cex.axis=1)

mtext(c(".06",".12",".25",".5","1","2"),side=2,line=0.8*c(2.2,1,2.2,1,2.2,1),at=log(c(.06,.12,.25,.5,1,2))/log(2),cex=1.2)

axis(4, labels=paste0(c(".5",1,2,4,10),"x"), las=1, line=0.5,at=log(1/c(0.5,1,2,4,10))/log(2),cex.axis=1)

mtext("Gain", side=4, line=3,cex=1.2)

par(mar=c(4 , 4, 4, 4))

if(topdf) dev.off()

## #Generate data for Figures 3 and 4

treatments_eff_assumed = c("--"=0.5,"-1-"=0.7)

treatments_eff_true = c("--"=0.5,"-1-"=0.7,"-2-"=0.7,"-1-2-"=0.8448276)

if(any(list.files()=="figdata_no_ixn.bak")) load("figdata_no_ixn.bak")

situations = c("sizes_scenario0_assumed",

"sizes_scenario1_assumed",

"sizes_scenario2_assumed",

"sizes_scenario3_assumed",

"sizes_scenario4_assumed",

"power_scenario0_assumed_scenario0_true",

"power_scenario0_assumed_scenario1_true",

"power_scenario0_assumed_scenario2_true",

"power_scenario0_assumed_scenario3_true",

"power_scenario0_assumed_scenario4_true")

if(!any(ls()=="figdata_no_ixn"))

{

figdata_no_ixn = list(list(fullfactorial=rep(NA,20),distributive=matrix(NA,nrow=8,ncol=20)))[rep(1,length(situations))]

figdata_no_ixn[[1]][["distributive"]] = matrix(NA,nrow=19,ncol=20)

names(figdata_no_ixn)=situations

}

#if(!any(ls()=="figdata_no_ixn_sizes_wrong_assumption")) figdata_no_ixn_sizes_wrong_assumption = list(fullfactorial=rep(NA,20),distributive=matrix(rep(NA,8*20),nrow=8))

assumptions = list(

c("--"=0.5,"-1-"=0.7),

c("--"=0.5,"-1-"=0.7,"-2-"=0.7,"-1-2-"=0.8448276),

c("--"=0.5,"-1-"=0.7,"-2-"=0.7,"-1-2-"=.99), # changed because convergence issues with 100%

c("--"=0.5,"-1-"=0.7,"-2-"=0.7,"-1-2-"=0.7),

c("--"=0.5,"-1-"=0.7,"-2-"=0.6,"-1-2-"=0.7) )

names(assumptions) = paste0("scenario",0:4)

for(scenario in names(assumptions))

{

situation = paste0("sizes_",scenario,"_assumed")

for(ii in 2:nrow(figdata_no_ixn[[situation]][["distributive"]])) { for(jj in (ii+1):20)

{

if((scenario=="scenario2"|scenario=="scenario3"|scenario=="scenario4")&(jj-ii)==1&is.na(figdata_no_ixn[[situation]][["distributive"]][ii,jj])) { figdata_no_ixn[[situation]][["distributive"]][ii,jj] = -1 } # logistic cannot converge in this case, too many parameters

cat(scenario,",",ii,"allocations among ",jj,"\n")

if(is.na(figdata_no_ixn[[situation]][["distributive"]][ii,jj]))

{

figdata_no_ixn[[situation]][["distributive"]][ii,jj] = simulate_sampsize(odds=rep(1,jj),

allocs=ii,

nsims=5000,

true_effects = assumptions[[scenario]],

nominal_alphas=0.05,

modality=c("distributive","full factorial")[1])

}

if(is.na(figdata_no_ixn[[situation]][["fullfactorial"]][jj]))

{

figdata_no_ixn[[situation]][["fullfactorial"]][jj] = simulate_sampsize(odds=rep(1,jj),

allocs=ii,

nsims=5000,

true_effects = assumptions[[scenario]],

nominal_alphas=0.05,

modality=c("distributive","full factorial")[2]) }

} ; save(figdata_no_ixn,file="figdata_no_ixn.bak") }

}

for(situation in rev(rev(situations)[1:5]))

{

expected_scenario = gsub("power_","",strsplit(situation,"_assumed_")[[1]][1])

true_scenario = gsub("_true","",strsplit(situation,"_assumed_")[[1]][2])

for(ii in 2:8) { for(jj in (ii+1):20)

{

cat(expected_scenario,"expected,",true_scenario,"true,",ii,"allocations among ",jj,"\n")

if(is.na(figdata_no_ixn[[situation]][["distributive"]][ii,jj])&!is.na(figdata_no_ixn[[paste0("sizes_",expected_scenario,"_assumed")]][["distributive"]][ii,jj])&figdata_no_ixn[[paste0("sizes_",expected_scenario,"_assumed")]][["distributive"]][ii,jj]!=-1)

{

power_output = simulate_power(odds=rep(1,jj),

allocs=ii,

sampsize=figdata_no_ixn[[paste0("sizes_",expected_scenario,"_assumed")]][["distributive"]][ii,jj],

nsims=5000,

true_effects = assumptions[[true_scenario]],

nominal_alphas=0.05,

modality=c("distributive","full factorial")[1])

print(power_output)

figdata_no_ixn[[situation]][["distributive"]][ii,jj] = power_output[1]

}

if(is.na(figdata_no_ixn[[situation]][["fullfactorial"]][jj]))

{

cat(expected_scenario,"expected,",true_scenario,"true,","factorial allocations among ",jj,"\n")

power_output = simulate_power(odds=rep(1,jj),

allocs=ii,

sampsize=figdata_no_ixn[[paste0("sizes_",expected_scenario,"_assumed")]][["fullfactorial"]][jj],

nsims=5000,

true_effects = assumptions[[true_scenario]],

nominal_alphas=0.05,

modality=c("distributive","full factorial")[2])

print(power_output)

figdata_no_ixn[[situation]][["fullfactorial"]][jj] = power_output[1]

}

} ; save(figdata_no_ixn,file="figdata_no_ixn.bak") }

}

## #Draw Figures 3 and 4

load("figdata_no_ixn.bak")

situations = c("sizes_scenario0_assumed",

"sizes_scenario1_assumed",

"sizes_scenario2_assumed",

"sizes_scenario3_assumed",

"sizes_scenario4_assumed",

"power_scenario0_assumed_scenario0_true",

"power_scenario0_assumed_scenario1_true",

"power_scenario0_assumed_scenario2_true",

"power_scenario0_assumed_scenario3_true",

"power_scenario0_assumed_scenario4_true")

if(!any(ls()=="topdf")) topdf = T

if(topdf) pdf("Figure - distributive main effect testing only main figure.pdf",width=7*2,height=7*3)

par(mfrow=c(3,2),cex=1.35,cex.lab=1,cex.axis=1)

palette_anchors = rbind(c(0,0,0,0.5),c(1,.8,.8,.8))

cc = apply(color_spread(20,palette_anchors),2,function(xx){rgb(xx[1],xx[2],xx[3])})

plot(0,0,col=0,xlim=0:1,ylim=0:1*max(figdata_no_ixn$sizes_scenario0_assumed$distributive,na.rm=T), xlab="Proportion allocated per subject (k/K)",ylab="Trial size",main="Choosing the number of allocations", cex.main=0.9, bty="n")

mtext("A.",side=3,line=-3,outer=T,at=0,cex=4,adj=0)

for(jj in rev(c(5,10,15,20))) lines(0:1*.1+.45,rep(figdata_no_ixn$sizes_scenario0_assumed$fullfactorial[jj],2),type="l",pch=20,cex=0.5,col="grey",lwd=2)

for(jj in rev(c(5,10,15,20))) { kk = 2:(jj-2) ; lines(kk/jj,figdata_no_ixn$sizes_scenario0_assumed$distributive[kk,jj],type="o",pch=20,lwd=2,col=cc[jj-1]) }

legend(0.5,max(figdata_no_ixn$sizes_scenario0_assumed$distributive,na.rm=T),c("",5,10,15,20),pch=19,lwd=2,col=c(0,cc[4],cc[9],cc[15],cc[19]),inset=0.01,bty="n",cex=0.9,xjust=0.5,yjust=1)

legend(0.5,max(figdata_no_ixn$sizes_scenario0_assumed$distributive,na.rm=T),"K =",pch=19,lwd=2,col=c(0),inset=0.01,bty="n",cex=0.9,xjust=1,yjust=1)

palette_anchors = rbind(c(0,.3,.0,.9),c(1,.9,.7,.0))

cc = apply(color_spread(8,palette_anchors),2,function(xx){rgb(xx[1],xx[2],xx[3])})

plot(0,0,col=0,xlim=c(4,20),ylim=0:1*max(figdata_no_ixn$sizes_scenario0_assumed$distributive,na.rm=T),xlab="Number of candidates (K)",ylab="Trial size",main="Choosing the number of candidate interventions", cex.main=0.9, bty="n",xaxt="n")

axis(1, las=1, line=0.5,at=seq(4,20,4))

mtext("B.",side=3,line=-3,outer=T,at=0.5,cex=4,adj=0)

lines(4:20,figdata_no_ixn$sizes_scenario0_assumed$fullfactorial[4:20],type="l",pch=20,cex=0.5,col="grey",lwd=2)

for(ii in rev(c(2,4,6,8))) lines((ii+2):20,figdata_no_ixn$sizes_scenario0_assumed$distributive[ii,(ii+2):20],type="o",pch=20,lwd=2,col=cc[ii-1])

legend(10,max(figdata_no_ixn$sizes_scenario0_assumed$distributive,na.rm=T),c("",2,4,6,8),pch=19,lwd=2,col=c(0,cc[1],cc[3],cc[5],cc[7]),inset=0.01,bty="n",cex=0.9,xjust=0.5,yjust=1)

legend(10,max(figdata_no_ixn$sizes_scenario0_assumed$distributive,na.rm=T),"k =",pch=19,lwd=2,col=c(0),inset=0.01,bty="n",cex=0.9,xjust=1,yjust=1)

if(!topdf) dev.new()

if(!topdf) par(mfrow=c(2,2),cex=1.35,cex.lab=1,cex.axis=1)

scenario_names = c("Both main effects 70%, logit-additivity","Both main effects 70%, synergy 99%","Both main effects 70%, no additivity","Occult main effect 60%, no additivity")

for(kk in 1:4)

{

situation = rev(rev(situations)[1:4])[kk]

palette_anchors = rbind(c(0.0,0.0+0*kk,0.0+0*kk,0.5-0*kk),c(1,0.+0*kk,0.8-0*kk,0.8+0*kk))

cc = apply(color_spread(8,palette_anchors),2,function(xx){rgb(xx[1],xx[2],xx[3])})

plot(0,0,col=0,xlim=c(4,20),ylim=0:1,xlab="Number of candidates",ylab="Statistical power",main=scenario_names[kk], cex.main=0.9, bty="n",xaxt="n")

axis(1, las=1, line=0.5,at=seq(4,20,4))

mtext(c("C.","D.","E.","F.")[kk],side=3,line=-(1+(kk-1)%/%2)*26-3,outer=T,at=c(0,.5,0,.5)[kk],cex=4,adj=0)

lines(4:20,figdata_no_ixn[[situation]][["fullfactorial"]][4:20],type="l",pch=20,cex=0.5,col="grey",lwd=2)

for(ii in 2:8) lines((ii+2):20,figdata_no_ixn[[situation]][["distributive"]][ii,(ii+2):20],type="o",pch=20,lwd=2,col=cc[ii-1])

if(kk==1) legend(20,0,c("From 2 allocations... ","... to 8 (per patient)"),pch=19,lwd=2,col=c(cc[1],cc[7]),inset=0.01,bty="n",cex=0.9,xjust=1,yjust=0,title="Color gradient",title.adj=0)

}

if(topdf) dev.off() else dev.new()

if(topdf) pdf("Figure - distributive main effect testing only sampsize shifts.pdf",width=7*2,height=7*2)

par(mfrow=c(2,2),cex=1.35,cex.lab=1,cex.axis=1)

scenario_names = c("Both main effects 70%, logit-additivity","Both main effects 70%, synergy 99%","Both main effects 70%, no additivity","Occult main effect 60%, no additivity")

for(kk in 1:4)

{

situation = situations[1+kk]

palette_anchors = rbind(c(0.0,0.8+0*kk,0.5+0*kk,0.0-0*kk),c(1,0.8+0*kk,0.0-0*kk,0.0+0*kk))

cc = apply(color_spread(8,palette_anchors),2,function(xx){rgb(xx[1],xx[2],xx[3])})

plot(0,0,col=0,xlim=c(4,20),ylim=c(-2,4),xlab="Number of candidates",ylab="Sample size fold-change",main=scenario_names[kk], cex.main=0.9, bty="n",yaxt="n",xaxt="n")

axis(1, las=1, line=0.5,at=seq(4,20,4))

mtext(c("A.","B.","C.","D.")[kk],side=3,line=-(0+(kk-1)%/%2)*26-3,outer=T,at=c(0,.5,0,.5)[kk],cex=4,adj=0)

axis(2,at=-2:4,labels=c("¼","½",1,2,4,8,16))

lines(log(figdata_no_ixn[[situation]][["fullfactorial"]]/figdata_no_ixn[["sizes_scenario0_assumed"]][["fullfactorial"]])/log(2),type="l",pch=20,cex=0.5,col="grey",lwd=2)

for(ii in 2:8)

{

figdata_no_ixn[[situation]][["distributive"]][ii,!is.na(figdata_no_ixn[[situation]][["distributive"]][ii,]) & figdata_no_ixn[[situation]][["distributive"]][ii,] == -1] = NA

lines((ii+2):20,log(figdata_no_ixn[[situation]][["distributive"]][ii,(ii+2):20]/figdata_no_ixn[["sizes_scenario0_assumed"]][["distributive"]][ii,(ii+2):20])/log(2),type="o",pch=20,lwd=2,col=cc[ii-1])

}

if(kk==1) legend(20,4,c("From 2 allocations... ","... to 8 (per patient)"),pch=19,lwd=2,col=c(cc[1],cc[7]),inset=0.01,bty="n",cex=0.9,xjust=1,yjust=1,title="Color gradient",title.adj=0)

}

if(topdf) dev.off()

## #Generate data for Figure 5

if(any(list.files()=="figdata_ixntests_truecontrols.bak")) load("figdata_ixntests_truecontrols.bak")

ixn_strats = strsplit("confound logreg_mains logreg_mains_ixn12desc05 logreg_mains_ixn12desc15 logreg_mains_ixn12 logreg_mains_ixn_1desc logreg_mains_ixn_1desc_005 logreg_mains_ixn_Adesc_005"," ")[[1]] # logreg_mains_ixn_1all logreg_mains_ixn_Adesc logreg_mains_all_ixn

control_percents = paste0("C",c("00","05","10","20","30","40","60","80"))

if(!any(ls()=="figdata_ixntests_truecontrols")) figdata_ixntests_truecontrols = array(NA,dim=c(length(ixn_strats),length(control_percents),2,2),dimnames=list(ixn_strats,control_percents,c("nonadd","add"),1:2))

for(ii in ixn_strats[-1]) { for(jj in control_percents) { for(kk in c("nonadd","add")) { for(ll in 1:2)

{

cat("algorithm",ii,"true controls",jj, "scenario", kk,"dilution", ll, "\n")

if(is.na(figdata_ixntests_truecontrols[ii,jj,kk,ll])) figdata_ixntests_truecontrols[ii,jj,kk,ll] = simulate_sampsize(odds=rep(1,c(10,20)[ll]),allocs=c(4,2)[ll], true_effects = list(nonadd=c("--"=.5,"-1-"=.7,"-2-"=.7,"-1-2-"=.7),add=c("--"=.5,"-1-"=.7,"-2-"=.7,"-1-2-"=.8448276))[[kk]], nominal_alphas=0.05, modality="distributive", analysis_algorithm = get(ii), proportion_true_control = as.numeric(gsub("C","",jj))/100)

save(figdata_ixntests_truecontrols,file="figdata_ixntests_truecontrols.bak")

}}}}

for(jj in control_percents) { for(kk in c("nonadd","add")) { for(ll in 1:2)

{

figdata_ixntests_truecontrols["confound",jj,kk,ll] = round(integrated_calc(list(nonadd=c("--"=.5,"-1-"=.7,"-2-"=.7,"-1-2-"=.7),add=c("--"=.5,"-1-"=.7,"-2-"=.7,"-1-2-"=.8448276))[[kk]],NN=c(10,20)[ll],allocs_integ=c(4,2)[ll],bonferroni_integ=c(10,20)[ll],modality_integ="distributive",proportion_true_control = as.numeric(gsub("C","",jj))/100)$Size)

}}}

save(figdata_ixntests_truecontrols,file="figdata_ixntests_truecontrols.bak")

figdata_ixntests_truecontrols_confound = array(NA,dim=c(81,2,2),dimnames=list(paste0("C",0:80),c("nonadd","add"),1:2))

for(jj in dimnames(figdata_ixntests_truecontrols_confound)[[1]]) { for(kk in c("nonadd","add")) { for(ll in 1:2)

{

figdata_ixntests_truecontrols_confound[jj,kk,ll] = round(integrated_calc(list(nonadd=c("--"=.5,"-1-"=.7,"-2-"=.7,"-1-2-"=.7),add=c("--"=.5,"-1-"=.7,"-2-"=.7,"-1-2-"=.8448276))[[kk]],NN=c(10,20)[ll],allocs_integ=c(4,2)[ll],bonferroni_integ=c(10,20)[ll],modality_integ="distributive",proportion_true_control = as.numeric(gsub("C","",jj))/100)$Size)

}}}

## #Draw Figure 5

load("figdata_ixntests_truecontrols.bak")

displayed_strats = strsplit("confound logreg_mains logreg_mains_ixn12 logreg_mains_ixn12desc05 logreg_mains_ixn12desc15 logreg_mains_ixn_1desc_005 logreg_mains_ixn_1desc"," ")[[1]] # logreg_mains_ixn_1all logreg_mains_desc_ixn logreg_mains_all_ixn

control_percents = paste0("C",c("00","05","10","20","30","40","60","80"))

if(!any(ls()=="topdf")) topdf = T

if(topdf) pdf("Figure control arm and testing strategies.pdf",width=7*2,height=7*2)

par(mfrow=c(2,2),cex=1.35,cex.lab=1,cex.axis=1)

palette_anchors = rbind(c(0,1,.0,.0),c(.167,1,.5,.5),c(.333,.8,.4,.8),c(.5,.5,.5,1),c(.667,.0,.2,1),c(.833,.8,.8,.8),c(1,.3,.3,.3))

cc = apply(color_spread(length(displayed_strats),palette_anchors),2,function(xx){rgb(xx[1],xx[2],xx[3])})

#cc=c("red","orange","grey","purple","blue")

names(cc) = displayed_strats

plot(0,0,col=0,xlim=0:1,ylim=0:1*max(figdata_no_ixn$sizes_scenario0_assumed$distributive,na.rm=T), xlab="Proportion allocated to true control (zero intervention)",ylab="Trial size",main="4 among 10, logit-scale additivity", cex.main=0.9, bty="n")

mtext("A.",side=3,line=-3,outer=T,at=0,cex=4,adj=0)

for(jj in displayed_strats[-1]) lines(as.numeric(gsub("C","",control_percents))/100, figdata_ixntests_truecontrols[jj,,"add",1],type="o",pch=20,lwd=2,col=cc[jj])

lines(as.numeric(gsub("C","",dimnames(figdata_ixntests_truecontrols_confound)[[1]]))/100,figdata_ixntests_truecontrols_confound[,"add",1],col=cc["confound"],lwd=3)

legend(0.22,max(figdata_no_ixn$sizes_scenario0_assumed$distributive,na.rm=T)*1.1,c("Confound","No interaction","Pre-specified","Pre-specified if p<0.05","Pre-specified if p<0.15","Backward p<0.05","Backward p<0.25"),pch=c(NA,rep(19,length(displayed_strats)-1)),lwd=c(3,rep(2,length(displayed_strats)-1)),col=cc,inset=0.01,bty="n",cex=0.8,xjust=0.5,yjust=1,title=NA)

plot(0,0,col=0,xlim=0:1,ylim=0:1*max(figdata_no_ixn$sizes_scenario0_assumed$distributive,na.rm=T), xlab="Proportion allocated to control (zero intervention)",ylab="Trial size",main="4 among 10, no additivity", cex.main=0.9, bty="n")

mtext("B.",side=3,line=-3,outer=T,at=0.5,cex=4,adj=0)

for(jj in displayed_strats[-1]) lines(as.numeric(gsub("C","",control_percents))/100, figdata_ixntests_truecontrols[jj,,"nonadd",1],type="o",pch=20,lwd=2,col=cc[jj])

lines(as.numeric(gsub("C","",dimnames(figdata_ixntests_truecontrols_confound)[[1]]))/100,figdata_ixntests_truecontrols_confound[,"nonadd",1],col=cc["confound"],lwd=3)

plot(0,0,col=0,xlim=0:1,ylim=0:1*max(figdata_no_ixn$sizes_scenario0_assumed$distributive,na.rm=T), xlab="Proportion allocated to control (zero intervention)",ylab="Trial size",main="2 among 20, logit-scale additivity", cex.main=0.9, bty="n")

mtext("C.",side=3,line=-3-26,outer=T,at=0,cex=4,adj=0)

for(jj in displayed_strats[-1]) lines(as.numeric(gsub("C","",control_percents))/100, figdata_ixntests_truecontrols[jj,,"add",2],type="o",pch=20,lwd=2,col=cc[jj])

lines(as.numeric(gsub("C","",dimnames(figdata_ixntests_truecontrols_confound)[[1]]))/100,figdata_ixntests_truecontrols_confound[,"add",2],col=cc["confound"],lwd=3)

plot(0,0,col=0,xlim=0:1,ylim=0:1*max(figdata_no_ixn$sizes_scenario0_assumed$distributive,na.rm=T), xlab="Proportion allocated to control (zero intervention)",ylab="Trial size",main="2 among 20, no additivity", cex.main=0.9, bty="n")

mtext("D.",side=3,line=-3-26,outer=T,at=0.5,cex=4,adj=0)

for(jj in displayed_strats[-1]) lines(as.numeric(gsub("C","",control_percents))/100, figdata_ixntests_truecontrols[jj,,"nonadd",2],type="o",pch=20,lwd=2,col=cc[jj])

lines(as.numeric(gsub("C","",dimnames(figdata_ixntests_truecontrols_confound)[[1]]))/100,figdata_ixntests_truecontrols_confound[,"nonadd",2],col=cc["confound"],lwd=3)

if(topdf) dev.off()

# #Scripts to reproduce the supplementary figures

#some of these scripts only work if the main figure data has been generated (e.g. sample sizes for simulations) and/or if previous scripts have been called

## #Simulate and draw Supplementary Figure S1

if(0){

source("./sup/1- Simulate and draw power and FWER one-sided for Fig2.r")

}

source("1- Clean setup.R")

if(!any(ls()=="topdf")) topdf = T

####################################################

# Define the function

####################################################

#

simulate_one = function(NN=1000,Nmaxalloc=2,Ncandidates=20,strategy=c("Distributive","Full factorial*","Capped factorial","Parallel arms (pooled)","Parallel arms","Separate trials")[1])

{

if(strategy=="Full factorial*") designmat = matrix(rbinom(NN*Ncandidates,1,0.5),nrow=NN)

if(strategy=="Capped factorial") designmat = t(replicate(NN,sample(c(rep(1,which(rmultinom(1,1,dbinom(0:Nmaxalloc,Ncandidates,.5))==1)-1),rep(0,Ncandidates))[1:Ncandidates]))) # the multinom part draws how many allocations in a truncated distribution (rather than defaulting to Nmaxalloc if above) ; both are possible but the paper was initially written such

if(strategy=="Distributive") designmat = t(replicate(NN,sample(c(rep(1,Nmaxalloc),rep(0,Ncandidates-Nmaxalloc)))))

if(grepl("Parallel arms",strategy)) designmat = t(replicate(NN,sample(c(1,rep(0,Ncandidates)))[1:Ncandidates]))

if(strategy!="Separate trials"&strategy!="Parallel arms")

{

yy = runif(NN)<(0.5+0.2*designmat[,1])

colnames(designmat) = paste0("X",1:Ncandidates)

output = sapply(colnames(designmat),function(xx){prop.test(matrix(c(sum(yy[designmat[,xx]==1]),sum(yy[designmat[,xx]==0]),sum(1-yy[designmat[,xx]==1]),sum(1-yy[designmat[,xx]==0])),nrow=2),correct=F,alternative=propalternative)$p.value})

}

if(strategy=="Parallel arms")

{

yy = runif(NN)<(0.5+0.2*designmat[,1])

colnames(designmat) = paste0("X",1:Ncandidates)

output = sapply(colnames(designmat),function(xx){prop.test(matrix(c(sum(yy[designmat[,xx]==1]),sum(yy[rowSums(designmat)==0]),sum(1-yy[designmat[,xx]==1]),sum(1-yy[rowSums(designmat)==0])),nrow=2),correct=F,alternative=propalternative)$p.value})

}

if(strategy=="Separate trials")

{

aa = paste0("X",1:Ncandidates)

output = sapply(aa,function(xx)

{

bb = round(NN/Ncandidates/2) # per arm

cc = rbinom(2,bb,c(0.5+0.2*(xx=="X1"),0.5)) # successes

cc = cbind(cc,bb-cc)

prop.test(cc,alternative=propalternative,correct=F)$p.value

})

}

output

}

####################################################

# Create object with results

####################################################

#

if(!any(list.files()=="fwercheck analytic prop.bak")) { power_fwer_results = list(NULL) } else { load(file="fwercheck analytic prop.bak") }

Nsims = 1000

########################################################################

# For 2 allocations max (ssizes as in main article, then power and fwer)

########################################################################

#

if(is.null(power_fwer_results[["powersims_2_g"]])|is.null(power_fwer_results[["fwersims_2_g"]]))

{

treatments_eff = c(0.7,0.5) ; names(treatments_eff) = c("-1-","--")

results = matrix(rep(NA,120),nrow=6)

rownames(results) = c("Distributive","Full factorial*","Capped factorial","Parallel arms (pooled)","Parallel arms","Separate trials")

modalities_list = c("distributive","full factorial","capped factorial","parallel","single trial","single trial")

for(ii in 1:6) { for(jj in 3:20)

{

results[ii,jj] = integrated_calc(treatments_eff,NN=jj,allocs_integ=2,bonferroni_integ=jj,modality_integ=modalities_list[ii])$Size

}}

results["Parallel arms",] = results["Parallel arms",]/2*((1:ncol(results))+1) # Obtained from a H2H trial, /2 for arm size, multiply by Ntreatments+1

results["Separate trials",] = results["Separate trials",]*(1:ncol(results)) # Need to run several separate trials, not one

powersims = results*NA

fwersims = powersims

propalternative="g"

for(jj in 3:20) { for(ii in rownames(results))

{

sims = replicate(Nsims,simulate_one(NN=round(results[ii,jj]),Ncandidates=jj,strategy=ii))

powersims[ii,jj] = mean(sims[1,]<0.025/jj)

fwersims[ii,jj] = mean(as.logical(colSums(sims[-1,]<0.025/jj)))

cat(ii,jj,powersims[ii,jj],fwersims[ii,jj],"\n")

}}

power_fwer_results[["powersims_2_g"]] = powersims

power_fwer_results[["fwersims_2_g"]] = fwersims

save(power_fwer_results,file="fwercheck analytic prop.bak")

}

if(topdf) pdf("Figure - Simulated power and FWER for analytical propdiff comparisons.pdf",width=7*2,height=7*3)

par(mfrow=c(3,2),cex=1.35,cex.lab=1,cex.axis=1)

plot(0,0,xlim=c(3,20),ylim=c(0.85,1),col=0, main="Power (max 2 allocations, one-sided test)", cex.main=0.9,xlab="Candidate interventions",ylab=paste("Power in", Nsims, "simulations"),bty="n")

for(ii in 1:nrow(results)) lines(3:20+0.03*(ii-nrow(results)/2-0.5),power_fwer_results[["powersims_2_g"]][ii,3:20],type="o",pch=20,col=cc[ii])

for(ii in 1:nrow(results)) { for(jj in 3:20) { pp = power_fwer_results[["powersims_2_g"]][ii,jj] ; lines(rep(jj+0.03*(ii-nrow(results)/2-0.5),2),qbeta(c(0.025,0.975),Nsims*pp+0.5,Nsims*(1-pp)+0.5),col=cc[ii]) }}

lines(c(3,20),c(0.9,0.9),lty=2,col="lightgrey")

legend(20,1,rownames(results),pch=19,lwd=2,col=cc,inset=0.01,bty="n",cex=0.9,xjust=1,yjust=1)

mtext("A.",side=3,line=-3,outer=T,at=0,cex=4,adj=0)

plot(0,0,xlim=c(3,20),ylim=c(0,0.15),col=0, main="Cognate FWER among null candidates", cex.main=0.9,xlab="Candidate interventions",ylab=paste("FWER in the same", Nsims, "simulations"),bty="n")

for(ii in 1:nrow(results)) lines(3:20+0.03*(ii-nrow(results)/2-0.5),power_fwer_results[["fwersims_2_g"]][ii,3:20],type="o",pch=20,col=cc[ii])

for(ii in 1:nrow(results)) { for(jj in 3:20) { pp = power_fwer_results[["fwersims_2_g"]][ii,jj] ; lines(rep(jj+0.03*(ii-nrow(results)/2-0.5),2),qbeta(c(0.025,0.975),Nsims*pp+0.5,Nsims*(1-pp)+0.5),col=cc[ii]) }}

lines(c(3,20),c(0.025,0.025),lty=2,col="lightgrey")

mtext("B.",side=3,line=-3,outer=T,at=0.5,cex=4,adj=0)

#####################################################

# For 4 allocations max (ssizes, then power and fwer)

#####################################################

#

if(is.null(power_fwer_results[["powersims_4_g"]])|is.null(power_fwer_results[["fwersims_4_g"]]))

{

treatments_eff = c(0.7,0.5) ; names(treatments_eff) = c("-1-","--")

results = matrix(rep(NA,120),nrow=6)

rownames(results) = c("Distributive","Full factorial*","Capped factorial","Parallel arms (pooled)","Parallel arms","Separate trials")

modalities_list = c("distributive","full factorial","capped factorial","parallel","single trial","single trial")

for(ii in 1:6) { for(jj in 5:20)

{

results[ii,jj] = integrated_calc(treatments_eff,NN=jj,allocs_integ=4,bonferroni_integ=jj,modality_integ=modalities_list[ii])$Size

}}

results["Parallel arms",] = results["Parallel arms",]/2*((1:ncol(results))+1) # Obtained from a H2H trial, /2 for arm size, multiply by Ntreatments+1

results["Separate trials",] = results["Separate trials",]*(1:ncol(results)) # Need to run several separate trials, not one

powersims = results*NA

fwersims = powersims

propalternative="g"

for(jj in 5:20) { for(ii in rownames(results))

{

sims = replicate(Nsims,simulate_one(NN=round(results[ii,jj]),Nmaxalloc=4,Ncandidates=jj,strategy=ii))

powersims[ii,jj] = mean(sims[1,]<0.025/jj)

fwersims[ii,jj] = mean(as.logical(colSums(sims[-1,]<0.025/jj)))

cat(ii,jj,powersims[ii,jj],fwersims[ii,jj],"\n")

}}

power_fwer_results[["powersims_4_g"]] = powersims

power_fwer_results[["fwersims_4_g"]] = fwersims

save(power_fwer_results,file="fwercheck analytic prop.bak")

}

plot(0,0,xlim=c(3,20),ylim=c(0.85,1),col=0, main="Power (max 4 allocations, one-sided test)", cex.main=0.9,xlab="Candidate interventions",ylab=paste("Power in", Nsims, "simulations"),bty="n")

for(ii in 1:nrow(results)) lines(5:20+0.03*(ii-nrow(results)/2-0.5),power_fwer_results[["powersims_4_g"]][ii,5:20],type="o",pch=20,col=cc[ii])

for(ii in 1:nrow(results)) { for(jj in 5:20) { pp = power_fwer_results[["powersims_4_g"]][ii,jj] ; lines(rep(jj+0.03*(ii-nrow(results)/2-0.5),2),qbeta(c(0.025,0.975),Nsims*pp+0.5,Nsims*(1-pp)+0.5),col=cc[ii]) }}

lines(c(3,20),c(0.9,0.9),lty=2,col="lightgrey")

mtext("C.",side=3,line=-1*26-3,outer=T,at=0,cex=4,adj=0)

plot(0,0,xlim=c(3,20),ylim=c(0,0.15),col=0, main="Cognate FWER among null candidates", cex.main=0.9,xlab="Candidate interventions",ylab=paste("FWER in the same", Nsims, "simulations"),bty="n")

for(ii in 1:nrow(results)) lines(5:20+0.03*(ii-nrow(results)/2-0.5),power_fwer_results[["fwersims_4_g"]][ii,5:20],type="o",pch=20,col=cc[ii])

for(ii in 1:nrow(results)) { for(jj in 5:20) { pp = power_fwer_results[["fwersims_4_g"]][ii,jj] ; lines(rep(jj+0.03*(ii-nrow(results)/2-0.5),2),qbeta(c(0.025,0.975),Nsims*pp+0.5,Nsims*(1-pp)+0.5),col=cc[ii]) }}

lines(c(3,20),c(0.025,0.025),lty=2,col="lightgrey")

mtext("D.",side=3,line=-1*26-3,outer=T,at=0.5,cex=4,adj=0)

#####################################################

# For 8 allocations max (ssizes, then power and fwer)

#####################################################

#

if(is.null(power_fwer_results[["powersims_8_g"]])|is.null(power_fwer_results[["fwersims_8_g"]]))

{

treatments_eff = c(0.7,0.5) ; names(treatments_eff) = c("-1-","--")

results = matrix(rep(NA,120),nrow=6)

rownames(results) = c("Distributive","Full factorial*","Capped factorial","Parallel arms (pooled)","Parallel arms","Separate trials")

modalities_list = c("distributive","full factorial","capped factorial","parallel","single trial","single trial")

for(ii in 1:6) { for(jj in 9:20)

{

results[ii,jj] = integrated_calc(treatments_eff,NN=jj,allocs_integ=8,bonferroni_integ=jj,modality_integ=modalities_list[ii])$Size

}}

results["Parallel arms",] = results["Parallel arms",]/2*((1:ncol(results))+1) # Obtained from a H2H trial, /2 for arm size, multiply by Ntreatments+1

results["Separate trials",] = results["Separate trials",]*(1:ncol(results)) # Need to run several separate trials, not one

powersims = results*NA

fwersims = powersims

propalternative="g"

for(jj in 9:20) { for(ii in rownames(results))

{

sims = replicate(Nsims,simulate_one(NN=round(results[ii,jj]),Nmaxalloc=8,Ncandidates=jj,strategy=ii))

powersims[ii,jj] = mean(sims[1,]<0.025/jj)

fwersims[ii,jj] = mean(as.logical(colSums(sims[-1,]<0.025/jj)))

cat(ii,jj,powersims[ii,jj],fwersims[ii,jj],"\n")

}}

power_fwer_results[["powersims_8_g"]] = powersims

power_fwer_results[["fwersims_8_g"]] = fwersims

save(power_fwer_results,file="fwercheck analytic prop.bak")

}

plot(0,0,xlim=c(3,20),ylim=c(0.85,1),col=0, main="Power (max 8 allocations, one-sided test)", cex.main=0.9,xlab="Candidate interventions",ylab=paste("Power in", Nsims, "simulations"),bty="n")

for(ii in 1:nrow(results)) lines(9:20+0.03*(ii-nrow(results)/2-0.5),power_fwer_results[["powersims_8_g"]][ii,9:20],type="o",pch=20,col=cc[ii])

for(ii in 1:nrow(results)) { for(jj in 9:20) { pp = power_fwer_results[["powersims_8_g"]][ii,jj] ; lines(rep(jj+0.03*(ii-nrow(results)/2-0.5),2),qbeta(c(0.025,0.975),Nsims*pp+0.5,Nsims*(1-pp)+0.5),col=cc[ii]) }}

lines(c(3,20),c(0.9,0.9),lty=2,col="lightgrey")

mtext("E.",side=3,line=-2*26-3,outer=T,at=0,cex=4,adj=0)

plot(0,0,xlim=c(3,20),ylim=c(0,0.15),col=0, main="Cognate FWER among null candidates", cex.main=0.9,xlab="Candidate interventions",ylab=paste("FWER in the same", Nsims, "simulations"),bty="n")

for(ii in 1:nrow(results)) lines(9:20+0.03*(ii-nrow(results)/2-0.5),power_fwer_results[["fwersims_8_g"]][ii,9:20],type="o",pch=20,col=cc[ii])

for(ii in 1:nrow(results)) { for(jj in 9:20) { pp = power_fwer_results[["fwersims_8_g"]][ii,jj] ; lines(rep(jj+0.03*(ii-nrow(results)/2-0.5),2),qbeta(c(0.025,0.975),Nsims*pp+0.5,Nsims*(1-pp)+0.5),col=cc[ii]) }}

lines(c(3,20),c(0.025,0.025),lty=2,col="lightgrey")

mtext("F.",side=3,line=-2*26-3,outer=T,at=0.5,cex=4,adj=0)

if(topdf) dev.off()

## #Simulate and draw Supplementary Figure S2

if(0){

source("./sup/2- Two-sided FWER for figure 2.r")

}

if(!any(ls()=="topdf")) topdf = F

####################################################

# Create object with results

####################################################

#

if(!any(list.files()=="fwercheck analytic prop twosided.bak")) { power_fwer_results = list(NULL) } else { load(file="fwercheck analytic prop twosided.bak") }

Nsims = 1000

########################################################################

# For 2 allocations max (ssizes as in main article, then power and fwer)

########################################################################

#

if(is.null(power_fwer_results[["powersims_2_g"]])|is.null(power_fwer_results[["fwersims_2_g"]]))

{

treatments_eff = c(0.7,0.5) ; names(treatments_eff) = c("-1-","--")

results = matrix(rep(NA,120),nrow=6)

rownames(results) = c("Distributive","Full factorial*","Capped factorial","Parallel arms (pooled)","Parallel arms","Separate trials")

modalities_list = c("distributive","full factorial","capped factorial","parallel","single trial","single trial")

for(ii in 1:6) { for(jj in 3:20)

{

results[ii,jj] = integrated_calc(treatments_eff,NN=jj,allocs_integ=2,bonferroni_integ=jj,modality_integ=modalities_list[ii])$Size

}}

results["Parallel arms",] = results["Parallel arms",]/2*((1:ncol(results))+1) # Obtained from a H2H trial, /2 for arm size, multiply by Ntreatments+1

results["Separate trials",] = results["Separate trials",]*(1:ncol(results)) # Need to run several separate trials, not one

powersims = results*NA

fwersims = powersims

propalternative="t"

for(jj in 3:20) { for(ii in rownames(results))

{

sims = replicate(Nsims,simulate_one(NN=round(results[ii,jj]),Ncandidates=jj,strategy=ii))

powersims[ii,jj] = mean(sims[1,]<0.05/jj)

fwersims[ii,jj] = mean(as.logical(colSums(sims[-1,]<0.05/jj)))

cat(ii,jj,powersims[ii,jj],fwersims[ii,jj],"\n")

}}

power_fwer_results[["powersims_2_g"]] = powersims

power_fwer_results[["fwersims_2_g"]] = fwersims

save(power_fwer_results,file="fwercheck analytic prop twosided.bak")

}

if(topdf) pdf("Figure - Simulated power and FWER for analytical propdiff comparisons twosided.pdf",width=7*2,height=7*3)

par(mfrow=c(3,2),cex=1.35,cex.lab=1,cex.axis=1)

plot(0,0,xlim=c(3,20),ylim=c(0.85,1),col=0, main="Power (max 2 allocations, two-sided test)", cex.main=0.9,xlab="Candidate interventions",ylab=paste("Power in", Nsims, "simulations"),bty="n")

for(ii in 1:nrow(results)) lines(3:20+0.03*(ii-nrow(results)/2-0.5),power_fwer_results[["powersims_2_g"]][ii,3:20],type="o",pch=20,col=cc[ii])

for(ii in 1:nrow(results)) { for(jj in 3:20) { pp = power_fwer_results[["powersims_2_g"]][ii,jj] ; lines(rep(jj+0.03*(ii-nrow(results)/2-0.5),2),qbeta(c(0.025,0.975),Nsims*pp+0.5,Nsims*(1-pp)+0.5),col=cc[ii]) }}

lines(c(3,20),c(0.9,0.9),lty=2,col="lightgrey")

legend(20,1,rownames(results),pch=19,lwd=2,col=cc,inset=0.01,bty="n",cex=0.9,xjust=1,yjust=1)

mtext("A.",side=3,line=-3,outer=T,at=0,cex=4,adj=0)

plot(0,0,xlim=c(3,20),ylim=c(0,0.15),col=0, main="Cognate FWER among null candidates", cex.main=0.9,xlab="Candidate interventions",ylab=paste("FWER in the same", Nsims, "simulations"),bty="n")

for(ii in 1:nrow(results)) lines(3:20+0.03*(ii-nrow(results)/2-0.5),power_fwer_results[["fwersims_2_g"]][ii,3:20],type="o",pch=20,col=cc[ii])

for(ii in 1:nrow(results)) { for(jj in 3:20) { pp = power_fwer_results[["fwersims_2_g"]][ii,jj] ; lines(rep(jj+0.03*(ii-nrow(results)/2-0.5),2),qbeta(c(0.025,0.975),Nsims*pp+0.5,Nsims*(1-pp)+0.5),col=cc[ii]) }}

lines(c(3,20),c(0.05,0.05),lty=2,col="lightgrey")

mtext("B.",side=3,line=-3,outer=T,at=0.5,cex=4,adj=0)

#####################################################

# For 4 allocations max (ssizes, then power and fwer)

#####################################################

#

if(is.null(power_fwer_results[["powersims_4_g"]])|is.null(power_fwer_results[["fwersims_4_g"]]))

{

treatments_eff = c(0.7,0.5) ; names(treatments_eff) = c("-1-","--")

results = matrix(rep(NA,120),nrow=6)

rownames(results) = c("Distributive","Full factorial*","Capped factorial","Parallel arms (pooled)","Parallel arms","Separate trials")

modalities_list = c("distributive","full factorial","capped factorial","parallel","single trial","single trial")

for(ii in 1:6) { for(jj in 5:20)

{

results[ii,jj] = integrated_calc(treatments_eff,NN=jj,allocs_integ=4,bonferroni_integ=jj,modality_integ=modalities_list[ii])$Size

}}

results["Parallel arms",] = results["Parallel arms",]/2*((1:ncol(results))+1) # Obtained from a H2H trial, /2 for arm size, multiply by Ntreatments+1

results["Separate trials",] = results["Separate trials",]*(1:ncol(results)) # Need to run several separate trials, not one

powersims = results*NA

fwersims = powersims

propalternative="t"

for(jj in 5:20) { for(ii in rownames(results))

{

sims = replicate(Nsims,simulate_one(NN=round(results[ii,jj]),Nmaxalloc=4,Ncandidates=jj,strategy=ii))

powersims[ii,jj] = mean(sims[1,]<0.05/jj)

fwersims[ii,jj] = mean(as.logical(colSums(sims[-1,]<0.05/jj)))

cat(ii,jj,powersims[ii,jj],fwersims[ii,jj],"\n")

}}

power_fwer_results[["powersims_4_g"]] = powersims

power_fwer_results[["fwersims_4_g"]] = fwersims

save(power_fwer_results,file="fwercheck analytic prop twosided.bak")

}

plot(0,0,xlim=c(3,20),ylim=c(0.85,1),col=0, main="Power (max 4 allocations, two-sided test)", cex.main=0.9,xlab="Candidate interventions",ylab=paste("Power in", Nsims, "simulations"),bty="n")

for(ii in 1:nrow(results)) lines(5:20+0.03*(ii-nrow(results)/2-0.5),power_fwer_results[["powersims_4_g"]][ii,5:20],type="o",pch=20,col=cc[ii])

for(ii in 1:nrow(results)) { for(jj in 5:20) { pp = power_fwer_results[["powersims_4_g"]][ii,jj] ; lines(rep(jj+0.03*(ii-nrow(results)/2-0.5),2),qbeta(c(0.025,0.975),Nsims*pp+0.5,Nsims*(1-pp)+0.5),col=cc[ii]) }}

lines(c(3,20),c(0.9,0.9),lty=2,col="lightgrey")

mtext("C.",side=3,line=-1*26-3,outer=T,at=0,cex=4,adj=0)

plot(0,0,xlim=c(3,20),ylim=c(0,0.15),col=0, main="Cognate FWER among null candidates", cex.main=0.9,xlab="Candidate interventions",ylab=paste("FWER in the same", Nsims, "simulations"),bty="n")

for(ii in 1:nrow(results)) lines(5:20+0.03*(ii-nrow(results)/2-0.5),power_fwer_results[["fwersims_4_g"]][ii,5:20],type="o",pch=20,col=cc[ii])

for(ii in 1:nrow(results)) { for(jj in 5:20) { pp = power_fwer_results[["fwersims_4_g"]][ii,jj] ; lines(rep(jj+0.03*(ii-nrow(results)/2-0.5),2),qbeta(c(0.025,0.975),Nsims*pp+0.5,Nsims*(1-pp)+0.5),col=cc[ii]) }}

lines(c(3,20),c(0.05,0.05),lty=2,col="lightgrey")

mtext("D.",side=3,line=-1*26-3,outer=T,at=0.5,cex=4,adj=0)

#####################################################

# For 8 allocations max (ssizes, then power and fwer)

#####################################################

#

if(is.null(power_fwer_results[["powersims_8_g"]])|is.null(power_fwer_results[["fwersims_8_g"]]))

{

treatments_eff = c(0.7,0.5) ; names(treatments_eff) = c("-1-","--")

results = matrix(rep(NA,120),nrow=6)

rownames(results) = c("Distributive","Full factorial*","Capped factorial","Parallel arms (pooled)","Parallel arms","Separate trials")

modalities_list = c("distributive","full factorial","capped factorial","parallel","single trial","single trial")

for(ii in 1:6) { for(jj in 9:20)

{

results[ii,jj] = integrated_calc(treatments_eff,NN=jj,allocs_integ=8,bonferroni_integ=jj,modality_integ=modalities_list[ii])$Size

}}

results["Parallel arms",] = results["Parallel arms",]/2*((1:ncol(results))+1) # Obtained from a H2H trial, /2 for arm size, multiply by Ntreatments+1

results["Separate trials",] = results["Separate trials",]*(1:ncol(results)) # Need to run several separate trials, not one

powersims = results*NA

fwersims = powersims

propalternative="t"

for(jj in 9:20) { for(ii in rownames(results))

{

sims = replicate(Nsims,simulate_one(NN=round(results[ii,jj]),Nmaxalloc=8,Ncandidates=jj,strategy=ii))

powersims[ii,jj] = mean(sims[1,]<0.05/jj)

fwersims[ii,jj] = mean(as.logical(colSums(sims[-1,]<0.05/jj)))

cat(ii,jj,powersims[ii,jj],fwersims[ii,jj],"\n")

}}

power_fwer_results[["powersims_8_g"]] = powersims

power_fwer_results[["fwersims_8_g"]] = fwersims

save(power_fwer_results,file="fwercheck analytic prop twosided.bak")

}

plot(0,0,xlim=c(3,20),ylim=c(0.85,1),col=0, main="Power (max 8 allocations, two-sided test)", cex.main=0.9,xlab="Candidate interventions",ylab=paste("Power in", Nsims, "simulations"),bty="n")

for(ii in 1:nrow(results)) lines(9:20+0.03*(ii-nrow(results)/2-0.5),power_fwer_results[["powersims_8_g"]][ii,9:20],type="o",pch=20,col=cc[ii])

for(ii in 1:nrow(results)) { for(jj in 9:20) { pp = power_fwer_results[["powersims_8_g"]][ii,jj] ; lines(rep(jj+0.03*(ii-nrow(results)/2-0.5),2),qbeta(c(0.025,0.975),Nsims*pp+0.5,Nsims*(1-pp)+0.5),col=cc[ii]) }}

lines(c(3,20),c(0.9,0.9),lty=2,col="lightgrey")

mtext("E.",side=3,line=-2*26-3,outer=T,at=0,cex=4,adj=0)

plot(0,0,xlim=c(3,20),ylim=c(0,0.15),col=0, main="Cognate FWER among null candidates", cex.main=0.9,xlab="Candidate interventions",ylab=paste("FWER in the same", Nsims, "simulations"),bty="n")

for(ii in 1:nrow(results)) lines(9:20+0.03*(ii-nrow(results)/2-0.5),power_fwer_results[["fwersims_8_g"]][ii,9:20],type="o",pch=20,col=cc[ii])

for(ii in 1:nrow(results)) { for(jj in 9:20) { pp = power_fwer_results[["fwersims_8_g"]][ii,jj] ; lines(rep(jj+0.03*(ii-nrow(results)/2-0.5),2),qbeta(c(0.025,0.975),Nsims*pp+0.5,Nsims*(1-pp)+0.5),col=cc[ii]) }}

lines(c(3,20),c(0.05,0.05),lty=2,col="lightgrey")

mtext("F.",side=3,line=-2*26-3,outer=T,at=0.5,cex=4,adj=0)

if(topdf) dev.off()

## #Generate data for Supplementary Figure S3

if(0){

source("./sup/3- Generate FWER for figure 3.r")

source("1- Clean setup.R")

source("4- Generate data without interaction terms.r")

if(!any(ls()=="topdf")) topdf = F

load("figdata_no_ixn.bak")

}

####################################################

# Declare some global environment variables

####################################################

#

assumptions = list(

c("--"=0.5,"-1-"=0.7),

c("--"=0.5,"-1-"=0.7,"-2-"=0.7,"-1-2-"=0.8448276),

c("--"=0.5,"-1-"=0.7,"-2-"=0.7,"-1-2-"=.99), # changed because convergence issues with 100%

c("--"=0.5,"-1-"=0.7,"-2-"=0.7,"-1-2-"=0.7),

c("--"=0.5,"-1-"=0.7,"-2-"=0.6,"-1-2-"=0.7) )

names(assumptions) = paste0("scenario",0:4)

situations = c("sizes_scenario0_assumed",

"sizes_scenario1_assumed",

"sizes_scenario2_assumed",

"sizes_scenario3_assumed",

"sizes_scenario4_assumed",

"power_scenario0_assumed_scenario0_true",

"power_scenario0_assumed_scenario1_true",

"power_scenario0_assumed_scenario2_true",

"power_scenario0_assumed_scenario3_true",

"power_scenario0_assumed_scenario4_true")

####################################################

# Define the function

####################################################

#

simulate_one = function(NN=1000,Nmaxalloc=2,Ncandidates=20, simtruth = assumptions[[1]], strategy=c("Distributive","Full factorial*")[1])

{

if(strategy=="Full factorial*") designmat = matrix(rbinom(NN*Ncandidates,1,0.5),nrow=NN)

if(strategy=="Capped factorial") designmat = t(replicate(NN,sample(c(rep(1,which(rmultinom(1,1,dbinom(0:Nmaxalloc,Ncandidates,.5))==1)-1),rep(0,Ncandidates))[1:Ncandidates]))) # the multinom part draws how many allocations in a truncated distribution (rather than defaulting to Nmaxalloc if above) ; both are possible but the paper was initially written such

if(strategy=="Distributive") designmat = t(replicate(NN,sample(c(rep(1,Nmaxalloc),rep(0,Ncandidates-Nmaxalloc)))))

if(grepl("Parallel arms",strategy)) designmat = t(replicate(NN,sample(c(1,rep(0,Ncandidates)))[1:Ncandidates]))

colnames(designmat) = paste0("-",1:Ncandidates,"-")

designmat=cbind(designmat,sapply(names(simtruth)[sapply(names(simtruth),function(xx){length(strsplit(xx,"-")[[1]])})>2],

function(xx){apply(matrix(as.logical(designmat[,paste("-",strsplit(xx,"-")[[1]][-1],"-",sep="")]),nrow=NN),1,all)})) # pick combinations whose presence needs be assessed & assess it

designmat = matrix(unlist(designmat),ncol=ncol(designmat),dimnames=dimnames(designmat))

simtruth = simtruth[order(names(simtruth))]

simtruth = simtruth[order(nchar(names(simtruth)))]

successprobs = rep(simtruth["--"],NN)

for(ii in names(simtruth)[names(simtruth)!="--"])

{

successprobs[as.logical(designmat[,ii])] = simtruth[ii] # set the outcome ; this can overwrite values several times, up to the largest specified combination (which is the right one in this specification)

}

colnames(designmat)[1:Ncandidates] = paste0("X",1:Ncandidates,"")

designmat = data.frame(designmat,ss=successprobs,yy=runif(NN)<successprobs)

pvals = summary(glm(as.formula(paste0("yy ~ ",paste(colnames(designmat)[1:Ncandidates],collapse=" + "))), family=binomial(logit),data=designmat))$coefficients[,"Pr(>|z|)"]

pvals_output = rep(1,Ncandidates)

names(pvals_output) = paste0("X",1:Ncandidates,"")

pvals_output[] = pvals[names(pvals_output)]

pvals_output[is.na(pvals_output)] = 1

pvals_output

}

simulate_one()

#############################################################

# Run the simulation (from existing data structure branching)

#############################################################

#

if(!any(ls()=="fwerdata_no_ixn")) fwerdata_no_ixn = figdata_no_ixn[grepl("power_",names(figdata_no_ixn))]

names(fwerdata_no_ixn) = gsub("power_","fwer_",names(fwerdata_no_ixn))

for(situation in rev(rev(situations)[1:5]))

{

expected_scenario = gsub("power_","",strsplit(situation,"_assumed_")[[1]][1])

true_scenario = gsub("_true","",strsplit(situation,"_assumed_")[[1]][2])

for(ii in 2:8) { cat("\n",expected_scenario,"expected,",true_scenario,"true,",ii,"allocations among",ii+1,"to","20 candidates","\n") ; for(jj in (ii+1):20)

{

if( !is.na(figdata_no_ixn[[situation]][["distributive"]][ii,jj])

&figdata_no_ixn[[situation]][["distributive"]][ii,jj]!=-1

&figdata_no_ixn[[situation]][["distributive"]][ii,jj]==fwerdata_no_ixn[[gsub("power_","fwer_",situation)]][["distributive"]][ii,jj]

)

{

fwer_output = replicate(1000,simulate_one(NN=figdata_no_ixn[[paste0("sizes_",expected_scenario,"_assumed")]][["distributive"]][ii,jj],Nmaxalloc=ii,Ncandidates=jj, simtruth = assumptions[[true_scenario]],strategy=c("Distributive")))

fwer_output = mean(as.logical(colSums(matrix(fwer_output[-(1:(1+(true_scenario!="scenario0"))),]<.05/jj,nrow=jj-(1+(true_scenario!="scenario0")) )) ))

fwerdata_no_ixn[[gsub("power_","fwer_",situation)]][["distributive"]][ii,jj] = fwer_output

cat(fwer_output,"")

}

if( !is.na(figdata_no_ixn[[situation]][["fullfactorial"]][jj])

&figdata_no_ixn[[situation]][["fullfactorial"]][jj]==fwerdata_no_ixn[[gsub("power_","fwer_",situation)]][["fullfactorial"]][jj]

&ii==2

)

{

fwer_output = replicate(1000,simulate_one(NN=figdata_no_ixn[[paste0("sizes_",expected_scenario,"_assumed")]][["fullfactorial"]][jj],Nmaxalloc=ii,Ncandidates=jj, simtruth = assumptions[[true_scenario]],strategy=c("Full factorial*")))

fwer_output = mean(as.logical(colSums(matrix(fwer_output[-(1:(1+(true_scenario!="scenario0"))),]<.05/jj,nrow=jj-(1+(true_scenario!="scenario0")) )) ))

fwerdata_no_ixn[[gsub("power_","fwer_",situation)]][["fullfactorial"]][jj] = fwer_output

cat("(f)",fwer_output," ",sep="")

}

} ; save(figdata_no_ixn,fwerdata_no_ixn,file="figdata_no_ixn.bak") }

}

## #Generate data for Supplementary Figure S4

if(0){

source("./sup/4- Generate FWER for figure 4.r")

source("1- Clean setup.R")

source("4- Generate data without interaction terms.r")

if(!any(ls()=="topdf")) topdf = F

load("figdata_no_ixn.bak")

}

for(scenarnum in 0:4) # create and populate the same data structure as the figdata tree

{

figdata_label = paste0("sizes_scenario",scenarnum,"_assumed")

fwerdata_label = paste0("fwer_scenario",scenarnum,"_assumed_scenario",scenarnum,"_true")

true_scenario = paste0("scenario",scenarnum)

if(!any(names(fwerdata_no_ixn)==fwerdata_label)) fwerdata_no_ixn[[fwerdata_label]] = figdata_no_ixn[[figdata_label]]

for(ii in 2:8) { cat("\n","scenario",scenarnum,"expected & true,",ii,"allocations among",ii+1,"to","20 candidates","\n") ; for(jj in (ii+1):20)

{

if( !is.na(figdata_no_ixn[[figdata_label]][["distributive"]][ii,jj])

&figdata_no_ixn[[figdata_label]][["distributive"]][ii,jj]!=-1

&figdata_no_ixn[[figdata_label]][["distributive"]][ii,jj]==fwerdata_no_ixn[[fwerdata_label]][["distributive"]][ii,jj]

)

{

fwer_output = replicate(1000,simulate_one(NN=figdata_no_ixn[[figdata_label]][["distributive"]][ii,jj],Nmaxalloc=ii,Ncandidates=jj, simtruth = assumptions[[true_scenario]],strategy=c("Distributive")))

fwer_output = mean(as.logical(colSums(matrix(fwer_output[-(1:(1+(true_scenario!="scenario0"))),]<.05/jj,nrow=jj-(1+(true_scenario!="scenario0")) )) ))

fwerdata_no_ixn[[fwerdata_label]][["distributive"]][ii,jj] = fwer_output

cat(fwer_output,"")

}

if( !is.na(figdata_no_ixn[[figdata_label]][["fullfactorial"]][jj])

&figdata_no_ixn[[figdata_label]][["fullfactorial"]][jj]==fwerdata_no_ixn[[fwerdata_label]][["fullfactorial"]][jj]

&ii==2

)

{

fwer_output = replicate(1000,simulate_one(NN=figdata_no_ixn[[figdata_label]][["fullfactorial"]][jj],Nmaxalloc=ii,Ncandidates=jj, simtruth = assumptions[[true_scenario]],strategy=c("Full factorial*")))

fwer_output = mean(as.logical(colSums(matrix(fwer_output[-(1:(1+(true_scenario!="scenario0"))),]<.05/jj,nrow=jj-(1+(true_scenario!="scenario0")) )) ))

fwerdata_no_ixn[[fwerdata_label]][["fullfactorial"]][jj] = fwer_output

cat("(f)",fwer_output," ",sep="")

}

save(figdata_no_ixn,fwerdata_no_ixn,file="figdata_no_ixn.bak")

}}

}

## #Draw Supplementary Figures S3 and S4

if(0){

source("./sup/5- Draw FWER figures.r")

}

load("figdata_no_ixn.bak")

if(!any(ls()=="topdf")) topdf = T

if(topdf) pdf("Figure - Simulated FWER for wrong assumption main effect model.pdf",width=7*2,height=7*2)

par(mfrow=c(2,2),cex=1.35,cex.lab=1,cex.axis=1)

if(!any(ls()=="Nsims")) Nsims = 1000

for(scenarnum in 1:4)

{

fwerdata_label = paste0("fwer_scenario0_assumed_scenario",scenarnum,"_true")

plot(0,0,xlim=c(3,20),ylim=c(0,.1),col=0, main=c("Both main effects 70%, logit−additivity","Both main effects 70%, synergy 99%","Both main effects 70%, no additivity","Occult main effect 60%, no additivity")[scenarnum], cex.main=0.9,xlab="Candidate interventions",ylab=paste("FWER in", Nsims, "simulations"),bty="n")

for(ii in 2:8)

{

results = fwerdata_no_ixn[[fwerdata_label]][["distributive"]]

color_palette = rgb(seq(.5,.5,l=7)-scenarnum*.1,seq(.6,.1,l=7)+scenarnum*.1,seq(.5,1,l=7))

lines((ii+2):20+0.03*(ii-nrow(results)/2-0.5),results[ii,(ii+2):20],type="o",pch=20,col=color_palette[ii-1])

for(jj in (ii+2):20)

{

pp = fwerdata_no_ixn[[fwerdata_label]][["distributive"]][ii,jj]

lines(rep(jj+0.03*(ii-5),2),qbeta(c(0.025,0.975),Nsims*pp+0.5,Nsims*(1-pp)+0.5),col=color_palette[ii-1])

}

}

lines(c(3,20),c(.05,.05),lty=2,col="lightgrey")

legend(20,.1,c("From 2 allocations...","... to 8 (per patient)"),pch=19,lwd=2,col=color_palette[c(1,7)],inset=0.01,bty="n",cex=0.9,xjust=1,yjust=1)

mtext(c("A.","B.","C.","D.")[scenarnum],side=3,line=c(-3,-3,-1*26-3,-1*26-3)[scenarnum],outer=T,at=c(0,.5,0,.5)[scenarnum],cex=4,adj=0)

}

dev.off()

if(topdf) pdf("Figure - Simulated FWER for correct assumptions main effect model.pdf",width=7*2,height=7*2)

par(mfrow=c(2,2),cex=1.35,cex.lab=1,cex.axis=1)

if(!any(ls()=="Nsims")) Nsims = 1000

for(scenarnum in 1:4)

{

fwerdata_label = paste0("fwer_scenario",scenarnum,"_assumed_scenario",scenarnum,"_true")

plot(0,0,xlim=c(3,20),ylim=c(0,.1),col=0, main=c("Both main effects 70%, logit−additivity","Both main effects 70%, synergy 99%","Both main effects 70%, no additivity","Occult main effect 60%, no additivity")[scenarnum], cex.main=0.9,xlab="Candidate interventions",ylab=paste("FWER in", Nsims, "simulations"),bty="n")

for(ii in 2:8)

{

results = fwerdata_no_ixn[[fwerdata_label]][["distributive"]]

color_palette = rgb(seq(.5,.5,l=7)-scenarnum*.1,seq(.1,.1,l=7),seq(.5,1,l=7))

lines((ii+2):20+0.03*(ii-nrow(results)/2-0.5),results[ii,(ii+2):20],type="o",pch=20,col=color_palette[ii-1])

for(jj in (ii+2):20)

{

pp = fwerdata_no_ixn[[fwerdata_label]][["distributive"]][ii,jj]

print(qbeta(c(0.025,0.975),Nsims*pp+0.5,Nsims*(1-pp)+0.5))

lines(rep(jj+0.03*(ii-5),2),qbeta(c(0.025,0.975),Nsims*pp+0.5,Nsims*(1-pp)+0.5),col=color_palette[ii-1])

}

}

lines(c(3,20),c(.05,.05),lty=2,col="lightgrey")

legend(20,.1,c("From 2 allocations...","... to 8 (per patient)"),pch=19,lwd=2,col=color_palette[c(1,7)],inset=0.01,bty="n",cex=0.9,xjust=1,yjust=1)

mtext(c("A.","B.","C.","D.")[scenarnum],side=3,line=c(-3,-3,-1*26-3,-1*26-3)[scenarnum],outer=T,at=c(0,.5,0,.5)[scenarnum],cex=4,adj=0)

}

dev.off()

## #Generate data for Supplementary Figure S5

if(0){

source("./sup/6- Generate data with pre-specified interaction.r")

}

if(any(list.files()=="figdata_one_ixn.bak")) load("figdata_one_ixn.bak")

situations = c("sizes_scenario0_assumed",

"sizes_scenario1_assumed",

"sizes_scenario2_assumed",

"sizes_scenario3_assumed",

"sizes_scenario4_assumed",

"power_scenario0_assumed_scenario0_true",

"power_scenario0_assumed_scenario1_true",

"power_scenario0_assumed_scenario2_true",

"power_scenario0_assumed_scenario3_true",

"power_scenario0_assumed_scenario4_true")

if(!any(ls()=="figdata_one_ixn"))

{

figdata_one_ixn = list(list(fullfactorial=rep(NA,20),distributive=matrix(NA,nrow=8,ncol=20)))[rep(1,length(situations))]

figdata_one_ixn[[1]][["distributive"]] = matrix(NA,nrow=19,ncol=20)

names(figdata_one_ixn)=situations

}

#if(!any(ls()=="figdata_one_ixn_sizes_wrong_assumption")) figdata_one_ixn_sizes_wrong_assumption = list(fullfactorial=rep(NA,20),distributive=matrix(rep(NA,8*20),nrow=8))

assumptions = list(

c("--"=0.5,"-1-"=0.7),

c("--"=0.5,"-1-"=0.7,"-2-"=0.7,"-1-2-"=0.8448276),

c("--"=0.5,"-1-"=0.7,"-2-"=0.7,"-1-2-"=.99), #changed cause 100% gives some crashing

c("--"=0.5,"-1-"=0.7,"-2-"=0.7,"-1-2-"=0.7),

c("--"=0.5,"-1-"=0.7,"-2-"=0.6,"-1-2-"=0.7) )

names(assumptions) = paste0("scenario",0:4)

for(scenario in names(assumptions))

{

situation = paste0("sizes_",scenario,"_assumed")

for(ii in 2:min(nrow(figdata_one_ixn[[situation]][["distributive"]]),ncol(figdata_one_ixn[[situation]][["distributive"]])-2)) { cat(scenario,",",ii,"allocations","\n") ; for(jj in (ii+2):20)

{

if(0&(scenario=="scenario2"|scenario=="scenario3"|scenario=="scenario4")&(jj-ii)==1&is.na(figdata_one_ixn[[situation]][["distributive"]][ii,jj])) { figdata_one_ixn[[situation]][["distributive"]][ii,jj] = -1 } # logistic cannot converge, too many parameters

if(is.na(figdata_one_ixn[[situation]][["distributive"]][ii,jj]))

{

cat("among ",jj,"\n")

figdata_one_ixn[[situation]][["distributive"]][ii,jj] = simulate_sampsize(odds=rep(1,jj),

allocs=ii,

nsims=5000,

true_effects = assumptions[[scenario]],

nominal_alphas=0.05,

modality=c("distributive","full factorial")[1],

analysis_algorithm = logreg_mains_ixn12)

}

if(is.na(figdata_one_ixn[[situation]][["fullfactorial"]][jj]))

{

cat("(factorial for ",jj,")","\n")

figdata_one_ixn[[situation]][["fullfactorial"]][jj] = simulate_sampsize(odds=rep(1,jj),

allocs=ii,

nsims=5000,

true_effects = assumptions[[scenario]],

nominal_alphas=0.05,

modality=c("distributive","full factorial")[2],

analysis_algorithm = logreg_mains_ixn12) }

save(figdata_one_ixn,file="figdata_one_ixn.bak")

}}

}

for(situation in rev(rev(situations)[1:5]))

{

expected_scenario = gsub("power_","",strsplit(situation,"_assumed_")[[1]][1])

true_scenario = gsub("_true","",strsplit(situation,"_assumed_")[[1]][2])

for(ii in 2:8) { for(jj in (ii+2):20)

{

cat(expected_scenario,"expected,",true_scenario,"true,",ii,"allocations among ",jj,"\n")

if(is.na(figdata_one_ixn[[situation]][["distributive"]][ii,jj])&!is.na(figdata_one_ixn[[paste0("sizes_",expected_scenario,"_assumed")]][["distributive"]][ii,jj])&figdata_one_ixn[[paste0("sizes_",expected_scenario,"_assumed")]][["distributive"]][ii,jj]!=-1)

{

power_output = simulate_power(odds=rep(1,jj),

allocs=ii,

sampsize=figdata_one_ixn[[paste0("sizes_",expected_scenario,"_assumed")]][["distributive"]][ii,jj],

nsims=5000,

true_effects = assumptions[[true_scenario]],

nominal_alphas=0.05,

modality=c("distributive","full factorial")[1],

analysis_algorithm = logreg_mains_ixn12)

print(power_output)

figdata_one_ixn[[situation]][["distributive"]][ii,jj] = power_output[1]

}

if(is.na(figdata_one_ixn[[situation]][["fullfactorial"]][jj]))

{

cat(expected_scenario,"expected,",true_scenario,"true,","factorial allocations among ",jj,"\n")

power_output = simulate_power(odds=rep(1,jj),

allocs=ii,

sampsize=figdata_one_ixn[[paste0("sizes_",expected_scenario,"_assumed")]][["fullfactorial"]][jj],

nsims=5000,

true_effects = assumptions[[true_scenario]],

nominal_alphas=0.05,

modality=c("distributive","full factorial")[2],

analysis_algorithm = logreg_mains_ixn12)

print(power_output)

figdata_one_ixn[[situation]][["fullfactorial"]][jj] = power_output[1]

}

save(figdata_one_ixn,file="figdata_one_ixn.bak")

}}

}

## #Draw Supplementary Figure S5

if(0){

source("./sup/7- Draw figures with pre-specified interaction.r")

}

load("figdata_one_ixn.bak")

situations = c("sizes_scenario0_assumed",

"sizes_scenario1_assumed",

"sizes_scenario2_assumed",

"sizes_scenario3_assumed",

"sizes_scenario4_assumed",

"power_scenario0_assumed_scenario0_true",

"power_scenario0_assumed_scenario1_true",

"power_scenario0_assumed_scenario2_true",

"power_scenario0_assumed_scenario3_true",

"power_scenario0_assumed_scenario4_true")

if(!any(ls()=="topdf")) topdf = T

if(topdf) pdf("Figure - ungated pre-specified.pdf",width=7*2,height=7*3)

par(mfrow=c(3,2),cex=1.35,cex.lab=1,cex.axis=1)

palette_anchors = rbind(c(0,0,0,0.5),c(1,.8,.8,.8))

cc = apply(color_spread(20,palette_anchors),2,function(xx){rgb(xx[1],xx[2],xx[3])})

plot(0,0,col=0,xlim=0:1,ylim=0:1*max(figdata_no_ixn$sizes_scenario0_assumed$distributive,na.rm=T), xlab="Proportion allocated per subject (k/K)",ylab="Trial size",main="Choosing the number of allocations", cex.main=0.9, bty="n")

mtext("A.",side=3,line=-3,outer=T,at=0,cex=4,adj=0)

for(jj in rev(c(5,10,15,20))) lines(0:1*.1+.45,rep(figdata_one_ixn$sizes_scenario0_assumed$fullfactorial[jj],2),type="l",pch=20,cex=0.5,col="grey",lwd=2)

for(jj in rev(c(5,10,15,20))) { kk = 2:(jj-2) ; lines(kk/jj,figdata_one_ixn$sizes_scenario0_assumed$distributive[kk,jj],type="o",pch=20,lwd=2,col=cc[jj-1]) }

legend(0.5,max(figdata_one_ixn$sizes_scenario0_assumed$distributive,na.rm=T),c("",5,10,15,20),pch=19,lwd=2,col=c(0,cc[4],cc[9],cc[15],cc[19]),inset=0.01,bty="n",cex=0.9,xjust=0.5,yjust=1)

legend(0.5,max(figdata_one_ixn$sizes_scenario0_assumed$distributive,na.rm=T),"K =",pch=19,lwd=2,col=c(0),inset=0.01,bty="n",cex=0.9,xjust=1,yjust=1)

palette_anchors = rbind(c(0,.3,.0,.9),c(1,.9,.7,.0))

cc = apply(color_spread(8,palette_anchors),2,function(xx){rgb(xx[1],xx[2],xx[3])})

plot(0,0,col=0,xlim=c(4,20),ylim=0:1*max(figdata_no_ixn$sizes_scenario0_assumed$distributive,na.rm=T),xlab="Number of candidates (K)",ylab="Trial size",main="Choosing the number of candidate interventions", cex.main=0.9, bty="n",xaxt="n")

axis(1, las=1, line=0.5,at=seq(4,20,4))

mtext("B.",side=3,line=-3,outer=T,at=0.5,cex=4,adj=0)

lines(4:20,figdata_one_ixn$sizes_scenario0_assumed$fullfactorial[4:20],type="l",pch=20,cex=0.5,col="grey",lwd=2)

for(ii in rev(c(2,4,6,8))) lines((ii+2):20,figdata_one_ixn$sizes_scenario0_assumed$distributive[ii,(ii+2):20],type="o",pch=20,lwd=2,col=cc[ii-1])

legend(10,max(figdata_one_ixn$sizes_scenario0_assumed$distributive,na.rm=T),c("",2,4,6,8),pch=19,lwd=2,col=c(0,cc[1],cc[3],cc[5],cc[7]),inset=0.01,bty="n",cex=0.9,xjust=0.5,yjust=1)

legend(10,max(figdata_one_ixn$sizes_scenario0_assumed$distributive,na.rm=T),"k =",pch=19,lwd=2,col=c(0),inset=0.01,bty="n",cex=0.9,xjust=1,yjust=1)

if(!topdf) dev.new()

if(!topdf) par(mfrow=c(2,2),cex=1.35,cex.lab=1,cex.axis=1)

scenario_names = c("Both main effects 70%, logit-additivity","Both main effects 70%, synergy 99%","Both main effects 70%, no additivity","Occult main effect 60%, no additivity")

for(kk in 1:4)

{

situation = rev(rev(situations)[1:4])[kk]

palette_anchors = rbind(c(0.0,0.0+0*kk,0.0+0*kk,0.5-0*kk),c(1,0.+0*kk,0.8-0*kk,0.8+0*kk))

cc = apply(color_spread(8,palette_anchors),2,function(xx){rgb(xx[1],xx[2],xx[3])})

plot(0,0,col=0,xlim=c(4,20),ylim=0:1,xlab="Number of candidates",ylab="Statistical power",main=scenario_names[kk], cex.main=0.9, bty="n",xaxt="n")

axis(1, las=1, line=0.5,at=seq(4,20,4))

mtext(c("C.","D.","E.","F.")[kk],side=3,line=-(1+(kk-1)%/%2)*26-3,outer=T,at=c(0,.5,0,.5)[kk],cex=4,adj=0)

lines(4:20,figdata_one_ixn[[situation]][["fullfactorial"]][4:20],type="l",pch=20,cex=0.5,col="grey",lwd=2)

for(ii in 2:8) lines((ii+2):20,figdata_one_ixn[[situation]][["distributive"]][ii,(ii+2):20],type="o",pch=20,lwd=2,col=cc[ii-1])

if(kk==1) legend(20,0,c("From 2 allocations... ","... to 8 (per patient)"),pch=19,lwd=2,col=c(cc[1],cc[7]),inset=0.01,bty="n",cex=0.9,xjust=1,yjust=0,title="Color gradient",title.adj=0)

}

if(topdf) dev.off() else dev.new()

if(topdf) pdf("Figure - ungated pre-specified sampsize shifts.pdf",width=7*2,height=7*2)

par(mfrow=c(2,2),cex=1.35,cex.lab=1,cex.axis=1)

scenario_names = c("Both main effects 70%, logit-additivity","Both main effects 70%, synergy 99%","Both main effects 70%, no additivity","Occult main effect 60%, no additivity")

for(kk in 1:4)

{

situation = situations[1+kk]

palette_anchors = rbind(c(0.0,0.8+0*kk,0.5+0*kk,0.0-0*kk),c(1,0.8+0*kk,0.0-0*kk,0.0+0*kk))

cc = apply(color_spread(8,palette_anchors),2,function(xx){rgb(xx[1],xx[2],xx[3])})

plot(0,0,col=0,xlim=c(4,20),ylim=c(-2,4),xlab="Number of candidates",ylab="Sample size fold-change",main=scenario_names[kk], cex.main=0.9, bty="n",yaxt="n",xaxt="n")

axis(1, las=1, line=0.5,at=seq(4,20,4))

mtext(c("A.","B.","C.","D.")[kk],side=3,line=-(0+(kk-1)%/%2)*26-3,outer=T,at=c(0,.5,0,.5)[kk],cex=4,adj=0)

axis(2,at=-2:4,labels=c("¼","½",1,2,4,8,16))

lines(log(figdata_one_ixn[[situation]][["fullfactorial"]]/figdata_one_ixn[["sizes_scenario0_assumed"]][["fullfactorial"]])/log(2),type="l",pch=20,cex=0.5,col="grey",lwd=2)

for(ii in 2:8)

{

figdata_one_ixn[[situation]][["distributive"]][ii,!is.na(figdata_one_ixn[[situation]][["distributive"]][ii,]) & figdata_one_ixn[[situation]][["distributive"]][ii,] == -1] = NA

lines((ii+2):20,log(figdata_one_ixn[[situation]][["distributive"]][ii,(ii+2):20]/figdata_one_ixn[["sizes_scenario0_assumed"]][["distributive"]][ii,(ii+2):20])/log(2),type="o",pch=20,lwd=2,col=cc[ii-1])

}

if(kk==1) legend(20,4,c("From 2 allocations... ","... to 8 (per patient)"),pch=19,lwd=2,col=c(cc[1],cc[7]),inset=0.01,bty="n",cex=0.9,xjust=1,yjust=1,title="Color gradient",title.adj=0)

}

if(topdf) dev.off()

## #Generate data for Supplementary Figure S6

if(0)

{

source("1- Clean setup.r")

source("./sup/8- Generate data with gated pre-specified interaction.r")

}

if(any(list.files()=="figdata_ixn_12desc05.bak")) load("figdata_ixn_12desc05.bak")

#if(any(list.files()=="figdata_ixn_12desc05.bak")&!any(ls()=="figdata_ixn_12desc05")) load("figdata_ixn_12desc05.bak")

situations = c("sizes_scenario0_assumed",

"sizes_scenario1_assumed",

"sizes_scenario2_assumed",

"sizes_scenario3_assumed",

"sizes_scenario4_assumed",

"power_scenario0_assumed_scenario0_true",

"power_scenario0_assumed_scenario1_true",

"power_scenario0_assumed_scenario2_true",

"power_scenario0_assumed_scenario3_true",

"power_scenario0_assumed_scenario4_true")

if(!any(ls()=="figdata_ixn_12desc05"))

{

figdata_ixn_12desc05 = list(list(fullfactorial=rep(NA,20),distributive=matrix(NA,nrow=8,ncol=20)))[rep(1,length(situations))]

figdata_ixn_12desc05[[1]][["distributive"]] = matrix(NA,nrow=19,ncol=20)

names(figdata_ixn_12desc05)=situations

}

#if(!any(ls()=="figdata_ixn_12desc05_sizes_wrong_assumption")) figdata_ixn_12desc05_sizes_wrong_assumption = list(fullfactorial=rep(NA,20),distributive=matrix(rep(NA,8*20),nrow=8))

assumptions = list(

c("--"=0.5,"-1-"=0.7),

c("--"=0.5,"-1-"=0.7,"-2-"=0.7,"-1-2-"=0.8448276),

c("--"=0.5,"-1-"=0.7,"-2-"=0.7,"-1-2-"=0.99),

c("--"=0.5,"-1-"=0.7,"-2-"=0.7,"-1-2-"=0.7),

c("--"=0.5,"-1-"=0.7,"-2-"=0.6,"-1-2-"=0.7) )

names(assumptions) = paste0("scenario",0:4)

for(scenario in names(assumptions))

{

situation = paste0("sizes_",scenario,"_assumed")

for(ii in 2:min(ncol(figdata_ixn_12desc05[[situation]][["distributive"]])-2,nrow(figdata_ixn_12desc05[[situation]][["distributive"]]))) { cat(scenario,",",ii,"allocations","\n") ; for(jj in (ii+2):20)

{

if(0&(scenario=="scenario2"|scenario=="scenario3"|scenario=="scenario4")&(jj-ii)==1&is.na(figdata_ixn_12desc05[[situation]][["distributive"]][ii,jj])) { figdata_ixn_12desc05[[situation]][["distributive"]][ii,jj] = -1 } # logistic cannot converge, too many parameters

if(is.na(figdata_ixn_12desc05[[situation]][["distributive"]][ii,jj]))

{

cat("among",jj, " ")

figdata_ixn_12desc05[[situation]][["distributive"]][ii,jj] = simulate_sampsize(odds=rep(1,jj),

allocs=ii,

nsims=5000,

true_effects = assumptions[[scenario]],

nominal_alphas=0.05,

modality=c("distributive","full factorial")[1],

analysis_algorithm = logreg_mains_ixn12desc05)

}

if(is.na(figdata_ixn_12desc05[[situation]][["fullfactorial"]][jj]))

{

figdata_ixn_12desc05[[situation]][["fullfactorial"]][jj] = simulate_sampsize(odds=rep(1,jj),

allocs=ii,

nsims=5000,

true_effects = assumptions[[scenario]],

nominal_alphas=0.05,

modality=c("distributive","full factorial")[2],

analysis_algorithm = logreg_mains_ixn12desc05)

}

save(figdata_ixn_12desc05,file="figdata_ixn_12desc05.bak")

}}

}

for(situation in rev(rev(situations)[1:5]))

{

expected_scenario = gsub("power_","",strsplit(situation,"_assumed_")[[1]][1])

true_scenario = gsub("_true","",strsplit(situation,"_assumed_")[[1]][2])

for(ii in 2:8) { cat(expected_scenario,"expected,",true_scenario,"true,",ii,"allocations","\n") ; for(jj in (ii+1):20)

{

if(is.na(figdata_ixn_12desc05[[situation]][["distributive"]][ii,jj])&!is.na(figdata_ixn_12desc05[[paste0("sizes_",expected_scenario,"_assumed")]][["distributive"]][ii,jj])&figdata_ixn_12desc05[[paste0("sizes_",expected_scenario,"_assumed")]][["distributive"]][ii,jj]!=-1)

{

cat("among ",jj)

power_output = simulate_power(odds=rep(1,jj),

allocs=ii,

sampsize=figdata_ixn_12desc05[[paste0("sizes_",expected_scenario,"_assumed")]][["distributive"]][ii,jj],

nsims=5000,

true_effects = assumptions[[true_scenario]],

nominal_alphas=0.05,

modality=c("distributive","full factorial")[1],

analysis_algorithm = logreg_mains_ixn12desc05)

print(power_output)

figdata_ixn_12desc05[[situation]][["distributive"]][ii,jj] = power_output[1]

}

if(is.na(figdata_ixn_12desc05[[situation]][["fullfactorial"]][jj]))

{

cat("among ",jj,"(factorial)")

power_output = simulate_power(odds=rep(1,jj),

allocs=ii,

sampsize=figdata_ixn_12desc05[[paste0("sizes_",expected_scenario,"_assumed")]][["fullfactorial"]][jj],

nsims=5000,

true_effects = assumptions[[true_scenario]],

nominal_alphas=0.05,

modality=c("distributive","full factorial")[2],

analysis_algorithm = logreg_mains_ixn12desc05)

print(power_output)

figdata_ixn_12desc05[[situation]][["fullfactorial"]][jj] = power_output[1]

}

save(figdata_ixn_12desc05,file="figdata_ixn_12desc05.bak")

}}

}

## #Draw Supplementary Figure S6

if(0)

{

source("1- Clean setup.r")

source("./sup/9- Draw figures with gated pre-specified interaction.r")

}

load("figdata_ixn_12desc05.bak")

situations = c("sizes_scenario0_assumed",

"sizes_scenario1_assumed",

"sizes_scenario2_assumed",

"sizes_scenario3_assumed",

"sizes_scenario4_assumed",

"power_scenario0_assumed_scenario0_true",

"power_scenario0_assumed_scenario1_true",

"power_scenario0_assumed_scenario2_true",

"power_scenario0_assumed_scenario3_true",

"power_scenario0_assumed_scenario4_true")

if(!any(ls()=="topdf")) topdf = T

if(topdf) pdf("Figure - distributive gated pre-specified.pdf",width=7*2,height=7*3)

par(mfrow=c(3,2),cex=1.35,cex.lab=1,cex.axis=1)

palette_anchors = rbind(c(0,0,0,0.5),c(1,.8,.8,.8))

cc = apply(color_spread(20,palette_anchors),2,function(xx){rgb(xx[1],xx[2],xx[3])})

plot(0,0,col=0,xlim=0:1,ylim=0:1*max(figdata_no_ixn$sizes_scenario0_assumed$distributive,na.rm=T), xlab="Proportion allocated per subject (k/K)",ylab="Trial size",main="Choosing the number of allocations", cex.main=0.9, bty="n")

mtext("A.",side=3,line=-3,outer=T,at=0,cex=4,adj=0)

for(jj in rev(c(5,10,15,20))) lines(0:1*.1+.45,rep(figdata_ixn_12desc05$sizes_scenario0_assumed$fullfactorial[jj],2),type="l",pch=20,cex=0.5,col="grey",lwd=2)

for(jj in rev(c(5,10,15,20))) { kk = 2:(jj-2) ; lines(kk/jj,figdata_ixn_12desc05$sizes_scenario0_assumed$distributive[kk,jj],type="o",pch=20,lwd=2,col=cc[jj-1]) }

legend(0.5,max(figdata_ixn_12desc05$sizes_scenario0_assumed$distributive,na.rm=T),c("",5,10,15,20),pch=19,lwd=2,col=c(0,cc[4],cc[9],cc[15],cc[19]),inset=0.01,bty="n",cex=0.9,xjust=0.5,yjust=1)

legend(0.5,max(figdata_ixn_12desc05$sizes_scenario0_assumed$distributive,na.rm=T),"K =",pch=19,lwd=2,col=c(0),inset=0.01,bty="n",cex=0.9,xjust=1,yjust=1)

palette_anchors = rbind(c(0,.3,.0,.9),c(1,.9,.7,.0))

cc = apply(color_spread(8,palette_anchors),2,function(xx){rgb(xx[1],xx[2],xx[3])})

plot(0,0,col=0,xlim=c(4,20),ylim=0:1*max(figdata_no_ixn$sizes_scenario0_assumed$distributive,na.rm=T),xlab="Number of candidates (K)",ylab="Trial size",main="Choosing the number of candidate interventions", cex.main=0.9, bty="n",xaxt="n")

axis(1, las=1, line=0.5,at=seq(4,20,4))

mtext("B.",side=3,line=-3,outer=T,at=0.5,cex=4,adj=0)

lines(4:20,figdata_ixn_12desc05$sizes_scenario0_assumed$fullfactorial[4:20],type="l",pch=20,cex=0.5,col="grey",lwd=2)

for(ii in rev(c(2,4,6,8))) lines((ii+2):20,figdata_ixn_12desc05$sizes_scenario0_assumed$distributive[ii,(ii+2):20],type="o",pch=20,lwd=2,col=cc[ii-1])

legend(10,max(figdata_ixn_12desc05$sizes_scenario0_assumed$distributive,na.rm=T),c("",2,4,6,8),pch=19,lwd=2,col=c(0,cc[1],cc[3],cc[5],cc[7]),inset=0.01,bty="n",cex=0.9,xjust=0.5,yjust=1)

legend(10,max(figdata_ixn_12desc05$sizes_scenario0_assumed$distributive,na.rm=T),"k =",pch=19,lwd=2,col=c(0),inset=0.01,bty="n",cex=0.9,xjust=1,yjust=1)

if(!topdf) dev.new()

if(!topdf) par(mfrow=c(2,2),cex=1.35,cex.lab=1,cex.axis=1)

scenario_names = c("Both main effects 70%, logit-additivity","Both main effects 70%, synergy 99%","Both main effects 70%, no additivity","Occult main effect 60%, no additivity")

for(kk in 1:4)

{

situation = rev(rev(situations)[1:4])[kk]

palette_anchors = rbind(c(0.0,0.0+0*kk,0.0+0*kk,0.5-0*kk),c(1,0.+0*kk,0.8-0*kk,0.8+0*kk))

cc = apply(color_spread(8,palette_anchors),2,function(xx){rgb(xx[1],xx[2],xx[3])})

plot(0,0,col=0,xlim=c(4,20),ylim=0:1,xlab="Number of candidates",ylab="Statistical power",main=scenario_names[kk], cex.main=0.9, bty="n",xaxt="n")

axis(1, las=1, line=0.5,at=seq(4,20,4))

mtext(c("C.","D.","E.","F.")[kk],side=3,line=-(1+(kk-1)%/%2)*26-3,outer=T,at=c(0,.5,0,.5)[kk],cex=4,adj=0)

lines(4:20,figdata_ixn_12desc05[[situation]][["fullfactorial"]][4:20],type="l",pch=20,cex=0.5,col="grey",lwd=2)

for(ii in 2:8) lines((ii+2):20,figdata_ixn_12desc05[[situation]][["distributive"]][ii,(ii+2):20],type="o",pch=20,lwd=2,col=cc[ii-1])

if(kk==1) legend(20,0,c("From 2 allocations... ","... to 8 (per patient)"),pch=19,lwd=2,col=c(cc[1],cc[7]),inset=0.01,bty="n",cex=0.9,xjust=1,yjust=0,title="Color gradient",title.adj=0)

}

if(topdf) dev.off() else dev.new()

if(topdf) pdf("Figure - distributive gated pre-specified sampsize shifts.pdf",width=7*2,height=7*2)

par(mfrow=c(2,2),cex=1.35,cex.lab=1,cex.axis=1)

scenario_names = c("Both main effects 70%, logit-additivity","Both main effects 70%, synergy 100%","Both main effects 70%, no additivity","Occult main effect 60%, no additivity")

for(kk in 1:4)

{

situation = situations[1+kk]

palette_anchors = rbind(c(0.0,0.8+0*kk,0.5+0*kk,0.0-0*kk),c(1,0.8+0*kk,0.0-0*kk,0.0+0*kk))

cc = apply(color_spread(8,palette_anchors),2,function(xx){rgb(xx[1],xx[2],xx[3])})

plot(0,0,col=0,xlim=c(4,20),ylim=c(-2,4),xlab="Number of candidates",ylab="Sample size fold-change",main=scenario_names[kk], cex.main=0.9, bty="n",yaxt="n",xaxt="n")

axis(1, las=1, line=0.5,at=seq(4,20,4))

mtext(c("A.","B.","C.","D.")[kk],side=3,line=-(0+(kk-1)%/%2)*26-3,outer=T,at=c(0,.5,0,.5)[kk],cex=4,adj=0)

axis(2,at=-2:4,labels=c("¼","½",1,2,4,8,16))

lines(log(figdata_ixn_12desc05[[situation]][["fullfactorial"]]/figdata_ixn_12desc05[["sizes_scenario0_assumed"]][["fullfactorial"]])/log(2),type="l",pch=20,cex=0.5,col="grey",lwd=2)

for(ii in 2:8)

{

figdata_ixn_12desc05[[situation]][["distributive"]][ii,!is.na(figdata_ixn_12desc05[[situation]][["distributive"]][ii,]) & figdata_ixn_12desc05[[situation]][["distributive"]][ii,] == -1] = NA

lines((ii+2):20,log(figdata_ixn_12desc05[[situation]][["distributive"]][ii,(ii+2):20]/figdata_ixn_12desc05[["sizes_scenario0_assumed"]][["distributive"]][ii,(ii+2):20])/log(2),type="o",pch=20,lwd=2,col=cc[ii-1])

}

if(kk==1) legend(20,4,c("From 2 allocations... ","... to 8 (per patient)"),pch=19,lwd=2,col=c(cc[1],cc[7]),inset=0.01,bty="n",cex=0.9,xjust=1,yjust=1,title="Color gradient",title.adj=0)

}

if(topdf) dev.off()

## #Generate data for Supplementary Figure S7

if(0){

source("./sup/13- Generate fractional factorial data.r")

}

source("1- Clean setup.R")

require("FrF2")

if(!any(ls()=="topdf")) topdf = T

#Fractional factorial table, default settings

if(!any(list.files()=="fractional_tables.bak"))

{

require(FrF2)

fractional_tables = sapply(1:20,function(xx)

{

if(xx<4)

{ aa=NULL } else {

aa = FrF2(2^ceiling(0.6*xx), xx, default.levels = c("0", "1"))

aa = data.frame(aa[order(apply(aa,1,function(xx) paste(xx,collapse=""))),])

colnames(aa) = paste("X",1:xx,sep="")

}

aa

})

save(fractional_tables,file="fractional_tables.bak")

} else { load(file="fractional_tables.bak") }

####################################################

# Define the function

####################################################

#

assumptions = list(

c("--"=0.5,"-1-"=0.7),

c("--"=0.5,"-1-"=0.7,"-2-"=0.7,"-1-2-"=0.8448276),

c("--"=0.5,"-1-"=0.7,"-2-"=0.7,"-1-2-"=.99), # changed because convergence issues with 100%

c("--"=0.5,"-1-"=0.7,"-2-"=0.7,"-1-2-"=0.7),

c("--"=0.5,"-1-"=0.7,"-2-"=0.6,"-1-2-"=0.7) )

names(assumptions) = paste0("scenario",0:4)

simulate_one_fracfac = function( NN=1000

,true_effects = c("--"=0.5,"-1-"=0.7,"-1-2-"=0.99)

,Ncandidates=20

,analysis_algorithm = logreg_mains )

{

true_effects=true_effects[order(nchar(names(true_effects)),decreasing=F)]

designmat = data.frame(lapply(fractional_tables[[Ncandidates]][sample(1:nrow(fractional_tables[[Ncandidates]]),NN,replace=T),],as.numeric))-1

yy = rep(true_effects["--"],NN)

for(ii in names(true_effects)[names(true_effects)!="--"])

{

ss = apply(data.frame(lapply(strsplit(gsub("-","",ii),"")[[1]],function(xx) designmat[,paste("X",xx,sep="")]==1 )),1,all)

yy[ss] = true_effects[ii] # set the outcome ; this can overwrite values several times, up to the largest specified combination (which is the right one in this specification)

}

yy = runif(NN)<yy

names(yy) = rownames(designmat)

analysis_algorithm(cbind(yy,designmat))

}

simulate_sampsize_fracfac = function(odds=rep(1,10), allocs=4, tar_power=0.9, nsims=5000, true_effects = c("-1-2-3-"=0.9,"-1-"=0.7,"--"=0.5), nominal_alphas = 0.05, bonferroni = NA, modality=c("distributive","full factorial")[1], analysis_algorithm = logreg_mains, verbose=F, proportion_true_control=0)

{

if(is.na(bonferroni)) { bonferroni = rep(length(odds),length(odds)) }

# here is where it starts being different

# we are going to compute different sample sizes only a few times and then fit a logistic regression

# this will enable picking a new range of values, and we keep doing this until our estimate for the sample size giving desired poser converges

tarNN = round(exp(seq(log(50),log(10000),length.out=48)))

latest_estimate = sqrt(10000)

latest_beta=.1

trials = matrix(c(1,sqrt(1000000),0,1),nrow=2)

colnames(trials) = c("srsampsize","nullreject")

se = NA

intercept_target = qnorm(tar_power)

trimmed_initiation = FALSE

#clusterExport(workingcluster,c("drawing_table","remaining_draws","modality","allocs","odds","analysis_algorithm"), envir=environment() )

while(nrow(trials)<nsims) #abs(latest_estimate^2-older_estimate^2)/latest_estimate^2>0.01|is.na(se)|se>.3)

{

cat("-")

simulations = sapply(tarNN,function(xx) simulate_one_fracfac( NN=xx

,true_effects = true_effects

,Ncandidates = length(odds)

,analysis_algorithm = analysis_algorithm

)[1]<nominal_alphas/bonferroni[1] )

if(nrow(trials)<200&nrow(trials)>50) cat(round(median(tarNN)))

if(nrow(trials)>200&!trimmed_initiation) { trials=trials[-(1:100),] ; trimmed_initiation = TRUE } # this prevents getting stuck because of an unlucky initiation

plot(trials[,1]^2,xlim=c(0,nsims*1.1),ylim=quantile(trials[,1],c(.01,.99))^2,main=c("Trace of sample size exploration",paste0("Current estimate ",round(latest_estimate^2),", slope ",round(latest_beta,3))),xlab="Simulation#",ylab="Sample size",type="p",col=2+trials[,2],pch=3,cex=.25+.25*(1-trials[,2]))

trials = rbind(trials,cbind(srsampsize=sqrt(tarNN),simulations))

loglik = function(xx) { sum(log( (1-trials[,"nullreject"]) + (trials[,"nullreject"]-.5)*2*pnorm(intercept_target+xx["srbeta"]^2*(trials[,"srsampsize"]-xx["X_target"])))) }

if(verbose) { print(trials[nrow(trials)-2:0,]) ; cat("\n") ; print(c("beta"=latest_beta, "X_target"=latest_estimate)) }

initial_value = loglik(c("srbeta"=.3, "X_target"=mean(trials[,"srsampsize"])))

if(initial_value!=Inf&initial_value!=-Inf&!is.na(initial_value))

{

try(mle <- optim(c("srbeta"=.3, "X_target"=mean(trials[,"srsampsize"])), fn=loglik, hessian = TRUE, control=list(fnscale=-1)), silent = TRUE)

latest_beta = unname(mle$par["srbeta"]^2)

latest_estimate = unname(mle$par["X_target"])

if(det(mle$hessian)!=0) { se = sqrt(diag(solve(-mle$hessian)))["X_target"] } else {se = 10}

if(is.na(se)){se=NULL}

tarNN = pmax(7,latest_estimate+c(-1,1)*min(0,se)) # span a window of +- 1 SE except if SE too large, minimum trial size 49

tarNN = round(seq(tarNN[1],tarNN[2],length.out=max(50,round(nrow(trials)/10)))^2) # in increments of 10% of the number of existing data points, evenly spread

} else {

tarNN = round(seq(quantile(trials[,1],.01),quantile(trials[,1],.99),length.out=max(50,round(nrow(trials)/10)))^2)

latest_estimate = 30

latest_beta=0.3

cat("LLfail")

} # the else loop may rarely be needed to unstick the algorithm

if(verbose) { cat("\n") ; print(tarNN); cat("\n") ; print(se) ; cat("\n") }

}

cat("sample size found : ", round(latest_estimate^2), " with ",nrow(trials), " simulated trials", "\n")

round(latest_estimate^2)

}

if(!any(ls()=="figdata_fracfac"))

{

if(!any(list.files()=="figdata_fracfac.bak"))

{

figdata_fracfac = lapply(c("no_ixn","one_ixn","ixn_12desc05"), function(xx) lapply(names(figdata_no_ixn),function(xx) rep(NA,20) ) )

names(figdata_fracfac) = c("no_ixn","one_ixn","ixn_12desc05")

names(figdata_fracfac[["no_ixn"]]) = names(figdata_no_ixn)

names(figdata_fracfac[["one_ixn"]]) = names(figdata_no_ixn)

names(figdata_fracfac[["ixn_12desc05"]]) = names(figdata_no_ixn)

} else { load(file="figdata_fracfac.bak") }

}

for(ii in names(figdata_fracfac))

{

cat(ii,"\n")

for(jj in names(figdata_fracfac[[ii]]))

{

cat(jj,"\n")

for(kk in seq(4,20,by=2))

{

if(is.na(figdata_fracfac[[ii]][[jj]][kk])) { cat("-") } else { cat("+") }

}

cat("\n")

}

}

#Sample sizes

for(scenario in names(assumptions))

{

situation = paste0("sizes_",scenario,"_assumed")

cat(scenario,", fractional with 40% interventions (rounded down) aliased","\n")

for(ii in c("no_ixn","one_ixn","ixn_12desc05"))

{ cat(ii,"analysis algorithm","\n")

for(jj in seq(4,20,by=2))

{

if(is.na(figdata_fracfac[[ii]][[situation]][jj]))

{

cat("computing",jj,"interventions","\n")

figdata_fracfac[[ii]][[situation]][jj] = simulate_sampsize_fracfac( odds=rep(1,jj)

,nsims=5000

,true_effects = assumptions[[scenario]]

,nominal_alphas=0.05

,analysis_algorithm = list( no_ixn = logreg_mains

,one_ixn = logreg_mains_ixn12

,ixn_12desc05 = logreg_mains_ixn12desc05

)[[ii]]

)

}

save(figdata_fracfac,file="figdata_fracfac.bak")

}

}

}

## #Draw Supplementary Figure S7

if(!any(ls()=="figdata_one_ixn")) load(file="figdata_one_ixn.bak")

if(!any(ls()=="figdata_no_ixn")) load(file="figdata_no_ixn.bak")

if(!any(ls()=="figdata_fracfac")) load(file="figdata_fracfac.bak")

if(!any(ls()=="topdf")) topdf = T

if(topdf)

{

pdf("Figure - frac vs full.pdf",width=7*2,height=7*1)

par(mfrow=c(1,2),cex=1.3,cex.lab=1.3,cex.axis=1.3)

}

par(mar=c(4,4,4,0))

plot(0,0,xlim=c(4,20),ylim=1*c(0,max(unlist(figdata_fracfac$no_ixn[1:5]),na.rm=T)),col=0,xlab="Number of candidates",ylab="Sample size",bty="n",yaxt="n",xaxt="n",main="No interaction term")

axis(1,at=seq(4,20,b=4))

axis(2,at=seq(0,1600,b=400))

mtext("A.",side=3,line=-3,outer=T,at=0,cex=4,adj=0)

colorset = c("black",rgb(.0,.99,.5),rgb(.0,.6,.4),rgb(.5,.7,.9),rgb(.0,.2,.6))

scenarioset = paste0("sizes_scenario",0:4,"_assumed")

for(ii in 1:5) { points(seq(4,20,b=2),figdata_fracfac$no_ixn[[scenarioset[ii]]][seq(4,20,b=2)],col=colorset[ii],lwd=3,pch=4,cex=1.5) ; lines(seq(4,20,b=2),figdata_no_ixn[[scenarioset[ii]]]$fullfactorial[seq(4,20,b=2)],col=colorset[ii],lwd=3,type="p",pch=1) }

#legend("topleft",legend=c("None","Additive","Synergistic","Non-additive","Semi-non-additive"),col=colorset,pch=20,lty=1,bty="n")

#legend("topright",legend=c("Fractional (40% aliased)","Full"),col="grey",pch=c(20,NA),lty=c(0,1),bty="n")

plot(0,0,xlim=c(4,20),ylim=1*c(0,max(unlist(figdata_fracfac$no_ixn[1:5]),na.rm=T)),col=0,xlab="Number of candidates",ylab="Sample size",bty="n",yaxt="n",xaxt="n",main="Systematic interaction term")

axis(1,at=seq(4,20,b=4))

axis(2,at=seq(0,1600,b=400))

mtext("B.",side=3,line=-3,outer=T,at=0.5,cex=4,adj=0)

for(ii in 1:5) { points(seq(4,20,b=2),figdata_fracfac$one_ixn[[scenarioset[ii]]][seq(4,20,b=2)],col=colorset[ii],lwd=3,pch=4,cex=1.5) ; lines(seq(4,20,b=2),figdata_one_ixn[[scenarioset[ii]]]$fullfactorial[seq(4,20,b=2)],col=colorset[ii],lwd=3,type="p",pch=1) }

legend("topright",legend=c("None","Additive","Synergistic","Non-additive","Semi-non-additive","Fractional (40% aliased)","Full factorial"),col=c(colorset,"grey","grey"),pch=c(rep(NA,5),4,1),pt.cex=1.5,lty=c(rep(1,5),NA,NA),lwd=c(rep(15,5),3,3),bty="n")

if(topdf) dev.off()

# #Code for quick calculations used in reviewer response 1

simulate_several_trials = function(NNs=rep(100,100))

{

tables = lapply(NNs,simulate_one_trial)

unlist(lapply(tables,function(XX){XX["x1","Pr(>|t|)"]}))

}

simulate_sampsize = function(tar_power=0.9, nsims=5000, verbose=F)

{

trimmed_initiation = FALSE

tarNN = round(exp(seq(log(50),log(10000),length.out=48)))

older_estimate = 0

latest_estimate = sqrt(10000)

latest_beta=.1

trials = matrix(c(1,sqrt(1000000),0,1),nrow=2)

colnames(trials) = c("srsampsize","nullreject")

se = NA

intercept_target = qnorm(tar_power)

while(nrow(trials)<nsims)

{

cat("-")

simulations = simulate_several_trials(tarNN)<0.05

if(nrow(trials)<150) cat(round(median(tarNN)))

if(nrow(trials)>150&!trimmed_initiation) { trials=trials[-(1:50),] ; trimmed_initiation = TRUE } # this prevents getting stuck because of an unlucky initiation

trials = rbind(trials,cbind(srsampsize=sqrt(tarNN),simulations))

loglik = function(xx) { sum(log( (1-trials[,"nullreject"]) + (trials[,"nullreject"]-.5)*2*pnorm(intercept_target+xx["beta"]*(trials[,"srsampsize"]-xx["X_target"])))) }

if(verbose) { print(trials[nrow(trials)-2:0,]) ; cat("\n") ; print(c("beta"=latest_beta, "X_target"=latest_estimate)) }

initial_value = loglik(c("beta"=latest_beta, "X_target"=latest_estimate))

if(initial_value!=Inf&initial_value!=-Inf&!is.na(initial_value))

{

mle <- optim(c("beta"=latest_beta, "X_target"=latest_estimate), fn=loglik, hessian = TRUE, control=list(fnscale=-1))

latest_beta = unname(mle$par["beta"])

older_estimate = latest_estimate

latest_estimate = unname(mle$par["X_target"])

if(det(mle$hessian)!=0) { se = sqrt(diag(solve(-mle$hessian)))["X_target"] } else {se = 10}

if(is.na(se)){se=NULL}

tarNN = pmax(7,latest_estimate+c(-1,1)*min(10,se)) # span a window of +- 1 SE except if SE too large, in increments of 10% of the number of existing data points, minimum trial size 49

tarNN = round(seq(tarNN[1],tarNN[2],length.out=max(50,round(nrow(trials)/10)))^2)

}

if(verbose) { cat("\n") ; print(tarNN); cat("\n") ; print(se) ; cat("\n") }

}

cat("sample size found : ", round(latest_estimate^2), " with ",nrow(trials), " simulated trials", "\n")

round(latest_estimate^2)

}

simulate_one_trial = function(NN)

{

DD = data.frame(yy = rnorm(NN,0,5), x1=0,x2=0,x12=0)

DD[(floor(NN/3)+1):(floor(NN*1/2)+0),"x1"]=1

DD[(floor(NN/2)+1):(floor(NN*2/3)+0),"x2"]=1

DD[(floor(NN*2/3)+1):(NN),"x1"]=1

DD[(floor(NN*2/3)+1):(NN),"x2"]=1

DD$x12= DD$x1*DD$x2

DD$yy = DD$yy+DD$x1

summary(glm("yy~x1+x2",data=DD))$coef

}

print( simulate_sampsize() )

simulate_one_trial = function(NN)

{

DD = data.frame(yy = rnorm(NN,0,5), x1=0,x2=0,x12=0)

DD[(floor(NN/3)+1):(floor(NN*1/2)+0),"x1"]=1

DD[(floor(NN/2)+1):(floor(NN*2/3)+0),"x2"]=1

DD[(floor(NN*2/3)+1):(NN),"x1"]=1

DD[(floor(NN*2/3)+1):(NN),"x2"]=1

DD$x12= DD$x1*DD$x2

DD$yy = DD$yy+DD$x1

summary(glm("yy~x1+x2+x12",data=DD))$coef

}

print( simulate_sampsize() )

simulate_one_trial = function(NN)

{

DD = data.frame(yy = rnorm(NN,0,5), x1=0,x2=0,x12=0)

DD[(floor(NN/4)+1):(floor(NN*1/2)+0),"x1"]=1

DD[(floor(NN/2)+1):(floor(NN*3/4)+0),"x2"]=1

DD[(floor(NN*3/4)+1):(NN),"x1"]=1

DD[(floor(NN*3/4)+1):(NN),"x2"]=1

DD$x12= DD$x1*DD$x2

DD$yy = DD$yy+DD$x1

summary(glm("yy~x1+x2",data=DD))$coef

}

print( simulate_sampsize() )

simulate_one_trial = function(NN)

{

DD = data.frame(yy = rnorm(NN,0,5), x1=0,x2=0,x12=0)

DD[(floor(NN/4)+1):(floor(NN*1/2)+0),"x1"]=1

DD[(floor(NN/2)+1):(floor(NN*3/4)+0),"x2"]=1

DD[(floor(NN*3/4)+1):(NN),"x1"]=1

DD[(floor(NN*3/4)+1):(NN),"x2"]=1

DD$x12= DD$x1*DD$x2

DD$yy = DD$yy+DD$x1

summary(glm("yy~x1+x2+x12",data=DD))$coef

}

print( simulate_sampsize() )

simulate_one_trial = function(NN)

{

DD = data.frame(yy = rnorm(NN,0,5), x1=0,x2=0,x12=0)

DD[(floor(NN*.4)+1):(floor(NN*.65)+0),"x1"]=1

DD[(floor(NN*.65)+1):(floor(NN*.90)+0),"x2"]=1

DD[(floor(NN*.9)+1):(NN),"x1"]=1

DD[(floor(NN*.9)+1):(NN),"x2"]=1

DD$x12= DD$x1*DD$x2

DD$yy = DD$yy+DD$x1

summary(glm("yy~x1+x2",data=DD))$coef

}

print( simulate_sampsize() )

simulate_one_trial = function(NN)

{

DD = data.frame(yy = rnorm(NN,0,5), x1=0,x2=0,x12=0)

DD[(floor(NN*.4)+1):(floor(NN*.65)+0),"x1"]=1

DD[(floor(NN*.65)+1):(floor(NN*.90)+0),"x2"]=1

DD[(floor(NN*.9)+1):(NN),"x1"]=1

DD[(floor(NN*.9)+1):(NN),"x2"]=1

DD$x12= DD$x1*DD$x2

DD$yy = DD$yy+DD$x1

summary(glm("yy~x1+x2+x12",data=DD))$coef

}

print( simulate_sampsize() )

simulate_one_trial = function(NN)

{

DD = data.frame(yy = rnorm(NN,0,2), x1=0,x2=0,x3=0,x4=0,x5=0)

fractional_matrix = matrix( c(0,0,0,1,1,

1,0,0,0,0,

0,1,0,0,1,

1,1,0,1,0,

0,0,1,1,0,

1,0,1,0,1,

0,1,1,0,0,

1,1,1,1,1

)

,ncol=5,byrow=T

)

DD[1:NN,c("x1","x2","x3","x4","x5")] = fractional_matrix[sample(1:8,NN,replace=T),]

DD$yy = DD$yy+DD$x1

summary(glm("yy~x1+x2+x3+x4+x5",data=DD))$coef

}

print( simulate_sampsize() )

simulate_one_trial = function(NN)

{

DD = data.frame(yy = rnorm(NN,0,2), x1=0,x2=0,x3=0,x4=0,x5=0)

DD[1:NN,c("x1","x2","x3","x4","x5")] = matrix(rbinom(NN*5,1,.5),ncol=5)

DD$yy = DD$yy+DD$x1

summary(glm("yy~x1+x2+x3+x4+x5",data=DD))$coef

}

print( simulate_sampsize() )

simulate_one_trial = function(NN)

{

DD = data.frame(yy = c(1,1,.7,.7), x1=c(1,1,1,0),x2=c(1,1,0,1),x3=c(1,0,1,1),x4=c(0,1,1,1))

DD = DD[sample(1:4,NN,replace=T),]

DD$yy = runif(NN)<DD$yy

summary(glm("yy~0+x1+x2+x3+x4",family=binomial(logit),data=DD))$coef

}

simulate_one_trial(700)

simulate_one_trial = function(NN)

{

DD = data.frame(yy = c(.99,.99,.7,.7), x1=c(1,1,1,0),x2=c(1,1,0,1),x3=c(1,0,1,1),x4=c(0,1,1,1))

DD = DD[sample(1:4,NN,replace=T),]

DD$yy = runif(NN)<DD$yy

summary(glm("yy~0+x1+x2+x3+x4",family=binomial(logit),data=DD))$coef

}

simulate_one_trial(700)

simulate_one_trial = function(NN)

{

aa = round(NN*(0:1+c(1,-1)*0.2857143))

bb = rbinom(2,aa,c(.7,.5))

prop.test(cbind(bb,aa-bb),correct=FALSE)$p.value

}

bpower(.7,.5,.2857143,alpha=0.05/7,461)

# with the original bpower function from package Hmisc the line is bpower(.7,.5,n1=round(.2857143*461),n2=round((1-.2857143)*461),alpha=.05/7)

aa = replicate(20000,simulate_one_trial(461))

mean(aa<.05/7)
